# Identification of Multiple Loci Associated with Social Parasitism in Honeybees

**DOI:** 10.1371/journal.pgen.1006097

**Published:** 2016-06-09

**Authors:** Andreas Wallberg, Christian W. Pirk, Mike H. Allsopp, Matthew T. Webster

**Affiliations:** 1 Department of Medical Biochemistry and Microbiology, Science for Life Laboratory, Uppsala University, Uppsala, Sweden; 2 Department of Zoology and Entomology, University of Pretoria, Pretoria, South Africa; 3 Plant Protection Research Institute, Agricultural Research Council, Stellenbosch, South Africa; University of California, Berkeley, UNITED STATES

## Abstract

In colonies of the honeybee *Apis mellifera*, the queen is usually the only reproductive female, which produces new females (queens and workers) by laying fertilized eggs. However, in one subspecies of *A*. *mellifera*, known as the Cape bee (*A*. *m*. *capensis*), worker bees reproduce asexually by thelytoky, an abnormal form of meiosis where two daughter nucleii fuse to form single diploid eggs, which develop into females without being fertilized. The Cape bee also exhibits a suite of phenotypes that facilitate social parasitism whereby workers lay such eggs in foreign colonies so their offspring can exploit their resources. The genetic basis of this switch to social parasitism in the Cape bee is unknown. To address this, we compared genome variation in a sample of Cape bees with other African populations. We find genetic divergence between these populations to be very low on average but identify several regions of the genome with extreme differentiation. The regions are strongly enriched for signals of selection in Cape bees, indicating that increased levels of positive selection have produced the unique set of derived phenotypic traits in this subspecies. Genetic variation within these regions allows unambiguous genetic identification of Cape bees and likely underlies the genetic basis of social parasitism. The candidate loci include genes involved in ecdysteroid signaling and juvenile hormone and dopamine biosynthesis, which may regulate worker ovary activation and others whose products localize at the centrosome and are implicated in chromosomal segregation during meiosis. Functional analysis of these loci will yield insights into the processes of reproduction and chemical signaling in both parasitic and non-parasitic populations and advance understanding of the process of normal and atypical meiosis.

## Author Summary

Honeybee colonies are mainly composed of female worker bees that are unable to lay eggs and reproduce; a task that is performed by a single queen bee. However, in a subspecies of the Western honeybee (*Apis mellifera*) known as the Cape bee (*A*. *m*. *capensis*), worker bees can lay eggs produced by an abnormal form of meiotic cell division known as thelytoky. These eggs are produced asexually and develop into female worker bees, which are then able to reproduce in the same way and hence to propagate this trait. Cape bee workers also display a variety of characteristics that enable them to invade foreign colonies, reproduce and feed off their resources, a behavior known as social parasitism. The genetic basis of these traits is unknown. Here we compare whole genome sequences of Cape bees with other honeybee subspecies and identify the genes that likely control this reproductive strategy. Although Cape bees are extremely genetically similar to other African bees, we identify genetic variants that make it possible to unambiguously distinguish them. In contrast to previous studies, our results suggest that multiple genes are involved in social parasitism, including those involved in hormonal signaling that may cause worker bees to activate their ovaries and those involved in chromosomal segregation, which may cause the switch between normal meiosis and thelytoky. Our results give insights into how genetic variants that facilitate asexual reproduction are able to invade a sexually reproducing population.

## Introduction

In most colonies of the honeybee *Apis mellifera*, the queen is the only reproductively active female and it produces pheromones that inhibit ovary activation in the workers [1,2], keeping them sterile. Under some circumstances, particularly when the colony lacks a queen, workers may activate their ovaries and lay unfertilized haploid eggs via a process called arrhenotokous parthenogenesis. Due to the haplodiploid sex determination of honeybees (and other hymenoptera) these haploid eggs develop into male drones. The Cape bee (*Apis mellifera capensis* Escholtz) is a subspecies of the honeybee *Apis mellifera* that inhabits the far south of South Africa [3–5]. The Cape bee differs from other honeybee subspecies in that unmated females, particularly workers, frequently produce female progeny that develop from unfertilized eggs through thelytokous parthenogenesis. Thelytoky is a form of meiosis whereby two daughter nuclei that form in the oocyte fuse to produce a viable diploid egg [6]. This abnormal mechanism of restoring diploidy in unfertilized eggs occurs only occasionally in normally arrhenotokous populations (<1% of eggs) but is the norm in reproducing Cape bee workers (>99% of eggs) [7].

The Cape bee exhibits a number of other traits that distinguish it from other subspecies and lead it to behave as a social parasite, whereby workers invade the nests of foreign colonies, reproduce and utilize their resources. Cape bee workers have significantly larger ovaries that are more readily activated and contain more egg-producing ovarioles than worker bees of other subspecies (10–20 vs. 1–5) [3,8]. Parasitic egg-laying Cape bee workers produce queen pheromones, allowing them to assert reproductive dominance over other workers [9] and have a significantly increased lifespan of 3–5 months compared to 6 weeks in non-parasitic workers [10]. These traits likely increase the evolutionary advantage of parthenogenesis, which allows this mode of selfish reproduction to become established. As with other honeybee subspecies, Cape bee workers rarely reproduce if a queen is present (~0.1% of drones are produced from worker-laid eggs in a normal honeybee colony [11]). However, during reproductive swarming in Cape bees as many as 10% of young workers are produced by egg-laying workers [12]. In between 40% to 60% of young queens produced during swarming are the offspring of workers [13,14].

The genetic basis of thelytoky and its associated behavioral and physiological traits in the Cape bee is still largely unclear. Asexual reproduction by thelytokous parthenogenesis is relatively common in other species of Hymenoptera [15]. In the parasitoid wasp *Lysiphlebus fabarum*, where different forms exhibit arrhenotokous or thelytokous parthenogenesis, a single locus determines this difference [16]. Thelytokously reproducing individuals of this species are all homozygous for an allele at a particular microsatellite locus, which is very rare in arrhenotokously reproducing individuals. In the Cape bee, the genetic basis of thelytoky has been investigated using backcrosses of hybrids formed by crossing *A*. *m*. *capensis* with *A*. *m*. *carnica* [17]. The pattern of segregation in such crosses is generally consistent with inheritance of thelytoky being determined by a single recessive locus, which has been termed *thelytoky (th)*, although more complex modes of inheritance incorporating more loci are also compatible with this inheritance pattern. This putative locus has been mapped to an interval on chromosome 13 [18] and a 9 bp deletion in this region has been proposed as the causative variant [19], however, a subsequent study failed to replicate this association [20] and detected the presence of the deletion in populations of other subspecies where thelytoky is absent (see Materials and Methods for more details).

Cape bees are genetically extremely similar to other African subspecies of honeybees, including *A*. *m*. *adansonii* and *A*. *m*. *scutellata* [21] suggesting a recent common ancestor and/or high levels of gene flow. Social parasitism in Cape bees should therefore represent a recently derived adaptation that has likely experienced positive selection. The distribution of Cape bees shows strong concordance to the biodiverse Fynbos ecoregion of South Africa, which suggests that the traits specific to the Cape bee are evolutionarily advantageous in this region [5]. Cape bee workers reproduce by thelytokous parthenogensis in the absence of a queen, during reproductive swarming or when invading the colony of a different subspecies. The propensity to do this is a trait that is fixed or close to fixation in the Cape bee population [3,22]. However, in their native range most reproduction in Cape bees is sexual, so patterns of genetic diversity therefore resemble those in other sexual outcrossing populations [21]. We therefore expect genetic variants responsible for traits associated with social parasitism to be located in regions of high differentiation with populations of other subspecies and associated with signals of selection in Cape bees.

What is the genetic basis of social parasitism in Cape bees? Social parasitism is facilitated by a range of different traits related to ovary development, behaviour and abnormal meiosis. Does a single *thelytoky* locus act as a “master switch” to enable this suite of phenotypes as previously suggested [17–19], or are each of these traits determined by an independent set of loci? To what extent are Cape bees genetically distinct from other African honeybee populations? Was the emergence of social parasitism associated with increased positive selection in the Cape bee population?

Here we use population-scale genome sequencing to address these questions. We developed a method to detect selection in Cape bees compared to other African populations that combines detecting SNPs with high differentiation between populations with long haplotype tests of selection [23,24]. Here we apply this approach to a dataset of 10 whole genome sequences of Cape bees, sampled from locations were workers are known to produce nearly exclusively female offspring [25], compared to 20 genome sequences of other sub-Saharan African honeybees that do not reproduce thelytokously. We detect a number of genomic regions with strong signals of selection in the Cape bee, which are likely to underlie the unique suite of traits that facilitate social parasitism in this subspecies.

## Results

### Low levels of genetic differentiation in Cape bees

**Fig 1:**
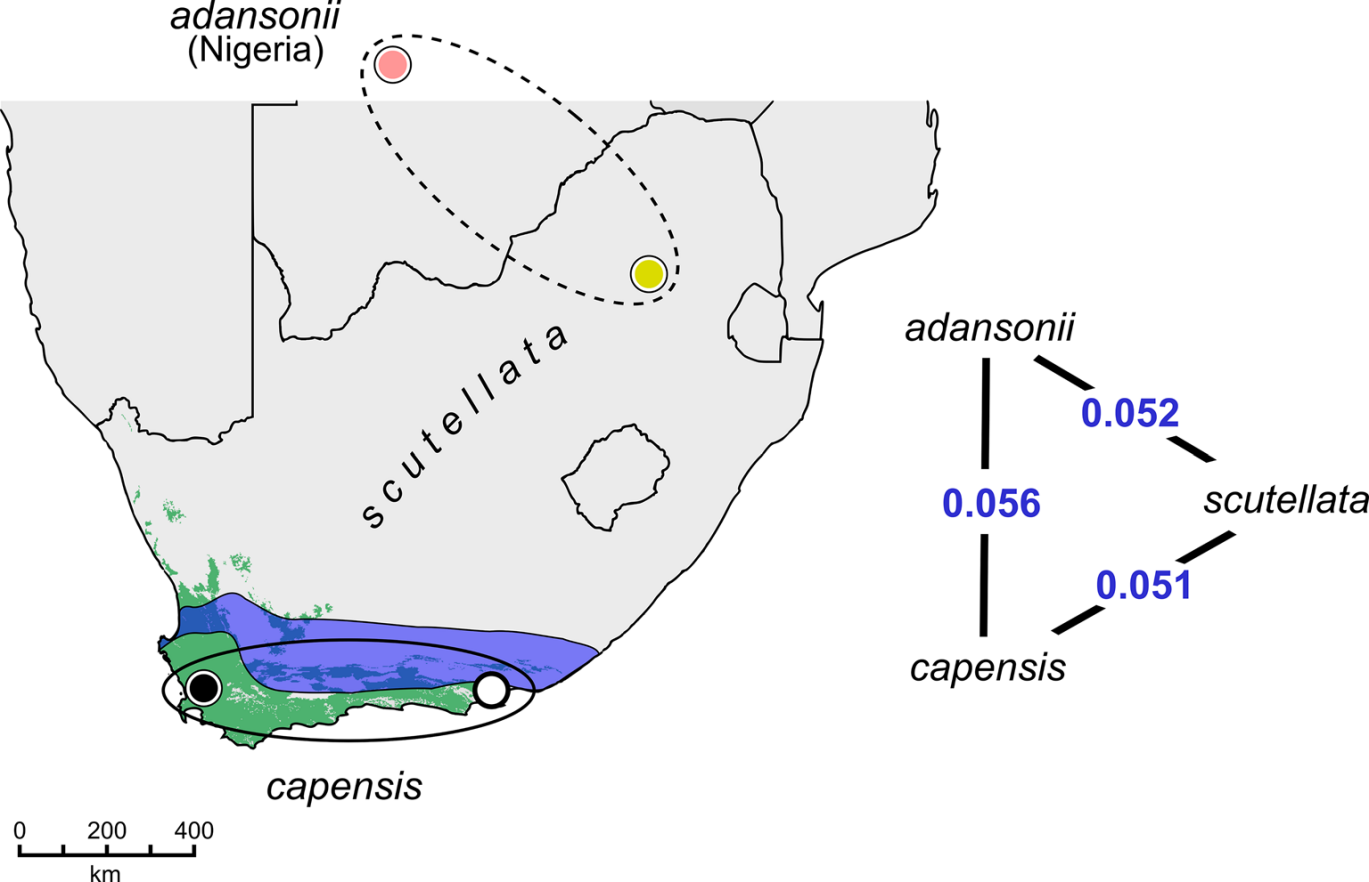
Geographical location of population samples. The thelytokous and parasitic Cape bee *Apis mellifera capensis* (lower ellipse) inhabits the Fynbos ecoregion (green) of South Africa. It was sampled from the western Fynbos (Stellenbosch, Cape Town; black circle) and eastern Fynbos (near Kragga Kamma Game Park, Port Elizabeth; white circle) and compared to two arrhenotokous and non-parasitic African populations (upper dashed ellipse). *A*. *m*. *scutellata* is widespread throughout of the Central Plateau and was sampled in the Pretoria region (yellow circle). A transitional zone with mixed phenotypes exists between the two subspecies (blue region). *A*. *m*. *adansonii* bees (pink) were sampled in Kaduna state, Nigeria (outside map). The schematic triangle specify the genome-wide levels of divergence between the three populations (*F*_ST_ estimator of Reynolds *et al*. [104]).

We aimed to identify genetic variants associated with strong differentiation between the Cape bee and other African bees due to selection in the Cape bee in favor of traits involved in social parasitism. We analyzed whole genome sequences from 10 Cape bees (*A*. *m*. *capensis*) sampled from two localities in southern South Africa: Port Elizabeth to the east (n = 5) and Cape Town to the west (n = 5). We compared these with sequences from two other African subspecies: *A*. *m*. *scutellata* collected in Pretoria (n = 10) and *A*. *m*. *adansonii* collected in Nigeria (n = 10; Fig 1). Genetic differentiation between these three African populations ranges between *F*_ST_ = 0.051–0.056 (Fig 1) and *A*. *m*. *scutellata* appears to be nearly equidistant genetically between the Cape bee in the south and *A*. *m*. *adansonii* in the north. This level of divergence is consistent with a recent common ancestor and/or pervasive gene flow among sub-Saharan populations.

We considered the geographically widespread *A*. *m*. *scutellata* and *A*. *m*. *adansonii* populations as a single African background population and attempted to identify alleles under selection in the Cape bees compared to this background population. *F*_ST_ between the Cape bee population and the background population is 0.044 and the allele frequency spectrum is dominated by variants segregating at similar frequencies: 93.3% of SNPs (n = 5.89 x 10^6^) have *F*_ST_ values below 0.1, whereas only 0.33% of SNPs (n = 20,460) have *F*_ST_ values above 0.3 (S1A Fig).

There are 45 SNPs fixed for different variants between the Cape bees (n = 10) and other African bees (n = 20), which are associated with one gene accession on chromosome 1 corresponding to *Ethr* (ecdysis-triggering hormone receptor) and three predicted genes on chromosome 11 (hypothetical proteins GB44917, GB45238 and GB45239; S1 Table). When the two Cape bee subpopulations are compared against other African populations separately, we observed 423 such fixed SNPs in the (western) Cape Town population (n = 5), which are associated with 38 gene accessions (S1B Fig; S1 Table). However, in the (eastern) Port Elizabeth population (n = 5), we detect only 60 fixed SNPs (associated with five gene accessions). *F*_ST_ between the African background population and the Cape Town population (0.070) is slightly higher than the African background population and the Port Elizabeth population (0.063). The Cape Town population also has a general excess of high *F*_ST_ variants. Between the two comparisons against the African background population, the analysis with the Cape Town subpopulation include 70% or more of the variants segregating at *F*_ST_>0.5 (S1B and S1C Fig). Colonies with the entire suite of Cape-bee-specific phenotypes are more common in the Cape Town region, whereas they occur less frequently in Port Elizabeth [3,22] and hence the higher number of fixed SNPs in the Cape Town region may reflect additional variants associated with these phenotypes.

### Evidence of increased levels of selection in Cape bees

Natural selection is expected to drive adaptive genetic variants towards fixation together with linked neutral variation, generating a pattern of increased population differentiation and higher haplotype homozygosity around selected variants [26]. We sought to identify genetic variants with high genetic differentiation due to recent selection specifically in the Cape bee population, reasoning that such variants likely underlie traits associated with social parasitism. To do this, we integrated SNP-level *F*_ST_ estimates with haplotype structure analyses. We implemented the population branch statistic (PBS) [27] to detect genomic regions that diverged rapidly on the Cape bee lineage compared to other African populations. We also scanned genome variation for genetic variants linked to long haplotypes in the Cape bee population using the cross-population extended haplotype homozygosity statistic (XP-EHH) [23] (implemented in the program *selscan* [28]).

For both PBS and XP-EHH estimates, values greater than zero are associated with selection signals in the Cape bees, whereas negative values indicate selection in the African background population. Across all SNPs in the dataset we find that the distribution of these values are centered around zero (mean XP-EHH = 0.007; mean PBS = 0.039), but that the most extreme XP-EHH and PBS scores are biased towards stronger signals in the Cape bees rather than the background population: for the PBS, the upper and lower 99.9% percentiles of the empirical distributions are 1.27 and -0.34, respectively, and for the XP-EHH theses values are 4.12 and -3.46 (S2A and S2B Fig). Low *F*_ST_ SNPs (*F*_ST_<0.3; >99% of SNPs) appear to be largely unbiased between the two groups with respect to the PBS and XP-EHH statistics (scores are close to zero), whereas high *F*_ST_ SNPs tend to have high values of these statistics in Cape bees: the average PBS and EHH scores of SNPs with *F*_ST_>0.85 are within the top 0.5% of these statistics (p<0.01, bootstrap; S2C–S2E Fig). Moreover, variants segregating at the highest frequency differences are not randomly distributed but tend to be clustered in regions with the strongest linked signatures of selection: regional *F*_ST_ drops to the top 1% level after about 50kbp for SNPs at *F*_ST_>0.9, compared to about 20kbp for SNPs at *F*_ST_ = 0.8–0.9, and these patterns decay similarly for PBS and XP-EHH (S3A–S3C Fig).

**Fig 2:**
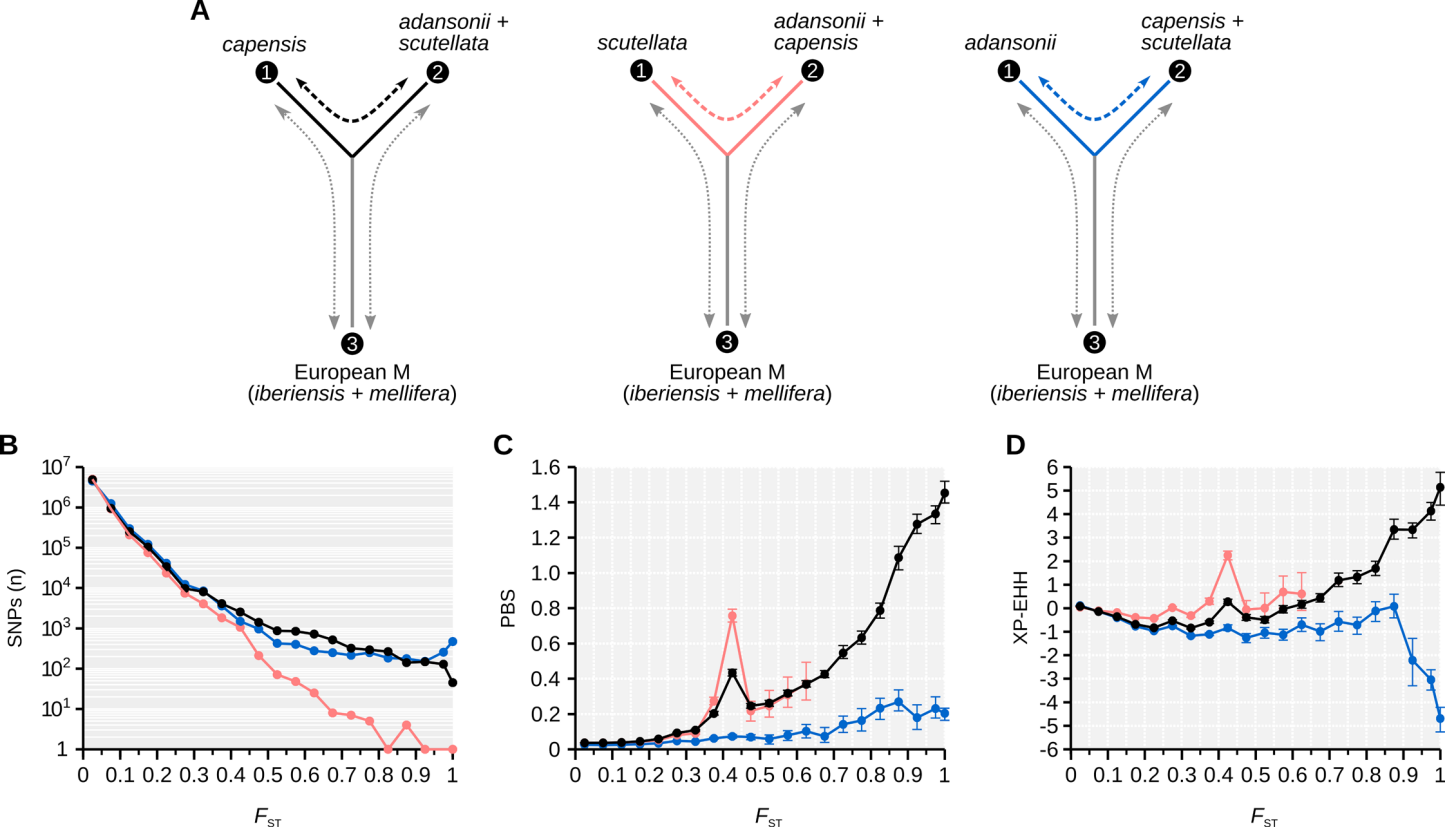
Selection signals are stronger in the Cape bees than any of the other two subspecies. The fixation index (*F*_ST_), Population Branch Statistic (PBS) and Cross-Population Extended Haplotype Homozygosity (XP-EHH) were computed for every SNP segregating between each African subspecies and a combined population consisting of the two remaining subspecies. (A) The structure of the three experiments. The main *F*_ST_ value was computed between each subspecies and the combined population (dashed line; population 1 (n = 10) and 2 (n = 20); *F*_ST1⟷2_) for every SNP. *F*_ST1⟷2_ was also estimated across 1kbp windows and between each population and a distantly related European outgroup (grey dashed lines; *F*_ST1⟷3_ and *F*_ST2⟷3_) in order to estimate the local PBS associated with every SNP. The first figure represents the main scan, i.e. comparing the Cape bees against the other African and European bees (black). The middle figure compares *scutellata* against the other bees (pink). The last figure compares *adansonii* against the other bees (blue). (B) The number of SNPs counted for *F*_ST_ bins of 0.05 for each comparison. Colors as in (A). (C) The mean PBS value for every *F*_ST_ bin of 0.05. Positive values identify divergence associated with population 1. Outlier variants with *F*_ST_>0.8 are most strongly biased towards divergence in population 1 when the Cape bees are taken as this population. 95% confidence intervals were computed from 200 bootstrap replicates. Colors as in (A). Data points and confidence intervals plotted for bins with at least 10 SNPs. (D) The mean XP-EHH value for every *F*_ST_ bin of 0.05. Positive values identify long haplotypes in population 1 relative to population 2, whereas negative values represent the opposite. Outlier variants with *F*_ST_>0.8 are most strongly biased towards long haplotypes in population 1 when the Cape bees are taken as this population. 95% confidence intervals were computed from 200 bootstrap replicates. Colors as in (A). Data points and confidence intervals plotted for bins with at least 10 SNPs.

These analyses suggest that more variants are associated with selection signals in the Cape bee population compared to the background population. Many of these variants may underlie the unique set of derived characteristics associated with thelytokous parthenogenesis and social parasitism in the Cape bee. In order to test the robustness of these observations, we performed two additional reciprocal comparisons: one comparing *A*. *m*. *scutellata* to the remaining two African populations, and the other comparing *A*. *m*. *adansonii* to the remaining populations. For each of the comparisons, we estimated *F*_*ST*_, PBS, and XP-EHH statistics in the same way as in the original genome scan (Fig 2A). The distribution of *F*_ST_ in the additional comparisons has fewer peaks than in the Cape Bee, indicating fewer signals of selection specifically in these populations (SNPs with *F*_ST_>0.8 occur on 13 chromosomes comparing Cape bees against the other African populations, but only on one and six chromosomes respectively in the corresponding *A*. *m*. *scutellata* and *A*. *m*. *adansonii* scans; S4A–S4C Fig). In some cases we detected partially overlapping signals (for instance around position 14,000,000 on chromosome 1) such that some accessions had outlier SNPs associated with more than one subspecies. However, among the gene candidates identified (see section *Characterization of candidate genes* below), 95% and 99% of the accessions had higher overall PBS signals in the Cape bees than in *A*. *m*. *scutellata* or *A*. *m*. *adansonii*, respectively, consistent with an excess of uniquely derived variants and stronger selection in the Cape bees in most cases.

In the comparison with *A*. *m*. *scutellata*, there are extremely few SNPs with high *F*_ST_ (*F*_ST_>0.80; 7 SNPs, compared to 730 SNPs in the Cape bee comparison). These SNPs have mildly elevated PBS values, but do not appear to be associated with signals of selection in *A*. *m*. *scutellata* according to the XP-EHH test (Fig 2B–2D). In the comparison with *A*. *m*. *adansonii*, a large number of SNPs have high *F*_ST_, but they are restricted to a few genomic regions. It is likely that many of these SNPs lie within regions of structural variation such as inversions that differ in frequency in the *A*. *m*. *adansonii* population, as high-*F*_ST_ SNPs are restricted to a few large blocks in this comparison. For instance, 84% of 1,241 SNPs with *F*_ST_>0.8 are located to two 0.5Mb blocks on chromosome 7 and a 1Mb block on chromosome 11 (S4C Fig). However, PBS is only marginally biased towards increased divergence in *A*. *m*. *adansonii* at high-*F*_ST_ SNPs in this comparison. Furthermore, XP-EHH values are below zero, indicating that differentiation at these variants is more strongly associated with selection in the background population (*A*. *m*. *capensis* and *A*. *m*. *scutellata*) (Fig 2B–2D). Taken together, these observations confirm that despite similar levels of genetic differentiation overall, the number of genetic variants that are highly differentiated due to selection in the Cape bee population is much higher than in other African populations. These genetic variants are therefore likely to underlie the suite of traits connected to social parasitism.

Since the western Cape Town and eastern Port Elizabeth Cape bee subpopulations showed different levels of fixation against the background population, we also queried these two populations separately for linked signals of haplotype divergence and diversity using three sets of outlier SNPs: those identified as 0.1% outliers for *F*_ST_ against the background population and with minimal evidence for extended haplotype homozygosity in the Cape bees (XP-EHH>0) across the complete dataset and those identified for the same criteria, but for each subpopulation individually. The associated haplotype-level signals are consistently stronger in the western Cape Town subpopulation compared with the Port Elizabeth subpopulation for all three sets (S5A–S5C Fig), congruent with greater fixation of Cape-bee-specific traits in the western region [3].

### Identification of genetic variants under selection

**Fig 3:**
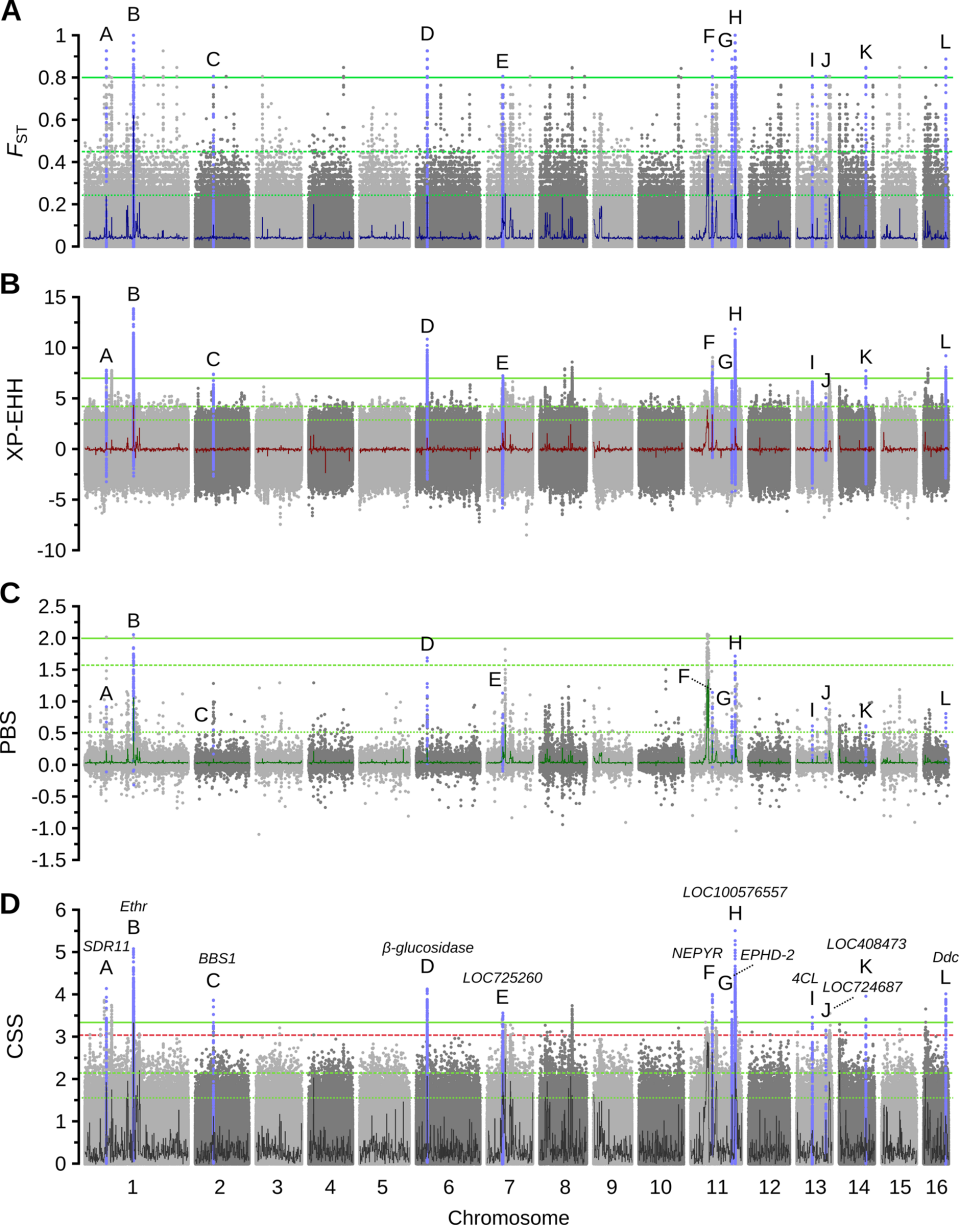
A genome-wide scan for selection identifies several candidate loci in the Cape bee genome. Selection statistics associated with every SNP segregating between the Cape bees (*A*. *m*. *capensis*) and the African background population (*scutellata* + *adansonii*) (n = 6.2×10^6^). (A) The Fixation index (*F*_ST_). Dark blue line is *F*_ST_ across 100kbp non-overlapping windows. Green lines are the 99.99% percentile (solid line; *F*_ST_>0.44; n = 624), the 99.9% percentile (dashed line; *F*_ST_>0.80; n = 6245) and the 99.5% percentile (dotted line; *F*_ST_>0.24; n = 31,226). Highlighted in light blue are all SNPs associated with the 12 candidate sweeps labeled from A to L (Table 1). See (D) below. (B) The Cross Population Extended Haplotype Homozygosity (XP-EHH) score of every SNP. Dark red line is the average XP-EHH across 100kbp non-overlapping windows. Green lines are the 99.99% percentile (solid line; XP-EHH>7.03; n = 622), the 99.9% percentile (dashed line; XP-EHH>4.11; n = 6224) and the 99.5% percentile (dotted line; XP-EHH>2.89; n = 31,120). Highlighted SNPs as in (D). (C) Haplotype divergence between the Cape bees, *scutellata* + *adansonii* and a European outgroup as measured using the Population Branch Statistic (PBS) for 1kbp non-overlapping windows. Dark green line is the average PBS across 100kbp non-overlapping windows. Green lines are the 99.99% percentile (solid line; PBS>1.99; n = 19), 99.9% percentile (dashed line; PBS>1.28; n = 190) and the 99.5% percentile (dotted line; PBS>0.51; n = 1946). Highlighted windows overlap with accessions in (D). (D) The composite selection score (CSS) of every SNP, based on the joint fractional rank for both *F*_ST_ and XP-EHH estimates of each SNP. Black line is the CSS computed from the fractional ranks of *F*_ST_ and XP-EHH for 100kbp non-overlapping windows. Green lines are the 99.99% percentile (solid line; CSS>3.33; n = 624), 99.9% percentile (dashed line; CSS>2.26; n = 6245) and the 99.5% percentile (dotted line; CSS>1.62; n = 31,226). Red dashed line at the minimum CSS level of the top 1000 SNPs within 8kbp from gene bodies (CSS>3.04; n = 1000; 97 accessions; S3 Table). 25 of the 97 accessions have at least one SNP with allele frequency differences above the 99.99% percentile (*F*_ST_>0.8) and are located into 12 putative selective sweeps (A–L; all SNPs associated with each accession are highlighted in blue; Table 1). The three sweeps H (uncharacterized protein LOC100576557; chromosome 11), B (Ethr; chromosome 1) and D (*β*-glucosidase; chromosome 6) have the SNPs with the highest CSS.

In order to identify variants specifically under selection in Cape bees, we estimated a composite selection score (CSS) [24] based on *F*_ST_ and XP-EHH scores (see Materials and Methods). We did not include PBS in this score as the magnitude of PBS is expected to correlate with *F*_ST_ under neutrality. However, a strong association between *F*_ST_ and XP-EHH is not expected according to the results of coalescent simulations [29]. In our dataset, we find a positive and significant but weak correlation between the absolute values of the XP-EHH statistic and *F*_ST_ (R^2^ = 0.07; p<1e^-5^), consistent with the simulated data. The genome-wide distribution of *F*_ST_, XP-EHH and PBS are shown in Fig 3A–3C. The distribution of CSS scores of all six million SNPs is shown in Fig 3D. We found that SNPs with the top 2000 CSS scores were significantly enriched within coding sequence by a factor of 2.3x (p<1e^-5^; Fischer’s exact test) compared to the expected. Likewise, SNPs are enriched within UTR regions and introns by 1.9x and 1.3x respectively (p<1e^-5^ in both cases). Taken together, this enrichment indicates that extreme signals are centered on gene bodies and that coding variants in particular are more commonly targets of selection than non-coding intergenic ones, which are underrepresented in the top SNPs (0.57x the expected amount; p<1e^-5^). Due to the association of outlier SNPs with gene bodies and the difficulty in characterizing the functional relevance of intergenic SNPs in gene deserts, we focused downstream analyses on outlier variants located close to genes.

We selected a conservative set of top-ranking SNPs to produce a set of putative candidate genes. Among the top 1000 SNPs in the full dataset, 873 SNPs (87.3%) occur within 8kbp of a gene, compared to 72% overall, a gene-centric overrepresentation (p<1e^-5^) consistent with the enrichment reported above. We selected these 873 SNPs plus the next 137 for analyses based on the top 1000 SNPs located within 8 kb of a gene (CSS = 3.04–5.52; 0.022% of all SNPs <8 kb from a gene; S2 Table). These make up 87.7% of the top 1140 SNPs. The average *F*_ST_ of these SNPs is 0.67, indicating that although they were selected on the basis of both *F*_ST_ and XP-EHH, they typically have high allele frequency differences between the Cape bees and the African background population. Indeed, among these SNPs, the average frequency of the major allele in the Cape bees is about 90%, compared to only 20% in the African background and 18% in the European sample used as outgroup for the PBS (S2 Table). The frequency of the major Cape bee allele was below 0.2 in 63% and 80% of SNPs in the African background and European populations, respectively. This demonstrates that putative candidate variants for social parasitism are typically rare outside of the Cape bee population. We next queried the 1000 SNPs against the current honeybee gene annotation models and found them to be located to peaks that associate with 97 gene accessions (S3 Table). These gene accessions have average CSS levels across the full gene bodies within the top 1% percentile of genes (CSS_97_ = 1.654 vs CSS_1%_ = 1.546; S6 Fig).

We queried the *Drosophila* orthologues (n = 61) of this gene set for enriched gene ontology terms using the GOrilla platform [30] and detected 16 significantly enriched biological processes and molecular functions after controlling for multiple testing [31](p<0.05; S7A and S7B Fig). We used the REVIGO tool [32] to reduce redundancy in the list of GO terms, removing 4 terms (S4 Table). Among the remaining GO-terms, we detect significant enrichment of genes associated with ecdysteroid metabolic processes (GO:0042445; n = 5; p = 6.15e^-3^; S4 Table). The genes we detected within this category include orthologs of three glucose-methanol-choline (GMC) oxidoreductases (*GMCOX4/6/9*) located in a conserved cluster on chromosome 1 [33], and also *Npc1a* and *foxo* (S4 Table; S7A Fig). Ecdysteroid signaling is a hormonal process that is correlated with development and ovary maturation in honeybees [34–36]. The corresponding top 1000 SNPs from the two other reciprocal comparisons (*scutellata* vs the rest and *adansonii* vs the rest) did not reveal any significantly enriched biological process GO terms.

It is possible that genes associated with particular biological functions may be under selection but do not have clearly identifiable outlier variants under our criteria. In order to address this question, we analyzed a ranked list of all accessions with *Drosophila* orthologues (n = 6,253). We used the gene-wide average CSS scores to rank all genes and provided the list to GOrilla to perform analyses of enriched GO terms among top accessions while controlling for multiple testing. We did not detect any significantly enriched GO term with FDR-corrected p<0.05 using this method. To further address whether selection may be targeting genes specifically associated with meiosis and abnormal chromosome segregation (i.e. thelytoky candidates), we collected all genes associated with GO terms related to meiosis or chromosome segregation. Genes of GO terms matching key words “meiotic” or “meiosis” (56 GO-terms; 126 genes) and “segregation” (10 GO-terms, 74 genes) were compiled into a meiosis-list. Each gene was counted only once despite the number of terms it was associated with, resulting in a list of 161 genes. In the same way, we compiled a list of 55 ecdysteroid-related genes (i.e. ovary development candidates), associated with GO terms matching “ecdysteroid” (10 GO-terms; 20 genes) or “ecdysone” (12 GO-terms, 41 genes; ecdysone is an ecdysteroid hormone). There is only a single shared gene between the two lists, so effects of multiple testing should be expected to be negligible. The average CSS score among all honeybee accessions is 0.383. The mean CSS of the “meiosis-genes” is 0.377. After randomly sampling 161 genes across the genome 2000 times we find that this minor decrease of about 1.5% is not significant at the 0.05 level. For the “ecdysteroid-genes” the mean CSS is 0.410, a small increase of about 7% but not significant at the 0.05 level using the same resampling procedure.

These alternative GO analyses fail to detect broad patterns of selection on particular biological functions. For instance, while we find that some of the clearest signals of selection based on outlier variants are associated with a small subset of genes involved in ecdysteroid metabolism, we do not find evidence for widespread selection signals across all genes associated with ecdysteroid metabolism. Taken together, the results of the GO analyses are consistent with a scenario in which selection in the Cape bees has targeted a restricted set of loci (i.e. <1% of genes) with diverse functions, rather than broadly affecting genes with similar functions across the whole genome.

### Characterization of candidate genes

We further filtered the set of 97 candidate genes down to the subset of 25 accessions that had at least one top CSS SNP with an *F*_ST_>0.8 (n_SNPs_ = 393; Table 1). At these SNPs, the average frequency of the major allele in the Cape bees was 92%, but only 1.4% or 1.9% in the African background or European samples, respectively (S2 Table). The frequency of the major Cape bee allele was found to be below 0.2 at >97% of these SNPs in either of the two populations. These 25 genes therefore contain the most highly differentiated variants with signals of selection between the Cape bees and the other African bees and those variants are also typically very rare also outside of Africa. Out of the original top 1000 SNPs, 762 SNPs are associated with these 25 genes and the average CSS score across these genes are within the top 0.5% of all genes in the genome (CSS_25_ = 2.175 vs CSS_0.5%_ = 1.912; S6 Fig), indicating that these genes have comprehensive signals of selection. Of these 25 accessions, 16 genes belong to the set of 38 accessions with fixed variants between the western Cape Town subpopulation and African background population and 4 genes belong to the set of 5 with fixed variants between the eastern Port Elizabeth and the background population. These are listed in Table 1 and highlighted in blue in Fig 3A–3D (see S8 Fig for candidate gene plots). The top 762 SNPs associated with the 25 genes include 21 non-synonymous variants, affecting the coding sequence of 11 genes. Some of the 25 genes are located close together such that selection signals appear to extend across more than one accession. From manually inspecting these data we assigned these 25 genes into 12 distinct putative selective sweeps with strong evidence of selection in the Cape bee, including highly differentiated SNPs, which we labeled as A–L according to their location in the genome (Table 2). Corresponding plots of XP-EHH, *F*_ST_, PBS, and the combined CSS around these sweeps are shown in S8 Fig. The three putative selective sweeps with highest CSS values are on chromosomes 11 (locus H), 1 (locus B), and 6 (locus D) and overlap with 14 of the accessions, with outlier signals spanning at least three genes each.

**Table 1:** Gene candidates in the vicinity of selection signals.

Accession	Locus	Product	Outlier SNPs	CSS	Sweep region
GB40769	LOC412458	Dehydrogenase/reductase SDR family member 11-like	29	3.007	A
GB46502	LOC724680	Uncharacterized protein LOC724680	1	1.795	B
GB46426	LOC100578708	Protein star-like	8	2.469	B
GB46500	Ethr	Ecdysis triggering hormone receptor	254	3.299	B
GB46498	-	-	24	3.122	B
GB46427	LOC409096	Uncharacterized protein LOC409096	59	2.820	B
GB46428	-	-	3	1.424	B
GB46429	Ebony	Ebony	15	2.378	B
GB50742	LOC102655146	Bardet-biedl syndrome 1 protein-like	10	1.456	C
GB54485	LOC413043	Udp-glucuronosyltransferase 1-3-like	11	2.586	D
GB54461	-	-	3	2.559	D
GB54486	LOC411978	β-glucosidase	42	3.027	D
GB54460	LOC725017	Udp-glucuronosyltransferase 2b13-like	10	1.978	D
GB54634	LOC725260	Uncharacterized LOC725260	17	1.357	E
GB43519	LOC411614	Neuropeptide y receptor-like	20	2.063	F
GB44981	LOC100576773	-	2	1.655	G
GB44980	LOC409260	Epidermal retinol dehydrogenase 2-like	17	2.092	G
GB44917	-	-	167	2.293	H
GB45238	-	-	3	3.573	H
GB45239	LOC100576557	Uncharacterized protein LOC100576557	26	3.237	H
GB40077	LOC726040	Probable 4-coumarate—coa ligase 3-like	4	1.235	I
GB49919	LOC724687	Uncharacterized LOC724687	3	1.722	J
GB52756	LOC408474	Apyrase	1	0.863	K
GB52757	LOC408473	Uncharacterized LOC408473	7	0.855	K
GB45973	Ddc	Dopa decarboxylase	26	1.504	L

**Table 2:** Characteristics of sweep regions containing gene candidates.

Region	Linkage group	Core SNP position (bp)	Δ*π* selected haplotype (<0.01cM; %)	Length (cM)	Length (bp)	Approximate start position	Approximate stop position
A	1	6,188,694	-87	0.075	5,500	6,184,000	6,194,000
B	1	14,026,609	-71	0.075	20,500	14,021,000	14,060,000
C	2	5,240,333	-85	0.025	1,500	5,238,000	5,241,000
D	6	3,183,289	-82	0.125	11,500	3,171,000	3,195,000
E	7	4,547,937	-86	0.045	9,500	4,539,000	4,559,000
F	11	6,285,044	-59	0.025	5,500	6,279,000	6,290,000
G	11	11,923,867	-94	0.075	10,500	11,906,000	11,928,000
H	11	12,862,728	-83	0.115	10,500	12,852,000	12,872,000
I	13	4,532,630	-95	0.055	3,500	4,529,000	4,535,000
J	13	8,458,545	-86	0.025	5,500	8,452,000	8,464,000
K	14	7,726,445	-94	0.035	1,500	7,724,000	7,727,000
L	16	6,399,173	-91	0.145	5,500	6,393,000	6,405,000

The three SNPs with the highest CSS scores in the dataset are located in locus H on chromosome 11 (~40kbp wide), which contains 36 of the 45 fixed SNPs in the Cape bee sample relative to the African background population. The CSS scores are centered on the gene accession GB45239, which contains the non-synonymous SNP with the highest selection score in the dataset (CSS = 4.07). This gene is not characterized in the honeybees but has peptide sequence similarity to a structural maintenance of chromosomes (SMC) domain (COG1196; p = 6.82e^-04^; TIGR02168; p = 2.43e^-03^) in the NCBI Conserved Domain Database [37]. The role in chromosome integrity may indicate that this gene is involved in thelytokous meiosis in the Cape bee.

Locus B on chromosome 1 is ~110kbp wide and encompasses 7 genes. However, the 9 fixed SNPs are centered at an intron of the *Ethr* gene, which codes for the receptor for the neuropeptide ecdysis-triggering hormone (ETH). ETH is essential for initiating ecdysis but also regulates juvenile hormone (JH) synthesis in insects [38,39]. Notably, the region also spans genes coding for a *StAR*-like protein, an intracellular cholesterol transporter facilitating ovarian steroid hormone synthesis in vertebrates and insects [40–44], and *ebony*, which makes N-β-alanyl-dopamine from dopamine, a process associated with yellow cuticular pigmentation instead of the brown/black tan resulting from dopamine melanization [45,46]. This region therefore contains candidates for larval development, ovary development and pigmentation, all of which differ in the Cape bee compared to other subspecies of *A*. *mellifera*.

Locus D on chromosome 6 (~40kbp wide; 5 genes) is centered on *β-glucosidase*, a digestive enzyme associated with carbohydrate metabolism, including cellulose digestion. In the honeybee, the gene codes for an enzyme that hydrolyses small mono-glycosides, with a particular preference for phenyl-glycosides such as *β*-*p*NPG [47–49]. While the EHH signal decays rapidly around the *β-glucosidase* gene, neighboring SNPs around the accession also have unusually high *F*_ST_ values. To either side of the accession are the UDP glycosyltransferase (UGT) genes encoding the two proteins *UDP-glucuronosyltransferase 1-3-like* and *UDP-glucuronosyltransferase 2B13-like*. UGT enzymes catalyze reactions that metabolize oligosaccharides and conjugate sugars to a broad range of lipophilic compounds (see GO analysis results above). *β-glucosidase* has been shown to have a role in chemical signaling in social insects [50–52]. In the noctuid moth *Mamestra configurata*, *β-glucosidase* produces a glucoside precursor of the phenylethanol sex pheromone [50]. 2-phenylethanol has been suggested to advertise ovary status between young unmated honeybee queens and workers during the queen elimination phase [53], but the role of *β-glucosidase* for producing the pheromone has not yet been studied in the honeybee.

We find additional signatures of selection on loci outside of these three largest sweeps. High CSS scores across accession GB40769 “*dehydrogenase/reductase SDR family member 11-like*” (LOC412458) on chromosome 1 (locus A). SDRs make up large families of NADPH-dependent oxidoreductases with central metabolic functions [54]. The *Drosophila* orthologue is CG9360, a NADP+-dependent farnesol dehydrogenase (FOHSDR; KEGG: K15890) [55]. The orthologue detected in the closely related honeybee *Apis florea* using BLASTp [56,57] is also annotated as “farnesol dehydrogenase-like” (E-value = 1e^-140^; 88% identity; XP_003690738.1). This gene may therefore code for a farnesol dehydrogenase enzyme in bees. FOHSDR acts in the JH biosynthesis pathway and oxidizes the sesquiterpene alcohol farnesol into farnesal, a precursor of JH [39,58]. JH has an important function regulating ovary development and transition to adulthood [36].

Locus C, on chromosome 2 encompasses GB50742 (LOC102655146), annotated as “*Bardet-Biedl syndrome 1 protein-like*” (BBS1), a member of the BBSome protein complex, which is an essential protein transporter for cilium assembly and localizes near the primary cilium and centrosome in the cell [59,60], which suggests it may be a candidate for thelytoky. We detect a CSS peak in the intron of GB54634 (LOC725260; *Drosophila* orthologue: CG9896) on chromosome 7 (locus E). This gene encodes a protein that is currently uncharacterized across the animal kingdom: searches with NCBI BLASTp and in the Conserved Domain Database did not identify any functionally annotated orthologues or known domains. Locus F encompasses accession GB43519 (LOC411614) on chromosome 11, corresponding to the Neuropeptide Y-like (NPY; the insect orthologue is NPF) receptor *snpfR*. NPF is a neurohormone that regulates feeding motivation and behaviors in animals and is up-regulated in honeybee foragers compared to nurse bees [61]. We hypothesize that this gene is involved in lack of foraging in the Cape bee that occurs during social parasitism. On the same chromosome (locus G), we also find outlier SNPs across accession GB44980 (LOC409260), which codes for an “*epidermal retinol dehydrogenase 2-like*” protein. Retinol dehydrogenases oxidate retinol into retinal and are important for vitamin A metabolism and the production of photopigments and retinoic acid, essential for vision, neural development, organogenesis and reproduction across animal taxa [62–64].

Locus J is centered on GB49919 (LOC724687) on chromosome 13, which codes for a protein with a Guanylate-kinase-associated protein domain (GKAP) and shows high similarity towards insect sequences annotated as disks large-associated protein 5-like (DLGAP5). The *Drosophila* orthologue is MARS (FBgn0033845), which is the fly version of the vertebrate DLGAP5-gene (syn. HURP). In both the fly and vertebrates, this protein promotes microtubular stability in mitotic and meiotic spindles prior to cell division [65–68], making it a good candidate for involvement in thelytoky in the Cape bee. Close to the end of chromosome 16, we detect a clear selection signature across accession GB45973 (locus L). This gene codes for the Dopa decarboxylase (Ddc) enzyme, which converts L-dopa to dopamine, a neurotransmitter with diverse functions in insects, including roles in aversive learning and memory formation, innate immunity, and ovary development [69–71].

### Characterization of selective sweep regions

We next analyzed patterns of genetic variation in the 12 putative selective sweep regions (Table 2). Cape bee haplotypes with strongly selected alleles can be expected to have reduced levels of genetic diversity compared to unselected Cape and African haplotypes. In a “hard” selective sweep, a single selected haplotype rises in frequency in the population, effectively reducing diversity to zero around the selected variant. In a “soft” sweep on the other hand, the selected variant already occurs on several haplotype backgrounds when selection starts, or recombine early during the selection process, such that a mix of haplotypes carrying the putative variant rise in frequency in parallel, essentially preserving more genetic variation around the selected allele compared to a hard sweep [72].

To address whether gene candidates may typically have been undergoing “hard” or “soft” selection in the Cape bees, we defined a core SNP with the highest *F*_ST_ value for each region. We then defined a core haplotype region as being within 0.01 cM of this selected SNP and measured haplotype diversity linked to the selected and non-selected allele. In every case we observed distinctly reduced genetic variation among the core haplotypes with the selected allele (*π* was reduced by more than 50% in all cases) but there was no evidence of a single core haplotype (i.e. with no genetic variation) being linked to the core SNP in any sweep (Table 2). This indicates that selection in these regions has most likely occurred on standing variation, and that the targets of selection were initially present on more than one haplotype, representing soft sweeps rather than hard sweeps. The patterns of genetic variation are influenced by a number of factors, including the timing and strength of selection, and the diversity of haplotypes bearing the selected variant at the onset of selection. However, it is clear that the sweeps B, D and H, that contain the strongest CSS signals, all span relatively large regions of the genome (>10 kb) indicating that selection may have been more recent in these regions, whereas sweeps J and L, centered around the MARS and Ddc genes, respectively, define small regions (<6kbp) which may represent one or more older rounds of selection.

### Evaluation of the gemini locus

A single locus on chromosome 13 was previously implicated in control of thelytoky [17,18], and it was hypothesized that a 9 bp deletion (the *thelytoky associated element 1*; tae1) affecting alternative splicing of the *gemini* transcription factor within this locus governs the switch to social parasitism in the Cape bee [19]. However, a recent study did not find any association with thelytoky and the chromosome 13 locus, and found that the 9 bp deletion was polymorphic in populations of other honeybee subspecies [20]. It has also been suggested that this locus could be associated with reproductive dominance shown by Cape bee workers [18–20]. In the current honeybee genome build (OGSv3.2) the *gemini* locus maps to adjacent gene accessions, GB48238 and GB48239, which lie within a 10 kbp region on chromosome 13. The average CSS values of these accessions are both <0.3, which is below the average of all CSS values in the dataset (CSS_mean_ = 0.383). None of the SNPs in this region are present among our candidates for selection. It is therefore unlikely that a major locus for thelytoky or any other Cape bee trait lies within this region.

We also used the mapping of short reads to investigate the segregation of the 9 bp deletion in our dataset (S9 Fig). The honeybee reference genome sequence does not contain the deletion. In some of our samples, at least one read mapped across the deletion locus with an alignment gap, which is strong evidence for the presence of a deletion in at least one allele. In other samples, at least one read mapped across the deletion without such a gap, which is evidence for the presence of at least one non-deletion allele. Finally, there are some samples where no reads map across the locus, which suggests that a deletion is likely present, although considering the relatively low average read depth, such patterns could also occur by chance. We find similar proportions of these read mappings in all three African subspecies, and we therefore estimate that the frequency of the deletion is similar (30–40%) across these populations. Importantly, we find strong evidence of the presence of the non-deletion allele at high frequencies in the Cape bee samples, and the presence of the deletion allele in both *A*. *m*. *scutellata* and *A*. *m*. *adansonii*. It is therefore not possible that this deletion controls the switch to social parasitism in the Cape bee.

## Discussion

Cape bees (*A*. *m*. *capensis*) inhabit the biodiverse coastal Fynbos ecoregion of South Africa, whereas the neighboring savannah habitats of the Central Plateau are inhabited by the closely related *A*. *m*. *scutellata* (Fig 1). In its natural habitat, Cape bee worker reproduction is rare in colonies with a queen [73], but increases if the queen is lost or during reproductive swarming [12]. Nevertheless, worker reproduction contributes significantly to the Cape bee population [74]. Cape bee workers can also act as parasites, by entering colonies of *A*. *m*. *scutellata* and laying eggs, even in the presence of the queen, which is eventually lost [75]. The reproducing Cape workers do not participate in foraging [76] and the colony gradually dwindles and finally dies [77,78], but the offspring of the parasitic workers is able to disperse and parasitize other nests. This dramatic invasive behavior has been coined social parasitism. A number of traits observed in Cape bees facilitate this behavior, including development of worker ovaries, reproductive dominance and reproduction by thelytokous parthenogenesis. The reason why these traits appear in the Cape bee is unclear, but it is possible that they have an adaptive advantage in the Fynbos area where they predominantly occur (reviewed in [5]). In addition to biological interest, the social parasitism can have disastrous impacts on apiculture as shown during the “capensis calamity” in the early 1990s when cape bees were introduced outside of native range and had severe negative effects on the native colonies of *A*. *m*. *scutellata* [78–80].

We hypothesized that the traits involved in social parasitism in the Cape bee represent derived adaptations that have been produced by positive selection in the Cape bee population. We analyzed genetic variants with high *F*_ST_ in the Cape bee population compared to a background population consisting of two other African subspecies. We find that SNPs with high *F*_ST_ in this comparison are strongly associated with signals of selection in the Cape bee population inferred by the PBS and XP-EHH tests, rather than being associated with selection in the background population. In the reciprocal comparisons analysing highly differentiated SNPs in the two other African subspecies, similar strong associations with selection signals were not observed. This indicates that positive selection had been more prevalent in the Cape bee population, and is therefore likely to be associated with the unique set of derived traits.

To identify genetic variants that control these traits we performed a genome scan for loci under selection using the CSS statistic that combines *F*_ST_ and XP-EHH. We find clear signals implicating multiple loci in the suite of traits specific to the Cape bee. Selection signals are found in several genomic regions and encompass a diverse variety of genes, consistent with the diverse set of traits connected to social parasitism. Notably however, we find no evidence for a connection between a putative thelytoky locus on chromosome 13 and social parasitism in the Cape bee [18], as there are no SNPs in this region with high *F*_ST_ or CSS in our dataset. In addition, we find that a 9 bp deletion, previously suggested to be associated with thelytoky in the Cape bee [19], likely segregates at similar frequencies in other African populations, as also found in a recent study [20]. It is therefore highly unlikely that genetic variation in this region, in particular the 9 bp deletion, has any connection to social parasitism in Cape bees.

Our results suggest that multiple loci, rather than a single master regulator, are responsible for the switch to social parasitism in Cape bees. The candidate loci presented here can be used in further experiments to elucidate the biological basis of these traits. Some previous studies have used F1 hybrid queens from Cape bees and another subspecies backcrossed to Cape bee drones to analyze the segregation of the putative thelytoky locus [18–20]. Laying workers produced from such crosses exhibited a ratio of thelytoky:arrhenotoky close to 1:1 predicted if thelytoky was controlled by a single locus. However, it is important to note that while this is consistent with control by a single locus, it could still be observed if the hybrid was heterozygous at multiple loci that control thelytoky. In addition, there are a number of additional traits exhibited by the Cape bee that facilitate worker reproduction and social parasitism but are not functionally connected to cell division, which are likely to be controlled by different loci. It has previously been found that ovary activation and pheromone excretion only co-vary in Cape bees but are not fully associated (some individuals express only one trait), suggesting that they are controlled by more than one locus [81].

Genetic markers that co-vary with the Cape bee phenotypes and distinguish Cape bees from the *scutellata* population have previously been lacking or found to be inconsistent [3]. The highly differentiated SNPs identified here also have the practical benefit of being able to unambiguously identify the degree of introgression from Cape bees using only a few SNP markers. For example, there are two regions, on chromosomes 1 and 11, respectively, with fixed variants that separate all Cape bees in our samples from all other African bees in our sample. Typing these two genetic markers would unambiguously distinguish Cape bees from neighboring populations and allow confident assessment of gene flow involving Cape bees.

The Cape bees from our two sample locations have different degrees of differentiation compared to the other African bees: there are seven times as many fixed variants between the western Cape Town sample and other African bees as there are between the eastern Port Elizabeth sample and other African bees in our dataset (423 variants across 10 chromosomes vs 60 variants across 2 chromosomes, respectively) and the haplotypes on which these fixations occur are more divergent from other African bees in the Cape Town sample. Stronger selection pressure or higher degrees of reproductive isolation could be driving these differences. The western Fynbos region can be considered at the current center of the Cape bee genotype, whereas the eastern subpopulation, located closer to the transitional zone, may have been subject to more introgression from *scutellata*-type genotypes. This is supported by a western-to-eastern cline observed in phenotypic variation [3]: worker bees from the western region have larger ovaries with up 50% more ovarioles than do the eastern bees [22,82], they produce diploid female offspring through thelytoky at higher ratios [83] and although they do not mate, they have more developed spermathecae compared to the eastern bees [82], rendering them more queen-like.

Cape bee workers can lay diploid unfertilized eggs through thelytokous parthenogenesis. At the cytological level, the diploid nucleus of the egg is produced after the second meiosis. Following an atypical linear orientation of the two meiotic spindles, four nuclei are produced and the central pronucleus fuses with the central descendant of the first polar body, migrates into the egg and initiates normal embryological cleavage as if the egg had been fertilized by a sperm [6]. Among our outlier SNPs are non-synonymous variants in three candidate genes, which code for proteins that could potentially play roles in chromosomal segregation and thelytoky in the Cape bees. These genes modulate the interaction between the centrosome (the microtubule organizing center of the cell) and the meiotic spindle (the structure responsible for separating chromatids during cell division). The gene GB45239 at locus H codes for an uncharacterized protein that has peptide sequence similarity towards an SMC domain. SMC proteins bind chromosomes and are essential for organizing the segregation of sister chromatids during cell division [84,85]. The gene GB50742 in locus C codes for a BBS1-like protein. BBS proteins are typically associated with the assembly of the primary cilium. Mutations in BBS genes are implied in many ciliopathic disorders and have been shown to disrupt the establishment of planar cell polarity [86,87]. Some BBS proteins, including BBS1, are also localized to the centrosome and their knockouts and mutants adversely affect spindle microtubule and pole function, resulting in misaligned chromosomes during mitosis in mice [88]. The gene GB49919 (locus J) gene codes for a protein with a GKAP domain and the *Drosophila* orthologue MARS has been shown to be required for successful centrosomal binding and the maintenance of spindle bipolarity during chromosome segregation [65–68]. In *Drosophila*, a single point mutation in the structurally similar guanylate kinase enzyme has been demonstrated to convert it into a spindle orientation protein [89], suggesting that the spindle apparatus is sensitive even to small genetic changes.

Cape bee worker development is unusually fast, with workers emerging about a day earlier than workers of European and other African subspecies [90,91]. Cape bee workers also have well-developed and readily activated ovaries [3]. Morphogenesis and ovary development and activation in honeybees are associated with changes in juvenile hormone (JH) and ecdysteroid levels [34–36]. Ecdysteroids are also produced in ovaries, deposited in eggs and regulate embryogenesis [42], including gonadal development in the larva [92]. In queen-destined honeybee larvae, increased levels of JH promote ovary development by protecting ovarian tissues from apoptosis [58,93]. We detect signals of selection across several loci that may modulate levels of these hormones, including a cluster of glucose-methanol-choline (GMC) oxidoreductases located on chromosome 1. In particular the strongest signal in our dataset is found in the ecdysis triggering hormone receptor (Ethr). Ecdysis triggering hormone is essential for initiating ecdysis among insects, and directly regulates JH synthesis in mosquitos [38,39]. The SDR11-like locus (locus A) shows high peptide similarity towards farnesol dehydrogenase (FOHSDR) in other bees and insects, which is responsible for converting farnesol into farnesal, an early step in the pathway for JH biosynthesis. Worker ovary size and JH signaling both correlate with worker behavior [94]. We hypothesize that these loci modulate larval development, ovary development, and/or worker behavior through their effects on JH and ecdysteroid levels.

Our scan identifies a locus coding for a 4-coumarate-CoA ligase. 4-coumaric acid is present in jelly fed to worker-destined larvae and triggers a genetic cascade resulting in worker caste determination and reduced ovary development [95]. It is therefore possible that variants in this gene in the Cape bee may promote ovary activation in workers. Among the top signals is a sweep localized to the gene coding for dopamine decarboxylase (Ddc) that include several changes to the peptide sequence. Dopamine levels are associated with both memory formation [96], behavior and ovary development in workers and is normally controlled by queen pheromones [97]. Royal jelly fed to queen-destined larvae is rich in dopamine [98]. When fed to workers, royal jelly stimulates production of dopamine and other neurohormones in the brain, induces ovary activation and reduces the willingness of workers to participate in foraging [99]. Under queen-less conditions, dopamine levels as well as gene expression for biogenic amine receptors are upregulated in workers and correlated with ovary activation and reproductive status [71,100,101]. This locus has thus been annotated for functions and effects that closely mirror the Cape bee phenotype.

Our analyses for selection therefore detects candidates that can be linked to three important aspects of the Cape bee phenotype: i) loci involved in centrosome function may affect chromosomal segregation and be responsible for Cape bee thelytoky; ii) loci involved in ecdysteroid, JH and dopamine signaling may promote worker ovary development; iii) loci involved in worker behavior including reproductive dominance and foraging. Given the number and nature of selection signals and candidate loci that are revealed in our comparative genome scan, we reject a model that explain all Cape bee phenotypes as being controlled by a single master-switch locus, and instead propose that a mix of multiple genotypes affect different phenotypes, which may have evolved independently.

## Materials and Methods

### Sampling

Genome variation in the *A*. *m*. *capensis* (Cape bee) population was inferred by sequencing diploid worker bees sampled from different colonies at two locations in the coastal Fynbos ecoregion of South Africa: five bees each were sampled from the western (Stellenbosch, Cape Town) and eastern parts (Kragga Kamma Game reserve region; Port Elizabeth; Fig 1), respectively, for a total chromosome sample size of 20. In both of these locations, laying workers produce nearly exclusively female offspring. The documented ratio of male:female offspring is 0:1 in these locations [22]. Additional worker bees sampled from two sub-Saharan honeybee subspecies without the social parasite phenotype were taken as representatives of a unselected African background population (n = 40): ten *A*. *m*. *scutellata* worker bees were collected from apiaries in the Pretoria region and ten *A*. *m*. *adansonii* worker bees were sampled from apiaries in Kaduna state, Nigeria. In addition, 28 European worker bees collected in Spain (*A*. *m*. *iberiensis*; n = 9) and Scandinavia (*A*. *m*. *mellifera*; n = 19) were used as a distantly related European outgroup. Short reads were mapped against version 4.5 (Amel_4.5) of the honeybee genome [102] and the African SNP dataset included 6.2 million phased genotypes. The samples were originally sequenced as part of a global survey of genetic variation. Refer to Wallberg *et al*. [21] for additional information about collection sites and the mapping and genotyping pipelines.

### Scans for selection on Cape bees

Cape bee workers rarely produce haploid males but frequently produce females [25]. Although the ability to produce female offspring by thelytokous parthenogenesis and act as a social parasite is a trait that appears to be fixed (or close to fixation) in Cape bee populations, most reproduction in such populations is still due to sexual reproduction by queens. The exception to this is when the queen is lost or during reproductive swarming, and a new sexually reproducing queen may be produced by an unfertilized worker-laid egg. We therefore expect that patterns of variation and linkage disequilibrium in Cape bees should be similar to populations of other subspecies, which is observed. Overall levels of genetic variation are similar between Cape bees and other African subspecies [21], suggesting that the presence of asexual reproduction does not markedly reduce the effective population size in the Cape population. Episodes of positive selection should therefore leave characteristic patterns of selective sweeps in restricted genomic regions typical of an outcrossing, sexually reproducing species.

We used a combination of allele frequency and haplotype structure analyses of the Cape bee genome to identify candidate variants and genes that occur in genomic regions with signatures of selection. Allele frequency differences between the Cape bees and the African background population (*scutellata* + *adansonii*) were calculated to estimate the per-SNP (n = 6,245,176) fixation index *F*_ST_ using the Weir-Cockerham equation [103] and identify fixed or nearly-fixed variants between the two populations. Genetic divergence between the Cape bees and the African reference populations were inferred across the full genome and over non-overlapping 1kbp and 100kbp windows using the *F*_ST_ estimator of Reynolds *et al*. [104]. The window-based *F*_ST_ estimates were then transformed into divergence times *T* and used to estimate the Population Branch Statistic (PBS), according to the procedure described by Yi *et al*. [27]. By comparing the divergence times between the two African populations to the corresponding divergence times between each population and the European outgroup, the PBS makes it possible to identify the divergent regions in which genetic changes are associated specifically with drift or selection in the Cape bees. The PBS therefore was computed from three pairwise *F*_*ST*_ comparisons: i) Cape bees vs the African background population; ii) Cape bees vs the European outgroup; and iii) the African background population vs the European outgroup. PBS>0 identifies genomic regions in which the Cape bees diverge from both the SA population and the European outgroup, ie uniquely derived alleles in the Cape bees.

To detect changes in haplotype diversity around candidate loci in the Cape bees, we applied the cross-population extended haplotype homozygosity (XP-EHH) test as implemented in the program *selscan* [28] for every SNP with a minor allele frequency (MAF) > = 0.02. While strong selection is expected to generate long haplotypes of linked variants, recombination should counter-act to disassociate them and degrade the haplotypes over time. A recombination map previously inferred from the African honeybee genotypes [105] was used to compute the average recombination rates over windows of 100kbp. These rates were next used to specify genetic distances between the SNPs and incorporated into the EHH analyses. Honeybee recombination rates are extremely high, on average ~25cM/Mb, making them the highest rates recorded in any animal species [105–107]. We therefore expect haplotype homozygosity patterns to decay relatively quickly around selected loci, allowing for high precision in the haplotype scan. The decay of EHH in the Cape bees were queried against the corresponding patterns observed in the African background population at each SNP and the output was normalized. The program traces the decay of haplotype homozygosity around every putative “core” SNP. The average SNP density in the dataset is ~1 SNP per 30bp and estimates for core SNPs near long gaps to the next variable position (>10,000bp) that had not yet decayed below the EHH cutoff threshold (0.05) were discarded, to avoid inference in regions with unusually little data or uncertainty about the physical distance between markers. Using this filter, XP-EHH estimates for 0.35% of SNPs discarded, the vast majority of which were located close to scaffold borders. In addition to per-SNP XP-EHH scores, we computed the average score across the same windows for which we had previously estimated divergence and the PBS.

In our framework, the most promising candidate loci are those that contain *F*_ST_ outlier SNPs associated with unusually high PBS and XP-EHH scores. In order to identify the SNPs and genes with the most comprehensive evidence of selection, we computed a single unbiased Composite Selection Score (CSS) by combining *F*_ST_ and XP-EHH estimates, following the procedure proposed by Randhawa *et al*. [24]. To compute the CSS, all SNPs are ranked for each statistic. For each SNP, the fractional rank positions for *F*_ST_ and XP-EHH, respectively, are converted into two z-statistics, the mean of which corresponds to a single joint rank for both statistics. The corresponding *p*-value is retrieved from a standard normal distribution. The CSS score is then taken as–log_10_*p*. We also computed CSS scores across 1kbp and 100kbp non-overlapping windows, by taking the Reynolds *et al*. [104] *F*_ST_ estimator and the average XP-EHH estimated across each window. The windows were ranked and assigned a single CSS score using the same approach as for the SNP dataset. For full-gene estimates, we then took the mean CSS of the 1kbp windows overlapping each gene body, including 2kbp of neighboring sequence to either side of the start/stop position.

### Analysis of gene candidates

SNPs were associated with genes using coordinates provided in the latest official gene set (OGSv3.2) [102] and the NCBI Annotation Release 102 (AR102), spanning ~13,000 accessions. A restricted set of candidate SNPs and genes were then evaluated for significantly enriched gene ontology (GO) terms that could indicate selection on specific biological pathways and functions. The mapped scaffolds of the honeybee genome are about 200**×**10^6^ bp and the mean density of genes is roughly one accession per 15kbp. The mean physical gene length is about 8kbp, as is the intergenic regions in between genes on the same scaffolds. Intergenic SNPs were annotated and included in the GO and downstream gene candidate analyses only if they were within 8kbp of the closest gene (43% of all intergenic SNPs). We analyzed the *Drosophila melanogaster* orthologues of these gene sets using the GOrilla web platform [30].

To address whether gene candidates may have been subject to “hard” or “soft” selection in the Cape bees, we assessed the levels of genetic diversity across the twelve selective sweeps identified in our scan. These sweeps were associated with 25 gene candidates with comprehensive selection signals. For each sweep, a single core SNP was chosen from the outlier SNPs in the sweep. The core SNP was taken as the SNP with the highest *F*_ST_ value in the sweep region. In the case of a tie between more than one top *F*_ST_ SNP, the SNP with the highest XP-EHH value among the competing SNPs was designated to be the core variant. Cape bee haplotypes with the high frequency variant at the SNP were classified as putatively selected haplotypes, whereas Cape bee haplotypes without the variant (if any) were put together with *adansonii* and *scutellata* haplotypes as unselected haplotypes.

We estimated genetic diversity across the region by computing the average pairwise differences between all haplotypes within each of the “selected” and “unselected” groups using the *π* statistic. *π* was computed for non-overlapping windows of 0.01cM of genetic length for up to 20cM away from either side of the core SNP (2000 windows to either side of the core). *π* was also computed for non-overlapping windows of 1kbp physical length for up to 2Mb away to either side of the core SNP. For every window, the relative difference in genetic diversity between the selected and unselected haplotypes was computed as:

$$\Delta \pi =\frac{\left(\pi_{selected}-\pi_{unselected}\right)}{\pi_{unselected}}$$

We traced diversity in windows upstream and downstream of the core SNP. In each case, the center (or first) core window contained the core variant itself. At every distance interval away from the SNP, the average *π* was computed from the corresponding upstream and downstream window, effectively folding the two-sided sweep profile across the variant into a one-sided sweep profile. A genomic average background Δ*π* between the two groups, Δ*π*_B_, was computed from averaging Δ*π* across the 1000 most distant windows around all twelve sweeps. The two analyses trace diversity up until different maximum distances away from the core, but Δ*π*_B_ was found to be close 0 in both analyses (Δ*π*_B_ = -2% across the 1kbp windows; Δ*π*_B_ = 4% across the 0.01cM windows). Δ*π* was negative for all core windows containing the core variants but approached or passed Δ*π*_B_ between the two groups within the first 30 windows regardless of using genetic or physical distance as units of distance. The length of a sweep was taken as the first distance interval at which the average Δ*π* was equal to or higher than Δ*π*_B_.

### Genetic basis of thelytoky

In the Cape bee, the genetic basis of thelytoky was investigated previously using backcrosses of hybrids formed by crossing *A*. *m*. *capensis* with *A*. *m*. *carnica* [17]. Out of four laying workers produced by the *A*. *m*. *carnica* backcross, none were thelytokous, whereas out of 31 laying workers produced by the *A*. *m*. *capensis* backcross, the proportion of thelytoky to arrhenotoky was not significantly different from 1:1. This pattern is consistent with inheritance of thelytoky being determined by a single recessive locus, termed *thelytoky (th)* by the authors of the study. However, it should be noted that such a pattern could also be produced if multiple loci were involved.

A similar backcross scheme to produce workers that were hypothetically homozygous or heterozygous for the putative *th* locus was used for genetic mapping using a panel of microsatellites. A significant association with the mode of reproduction was found on chromosome 13, which was inferred to be the location of the *th* locus [18]. The amount of queen substance was found to be higher in thelytokous workers produced by this backcross, and the onset of egg-laying to be earlier, leading the authors to suggest that *th* also controls these traits, which are related to reproductive dominance. A subsequent study used RNAi to knock out an exon of the candidate gene *gemini* within this region, and showed that it resulted in worker ovary activation, implicating it as the *th* locus [19]. It was proposed that a 9 bp deletion in the intron of the *gemini* gene regulates alternative splicing, thus generating thelytoky and its associated traits.

More recently, however, Chapman *et al*. [20] were unable to replicate these results. Using reciprocal backcrosses of *A*. *m*. *capensis* and the closely related subspecies *A*. *m*. *scutellata*, they found no evidence of an association between observations of thelytoky and a genetic marker linked to the *th* locus. They also found that the 9 bp deletion was common in other populations of *A*. *mellifera* without any reported thelytoky. This observation indicates that it is unlikely that the 9 bp deletion determines thelytoky in Cape bees. The genetic control thelytoky in the Cape bee is therefore far from clear from these studies. For instance, although backcrosses produce patterns of segregation consistent with a single locus controlling thelytoky, it is possible that more loci are involved in this trait.

## Supporting Information

S1 Fig Low-*F*_ST_ SNPs dominate the dataset and the western subpopulation shows more fixation against other populations. (A) *F*_ST_ was computed for every SNP segregating between the Cape bee (*capensis*, n = 10) and the *scutellata + adansonii* (SA; n = 20) background population (n = 6,245,176) and counted for *F*_ST_ bins of 0.05. The distribution is dominated by variants segregating at similar frequencies between the two groups: 93.3% of SNPs (n = 5.89 x 10^6^) have *F*_ST_ values below 0.1 (blue area) whereas 0.33% of SNPs (n = 20,460) have *F*_ST_ values above 0.3 (green area). 45 SNPs in the dataset are fixed between the two groups. (B) *F*_ST_ was computed for every SNP between the western Cape Town subpopulation (CT; n = 5) and SA (n = 20) (n = 5,934,995; black line) and the eastern Port Elizabeth subpopulation (PE; n = 5) and SA (n = 5,907,010; grey line) individually, and binned as in (A). The PE vs SA comparison has significant overrepresentation of low *F*_ST_ SNPs (*F*_ST_ = 0.00–0.005; 4,880,183 vs 4,810,196; p<1e^-5^, chi-squared test), whereas the CT vs SA comparison have significantly more SNPs segregating at every bin with *F*_ST_>0.05 (p<1e^-5^, chi-squared test). (C) The individual counts from (B) were combined and the proportion of CT vs SA variants segregating at each *F*_ST_ interval was computed. For low *F*_ST_ variants (*F*_ST_<0.3), CT SNPs make up less than 60% of SNPs (proportion CT SNPs per bin ranges from 0.496 to 0.549), whereas they are increasingly overrepresented for higher *F*_ST_ variants (*F*_ST_>0.5; proportion CT SNPs per bin 0.694–0.856).
(PDF)

S2 Fig Extreme haplotype homozygosity and divergence is more pronounced in Cape bees than the background population and associate with high-*F*_ST_ SNPs. (A) The XP-EHH was estimated for SNPs with MAF>0.02 (n = 6,196,550) using the program *selscan* ([28]) and binned (units of 0.01; blue distribution). The average XP-EHH for was also computed for 1kbp windows and binned (n = 188,088; units of 0.01; red distribution). The distribution is centered around 0 (mean XP-EHH = 0.007). The upper and lower 99.9% and 99.5% percentiles were identified from the empirical distribution of SNP and window XP-EHH estimates. These are more extreme when associated with the Cape bees (XP-EHH>0), compared to the background population (*scutellata + adansonii*; XP-EHH<0). 99.9% percentiles for SNPs (blue solid lines): 4.12 vs -3.46. 99.5% percentiles for SNPs (blue dashed lines): 2.9 vs -2.74. 99.9% percentiles for windows (red solid lines): 4.19 vs -2.72. 99.5% percentiles for SNPs (blue dashed lines): 2.53 vs -2.06. (B) The population branch statistic (PBS) was estimated for 1kbp windows across the genome (n = 189,053). The distribution is slightly skewed towards higher PBS in the Cape bees than the background population (mean PBS = 0.039). Upper and lower 99.9% and 99.5% percentiles were identified from the empirical distribution of PBS estimates. These are more extreme when associated with the Cape bees (PBS>0), compared to the background population (PBS<0). 99.9% percentiles (green solid lines): 1.27 vs -0.34. 99.5% percentiles (blue dotted lines): 0.51 vs -0.16. (C) The XP-EHH of SNPs was cross-referenced with the *F*_ST_ computed from the allele frequency differences between the Cape bees and the *scutellata + adansonii* background population. SNPs were binned for *F*_ST_ (units of 0.05). For every *F*_ST_ class, the mean XP-EHH was computed and 95% confidence intervals were computed from 200 bootstrap replicates. Each class was compared to the upper percentiles retrieved from the empirical XP-EHH distribution compiled in (A), which are associated with long haplotypes in the Cape bees. The 99.9% percentile is 4.12 (grey solid line); the 99.5% percentile is 2.9 (grey dashed line); and the 99% percentile is 2.49 (grey dotted line). SNPs with *F*_ST_>0.85 have haplotype homozygosity patterns that are strongly biased towards long haplotypes in the Cape bees and have XP-EHH values that exceed, on average, those of the top 0.5% of SNPs (p<0.01). (D) Cross-reference between the *F*_ST_ of SNPs and the average XP-EHH for the 1kbp window in which the SNP was located. *F*_ST_ classes and bootstraps used as in (C) and empirical percentiles from (A). The 99.9% percentile is 4.19 (grey solid line); the 99.5% percentile is 2.53 (grey dashed line); and the 99% percentile is 2 (grey dotted line). The result is similar to (C). SNPs with *F*_ST_>0.85 occur in regions with long haplotypes in the Cape bees, exceeding, on average, those of the top 0.5% regions across the genome (p<0.01). (E) Cross-reference between the *F*_ST_ of SNPs and the PBS for the 1kbp window in which the SNP was located. *F*_ST_ classes and bootstraps used as in (C) and empirical percentiles from (B). The 99.9% percentile is 1.27 (grey solid line); the 99.5% percentile is 0.51 (grey dashed line); and the 99% percentile is 0.34 (grey dotted line). SNPs with *F*_ST_>0.7 occur in regions with divergence patterns that are biased towards a long branch leading to the Cape bees, exceeding, on average, the divergence detected in the top 0.5% regions across the genome (p<0.01).
(PDF)

S3 Fig Extreme haplotype divergence and homozygosity signals centers around outlier SNPs (*F*_ST_>0.9) and decay after 40–60kbp. (A) Every SNP with *F*_ST_>0.2 and minimal evidence for extended haplotype homozygosity (XP-EHH) in the Cape bees (XP-EHH>0) was put in *F*_ST_ bins of 0.10. The linked divergence was traced around each SNP by computing *F*_ST_ across 1kbp windows for up to 500kbp to either side of each SNP. 95% confidence intervals were computed from 200 bootstrap replicates for every SNP *F*_ST_ class and distance. High *F*_ST_ SNPs appear to be clustered together in peaks: 3kbp away from the most highly differentiated SNPs (*F*_ST_>0.9), the window-based divergence is higher (0.6) than for SNPs with *F*_ST_ = 0.7–0.8 (0.3; p<0.01), indicative of higher density of highly differentiated SNPs around the former. The decay of linked signals was compared against genome-wide percentiles: top 0.1% (solid line), top 0.5% (dashed line), 1% (dotted line). Linked divergence signals extend longest around high *F*_ST_ SNPs: divergence drops to the top 1% level after about 50kbp for SNPs at *F*_ST_>0.9, compared to about 20kbp for SNPs at *F*_ST_ = 0.8–0.9 and 4kbp for SNPs at 0.7–0.8. The most differentiated SNPs therefore appear to be located in regions with the highest and widest linked divergence. (B) Tracing the decay of the population branch statistic (PBS) using the same procedure and SNPs as in (A). SNPs with high *F*_ST_ are clustered for the PBS and correlated with the widest linked signals: 3kbp away from SNPs with *F*_ST_>0.9, the PBS in linked regions is significantly elevated compared to other SNPs with *F*_ST_ = 0.7–0.8 (1.1 vs 0.45; p<0.01) and it takes about 60kbp for the PBS to drop to the top 1% level around *F*_ST_>0.9 SNPs, compared to 40kbp for SNPs at *F*_ST_ = 0.8–0.9 and 15kbp for SNPs at 0.7–0.8. The most differentiated SNPs are hence located in regions that with a long branch against both other African and European bees. (C) Tracing the decay of the XP-EHH statistic using the same procedure and SNPs as in (A). XP-EHH was estimated for SNPs with MAF>0.02. The average XP-EHH was computed for every 1kbp window up to 500kbp away from each SNP. The general pattern is consistent with the estimates for divergence. SNPs with high *F*_ST_ typically occur in regions with the widest haplotype homozygosity signals: 3kbp away from SNPs with *F*_ST_>0.9, the XP-EHH in linked regions is significantly elevated compared to SNPs with *F*_ST_ = 0.7–0.8 (4 vs 1.8; p<0.01). It takes about 40kbp for the XP-EHH to drop to the top 1% level around *F*_ST_>0.9 SNPs, compared to 20kbp for SNPs at *F*_ST_ = 0.8–0.9 and only 2kbp for SNPs at 0.7–0.8.
(PDF)

S4 Fig The Cape bees have a larger number of divergent regions across the genome than the other two subspecies. The fixation index (*F*_ST_) was computed for every SNP segregating between each subspecies and a combined population consisting of the other two African subspecies (further described in Fig 2). (A) *A*. *m*. *capensis* vs *adansonii* + *scutellata*. The main Cape bee scan has *F*_ST_ peaks (*F*_ST_>0.8) distributed across 13 chromosomes. (B) *A*. *m*. *scutellata* vs *adansonii* + *capensis*. The *scutellata* scan detects a single *F*_ST_ peak (*F*_ST_>0.8) on chromosome 1. (C) *A*. *m*. *adansonii* vs *capensis* + *scutellata*. The *adansonii* scan detects *F*_ST_ peaks (*F*_ST_>0.8) distributed across 6 chromosomes. Most of these SNPs are clustered on chromosomes 7 and 11.
(PDF)

S5 Fig The western subpopulation is associated with more extensive selection signals than the eastern subpopulation. (A) SNPs segregating between the Cape bees and the African background population (*scutellata + adansonii*) were sorted for *F*_ST_ and the top 0.1% (n = 6245) were filtered for minimal evidence for extended haplotype homozygosity in the Cape bees (XP-EHH>0) to make a candidate set of variants with trending evidence for selection in the Cape population (n = 2917). The decay of the population branch statistic (PBS) and XP-EHH, respectively, was traced in 1kbp windows for the western Cape town (CT; black line) and eastern Port Elizabeth (PE; grey line) subpopulations for up to 500kbp to either side of every such SNP. 95% confidence intervals (grey) were computed from 200 bootstrap replicates at every distance. The mean PBS and XP-EHH signals are distinctly stronger in CT compared to PE close to these SNPs: at 3kbp away from a SNP, PBS_CT_ is almost twice as high as PBS_PE_ (0.70 vs 0.38; p<0.01) and XP-EHH_CT_ is almost 2.5x times higher than for XP-EHH_PE_ (1.80 vs 0.72; p<0.01). The linked signatures are significantly stronger in CT than in PE around these SNPs for hundreds of kilobasepairs: PBS_CT_>PBS_PE_ for over 500kbp (p<0.05) and XP-EHH_CT_>XP-EHH_PE_ for 350kbp (p<0.05). These haplotype patterns are consistent with stronger signatures of selection in the CT subpopulation. (B) Analysis as in (A) but tracing the PBS and XP-EHH for CT and PE around outliers (n = 3820) identified specifically between the CT subpopulation and the SA population. The pattern is consistent with (A) but the difference between the CT and PE subpopulation for linked signatures of selection is further amplified when focused around these outlier SNPs: at 3kbp away from a SNP, the mean PBS for CT is 0.66 vs 0.27 for PE (p<0.01) and the mean XP-EHH for CT is 1.76 vs 0.28 for PE (p<0.01). These haplotype patterns indicate that some signatures of selection in the CT subpopulation are considerably weaker in the PE subpopulation. (C) Analysis as in (A) but tracing the PBS and XP-EHH for CT and PE around outliers (n = 1684) identified specifically between the PE subpopulation and the SA population. Also in this comparison and set of outliers, the CT subpopulation have significantly stronger signatures of selection with regards to the PBS and XP-EHH statistics: at 3kbp away from a SNP, the mean PBS for CT is 0.62 vs 0.48 for PE (p<0.01) and the mean XP-EHH for CT is 1.36 vs 1.13 for PE (p<0.01). These haplotype patterns indicate that the signatures of selection in the PE subpopulation may have substantial overlap with or be a subset of those detected in the CT subpopulation. (D) Analysis as in (A) but tracing the PBS and XP-EHH for CT and PE around 3000 randomly sampled SNPs segregating between the Cape bees and the SA population. PBS and XP-EHH decay patterns around these SNPs are mostly absent and indistinguishable between the CT and PE subpopulations. The PBS is significantly biased towards slightly higher values in the CT subpopulation: at 3kbp away from a SNP, the mean PBS for CT is 0.083 vs 0.069 for PE (p<0.01). For XP-EHH, the 95% confidence intervals estimated for the CT and PE subpopulations overlap throughout the full range.
(PDF)

S6 Fig Genes with putatively selected variants have elevated selection signals compared to the genomic background. (A) Genetic distance (*F*_ST_ estimator of Reynolds *et al*. [104]), the population branch statistic (PBS) and cross population extended haplotype heterozygosity (XP-EHH) between the Cape bees and the African background population were estimated for 1kbp windows (*scutellata* + *adansonii*) across the full genome and joined into a Composite Selection Score (CSS; [24]). The window-based CSS estimates were cross-referenced with accessions by taking the full gene-body coordinates adjusted to include 2kbp of upstream and downstream sequence. Across all 13,281 accessions, the mean CSS score is 0.383. The 99% percentile for CSS is 1.546 and the 99.5% percentile for CSS is 1.912. The 97 accessions identified by taking the top 1000 SNPs ranked for their CSS (*F*_ST_ and XP-EHH) have significantly elevated CSS across the full gene body (CSS = 1.654; p<0.05). This set was reduced to include only accessions with SNPs above a threshold level of fixation. As these thresholds became increasingly strict, we enriched for accessions with high overall CSS scores. The 25 accessions that include putative causative variants with *F*_ST_>0.8 (99.99% percentile for *F*_ST_) and have a mean CSS score of 2.175, significantly higher than the top 1% of genes (p<0.05). 95% confidence intervals were generated from 200 bootstrap replicates of each class of genes.
(PDF)

S7 Fig Significantly enriched gene ontology (GO) terms among *Drosophila* orthologues of the Cape bee candidates. The top 1000 SNPs sorted for their Composite Selection Signal (CSS; *F*_ST_+XP-EHH) located within 8kbp from the closest gene body were associated with 97 accessions in the honeybee genome (S3 Table), 73 of which had previously been matched to 68 unique *Drosophila* accessions using BLASTx with an e-value <0.5 (Wallberg *et al*. [21]). 61 of these accessions and a background set of 6253 honeybee-fly orthologues were recognized by the GOrilla platform and the candidate set was queried for significantly enriched GO-terms. (A) We detected 13 significantly enriched biological processes (p<0.05), 10 of which had a q-value <0.05 after correcting for multiple testing. The significantly enriched terms with no nested GO-terms below them are “Ecdysteroid metabolic process” (GO:0045455) and “Acyl-coA biosynthetic process” (GO:0071616). On the left: graph showing the interrelationships among GO-terms. On the right: table of GO-terms and their associated values and counts. Enrichment abbreviations are as follows: N = the total number of genes; B = the total number of genes associated with a specific GO term; n = the number of genes in in the target set; b = the number of genes in the intersection. Enrichment is taken as (b/n) / (B/N). (B) We detected 8 significantly enriched molecular functions (p<0.05), 5 of which had a q-value <0.05 after correcting for multiple testing. The significantly enriched terms with no nested GO-terms below them are “Flavin adenine dinucleotide binding” (GO:0050660), “Transmembrane signaling receptor activity” (GO:0004888), “UDP-glycosyltransferase activity”, (GO:0008194) and “Choline dehydrogenase activity” (GO:0008812).
(PDF)

S8 Fig Twelve selective sweeps with high-*F*_ST_ variants detected in the Cape bees. Among the 97 candidate accessions associated with the top 1000 SNPs ranked for the composite selection score (CSS) were 25 accessions that include at least one SNP above the 99.99% *F*_ST_ percentile (*F*_ST_>0.80). These are located in 12 selective sweeps (labeled from A to L) across the genome. (A) Sweep A; around accession GB40769 (LOC412458; Dehydrogenase/reductase SDR family member 11-like). Top plot: *F*_ST_ of all SNPs in the region (grey dots); *F*_ST_ of non-synonymous SNPs (red dots), if detected; blue line is the population branch statistic PBS measured over 1kbp non-overlapping windows; gene bodies in grey above graph; the main accession is highlighted in green. Middle plot: XP-EHH of all SNPs in the region (grey dots); XP-EHH of non-synonymous SNPs (red dots); blue line is the mean XP-EHH measured over 1kbp non-overlapping windows. Genes as in the top plot. Bottom plot: the Composite Selection Score (CSS; from [24]) based on both *F*_ST_ and XP-EHH of all SNPs in the region (grey dots); CSS of non-synonymous SNPs (red dots); blue line is the mean CSS measured over 1kbp non-overlapping windows. Genes as in the top plot. (B) Sweep B; around accession GB46500 (Ethr; Ecdysis triggering hormone receptor). Plots as in (A). (C) Sweep C; around accession GB50742 (LOC102655146; Bardet-biedl syndrome 1 protein-like). Plots as in (A). (D) Sweep D; around accession GB54486 (LOC411978; *β*-glucosidase). Plots as in (A). (E) Sweep E; around accession GB54634 (LOC725260; Uncharacterized LOC725260). Plots as in (A). (F) Sweep F; around accession GB43519 (LOC411614; Neuropeptide y receptor-like). Plots as in (A). (G) Sweep G; around accession GB44980 (LOC409260; Epidermal retinol dehydrogenase 2-like). Plots as in (A). (H) Sweep H; around accession GB45239 (LOC100576557; Uncharacterized protein LOC100576557). Plots as in (A). (I) Sweep I; around accession GB40077 (LOC726040; Probable 4-coumarate—coA ligase 3-like). Plots as in (A). (J) Sweep J; around accession GB49919 (LOC724687; Uncharacterized LOC724687). Plots as in (A). (K) Sweep K; around accession 14 GB52757 (LOC408473; Uncharacterized LOC408473). Plots as in (A). (L) Sweep L; around accession GB45973 (Ddc; Dopa decarboxylase). Plots as in (A).
(PDF)

S9 Fig The *gemini* deletion is not fixed in the Cape bees and is detected in other African subspecies. Short read data was mapped across the putative 9bp deletion (the *thelytoky associated element 1*; tae1) in one of the introns of the transcription factor *Gemini* and visualized using samtools console output. (A) Read data for 10 *capensis* samples. The deletion element is located to position 1,532,307–1,532,315 on chromosome 13 (5’-TTCCATCGT-3’ on the plus strand; highlighted in black). Samples #4 and #5 have tae1-consistent reads without the motif mapped across the region. These are aligned with *-symbols indicating gaps in the alignment against the reference sequence. Samples #2 and #3 do not have well mapped data across the region and may also lack the element. (B) Read data for 10 *scutellata* samples. Samples #2 and #3 have tae1-consistent reads without the motif mapped across the region. Sample #10 does not have well mapped data across the region and may lack the element. (C) Read data for 10 *adansonii* samples. Samples #5 and #10 tae1-consistent reads without the motif mapped across the region. Samples #1 and #7 does not have well mapped data across the region and may lack the element. (D) Table summarizing the short read evidence for the occurrence of tae1 across the three subspecies. Reads aligned with gaps across the deletion motif were detected in 2 out 10 samples in all three subspecies. Additional samples may also have the deletion. The deletion is not fixed in the Cape bees.
(PDF)

S1 Table Fixed SNPs between Cape bees and the African background population. (XLS)

S2 Table Top 1000 SNPs ranked for the Composite Selection Score (CSS) between Cape bees and the African background population. (XLS)

S3 Table Information about the 97 accessions associated with the top 1000 CSS SNPs. (XLS)

S4 Table Significantly enriched Gene Ontology terms detected among the *Drosophilia* orthologs of the candidate accessions. (XLS)
