# Supplementary figures and images for: Identification of Multiple Loci Associated with Social Parasitism in Honeybees
Source: PLoS Genet. 2016 Jun 9;12(6):e1006097. doi: 10.1371/journal.pgen.1006097 (PMC4900560; doi:10.1371/journal.pgen.1006097)

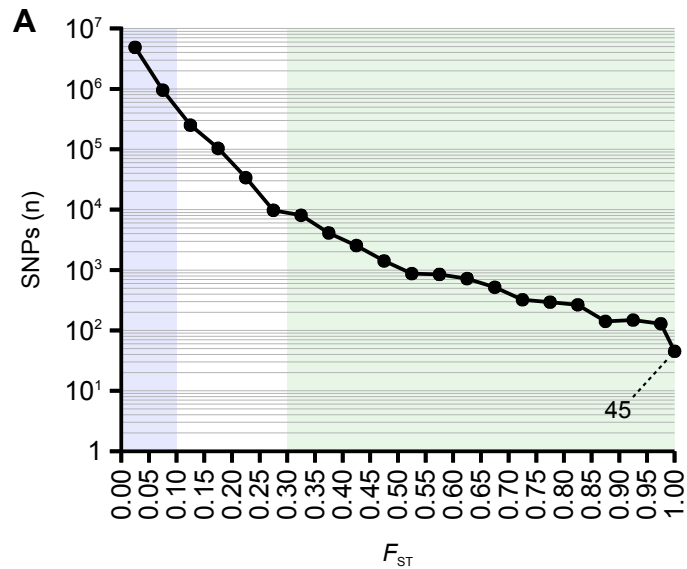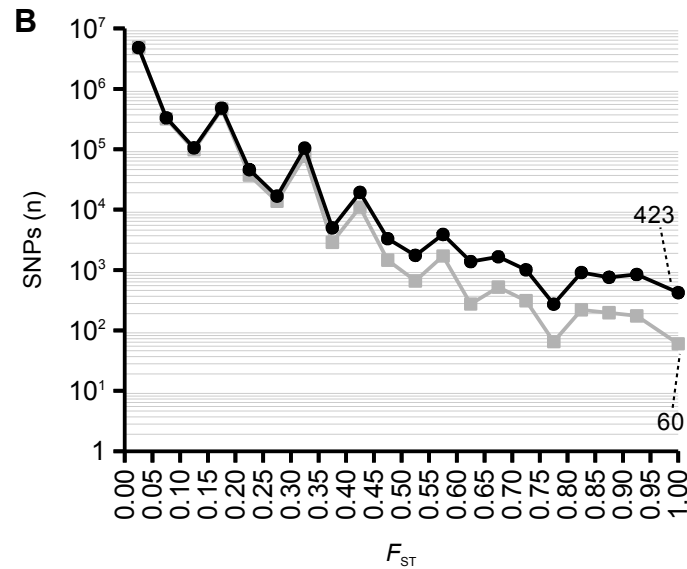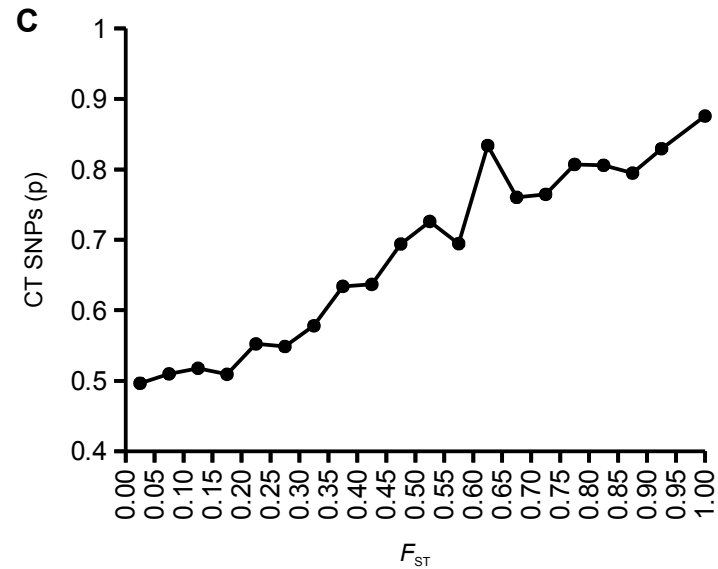

Supplement: S1 Fig — (A) FST was computed for every SNP segregating between the Cape bee (capensis, n = 10) and the scutellata + adansonii (SA; n = 20) background population (n = 6,245,176) and counted for FST bins of 0.05. The distribution is dominated by variants segregating at similar frequencies between the two groups: 93.3% of SNPs (n = 5.89 x 10^6^) have FST values below 0.1 (blue area) whereas 0.33% of SNPs (n = 20,460) have FST values above 0.3 (green area). 45 SNPs in the dataset are fixed between the two groups. (B) FST was computed for every SNP between the western Cape Town subpopulation (CT; n = 5) and SA (n = 20) (n = 5,934,995; black line) and the eastern Port Elizabeth subpopulation (PE; n = 5) and SA (n = 5,907,010; grey line) individually, and binned as in (A). The PE vs SA comparison has significant overrepresentation of low FST SNPs (FST = 0.00–0.005; 4,880,183 vs 4,810,196; p<1e-5, chi-squared test), whereas the CT vs SA comparison have significantly more SNPs segregating at every bin with FST>0.05 (p<1e-5, chi-squared test). (C) The individual counts from (B) were combined and the proportion of CT vs SA variants segregating at each FST interval was computed. For low FST variants (FST<0.3), CT SNPs make up less than 60% of SNPs (proportion CT SNPs per bin ranges from 0.496 to 0.549), whereas they are increasingly overrepresented for higher FST variants (FST>0.5; proportion CT SNPs per bin 0.694–0.856). (PDF) [file pgen.1006097.s001.pdf]

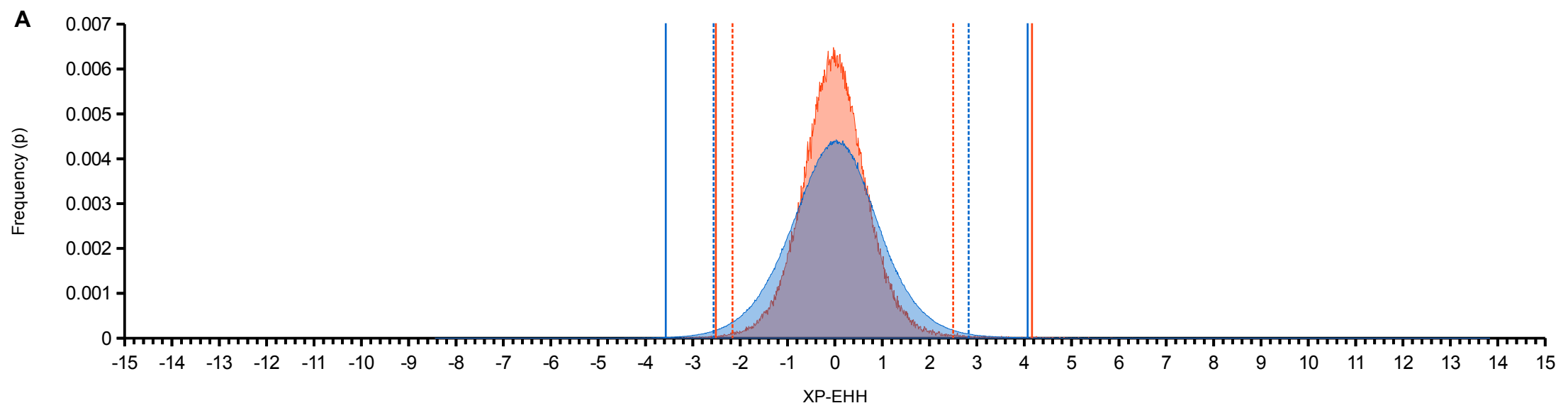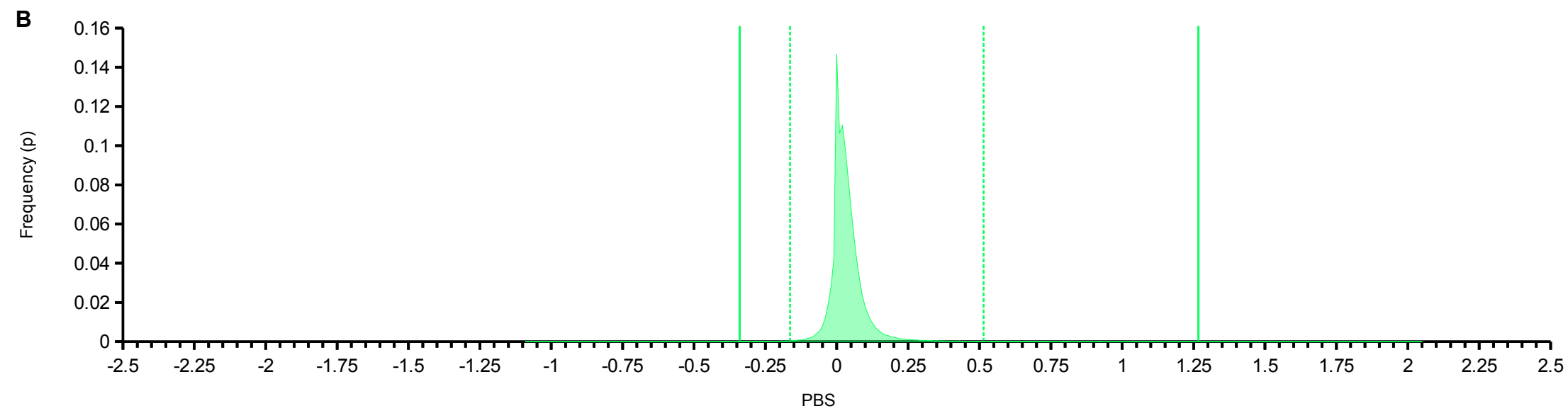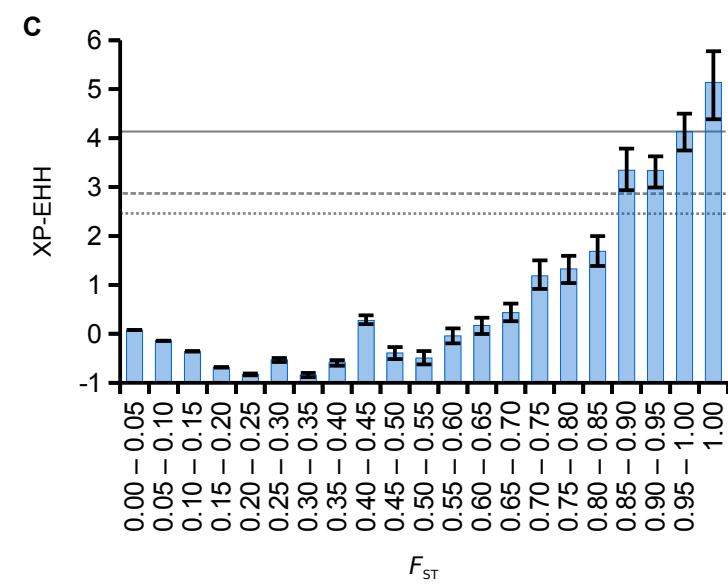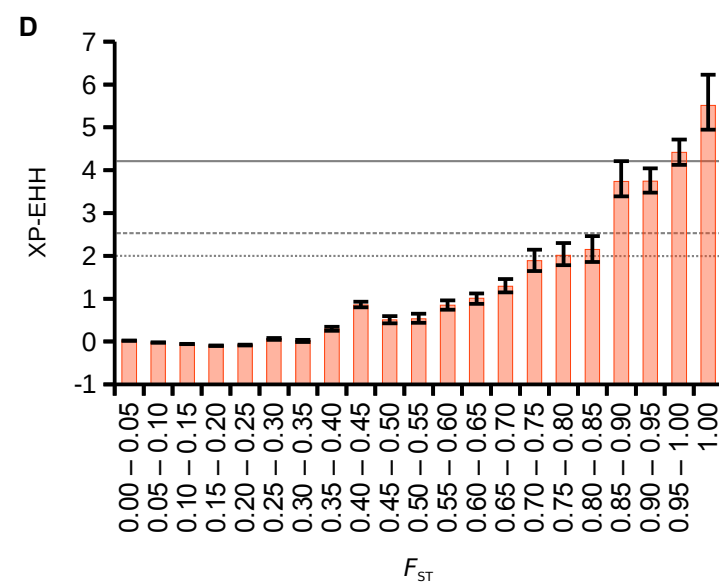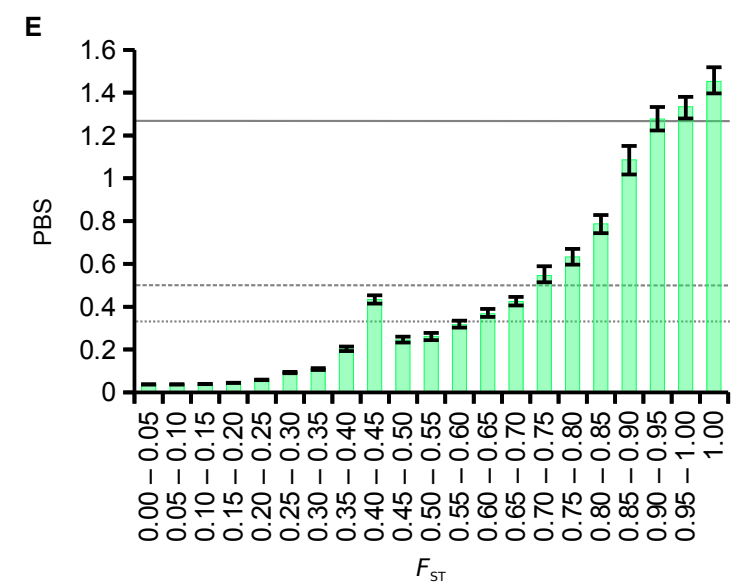

Supplement: S2 Fig — (A) The XP-EHH was estimated for SNPs with MAF>0.02 (n = 6,196,550) using the program selscan ([28]) and binned (units of 0.01; blue distribution). The average XP-EHH for was also computed for 1kbp windows and binned (n = 188,088; units of 0.01; red distribution). The distribution is centered around 0 (mean XP-EHH = 0.007). The upper and lower 99.9% and 99.5% percentiles were identified from the empirical distribution of SNP and window XP-EHH estimates. These are more extreme when associated with the Cape bees (XP-EHH>0), compared to the background population (scutellata + adansonii; XP-EHH<0). 99.9% percentiles for SNPs (blue solid lines): 4.12 vs -3.46. 99.5% percentiles for SNPs (blue dashed lines): 2.9 vs -2.74. 99.9% percentiles for windows (red solid lines): 4.19 vs -2.72. 99.5% percentiles for SNPs (blue dashed lines): 2.53 vs -2.06. (B) The population branch statistic (PBS) was estimated for 1kbp windows across the genome (n = 189,053). The distribution is slightly skewed towards higher PBS in the Cape bees than the background population (mean PBS = 0.039). Upper and lower 99.9% and 99.5% percentiles were identified from the empirical distribution of PBS estimates. These are more extreme when associated with the Cape bees (PBS>0), compared to the background population (PBS<0). 99.9% percentiles (green solid lines): 1.27 vs -0.34. 99.5% percentiles (blue dotted lines): 0.51 vs -0.16. (C) The XP-EHH of SNPs was cross-referenced with the FST computed from the allele frequency differences between the Cape bees and the scutellata + adansonii background population. SNPs were binned for FST (units of 0.05). For every FST class, the mean XP-EHH was computed and 95% confidence intervals were computed from 200 bootstrap replicates. Each class was compared to the upper percentiles retrieved from the empirical XP-EHH distribution compiled in (A), which are associated with long haplotypes in the Cape bees. The 99.9% percentile is 4.12 (grey solid line); the 99.5% percent [file pgen.1006097.s002.pdf]

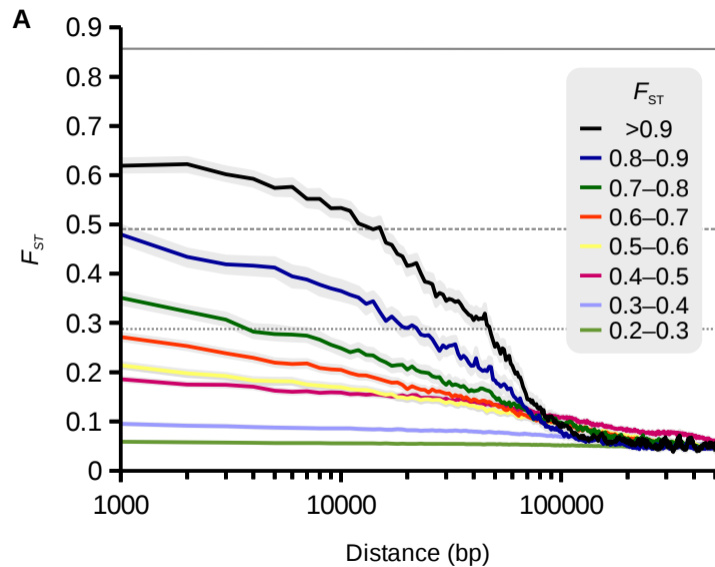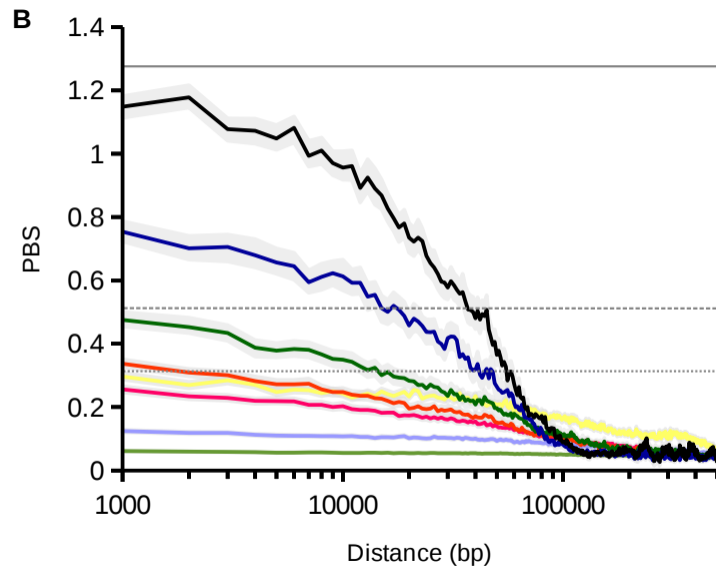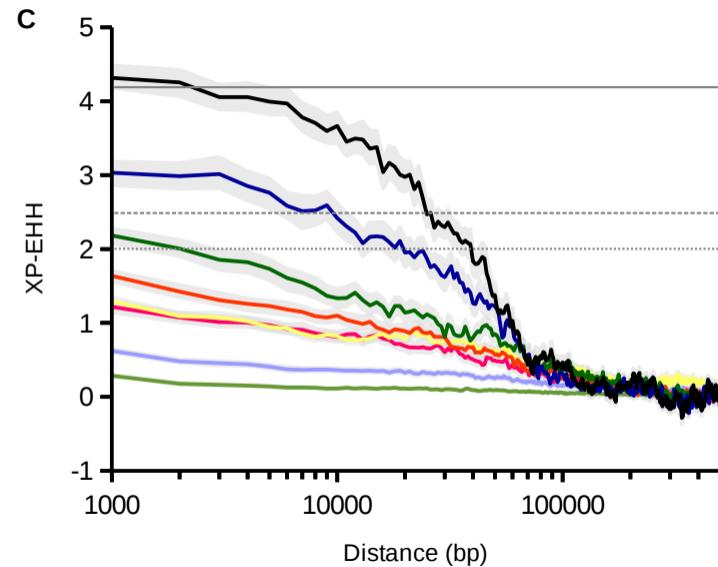

Supplement: S3 Fig — (A) Every SNP with FST>0.2 and minimal evidence for extended haplotype homozygosity (XP-EHH) in the Cape bees (XP-EHH>0) was put in FST bins of 0.10. The linked divergence was traced around each SNP by computing FST across 1kbp windows for up to 500kbp to either side of each SNP. 95% confidence intervals were computed from 200 bootstrap replicates for every SNP FST class and distance. High FST SNPs appear to be clustered together in peaks: 3kbp away from the most highly differentiated SNPs (FST>0.9), the window-based divergence is higher (0.6) than for SNPs with FST = 0.7–0.8 (0.3; p<0.01), indicative of higher density of highly differentiated SNPs around the former. The decay of linked signals was compared against genome-wide percentiles: top 0.1% (solid line), top 0.5% (dashed line), 1% (dotted line). Linked divergence signals extend longest around high FST SNPs: divergence drops to the top 1% level after about 50kbp for SNPs at FST>0.9, compared to about 20kbp for SNPs at FST = 0.8–0.9 and 4kbp for SNPs at 0.7–0.8. The most differentiated SNPs therefore appear to be located in regions with the highest and widest linked divergence. (B) Tracing the decay of the population branch statistic (PBS) using the same procedure and SNPs as in (A). SNPs with high FST are clustered for the PBS and correlated with the widest linked signals: 3kbp away from SNPs with FST>0.9, the PBS in linked regions is significantly elevated compared to other SNPs with FST = 0.7–0.8 (1.1 vs 0.45; p<0.01) and it takes about 60kbp for the PBS to drop to the top 1% level around FST>0.9 SNPs, compared to 40kbp for SNPs at FST = 0.8–0.9 and 15kbp for SNPs at 0.7–0.8. The most differentiated SNPs are hence located in regions that with a long branch against both other African and European bees. (C) Tracing the decay of the XP-EHH statistic using the same procedure and SNPs as in (A). XP-EHH was estimated for SNPs with MAF>0.02. The average XP-EHH was computed for every 1kbp window up to 500kbp away f [file pgen.1006097.s003.pdf]

**A**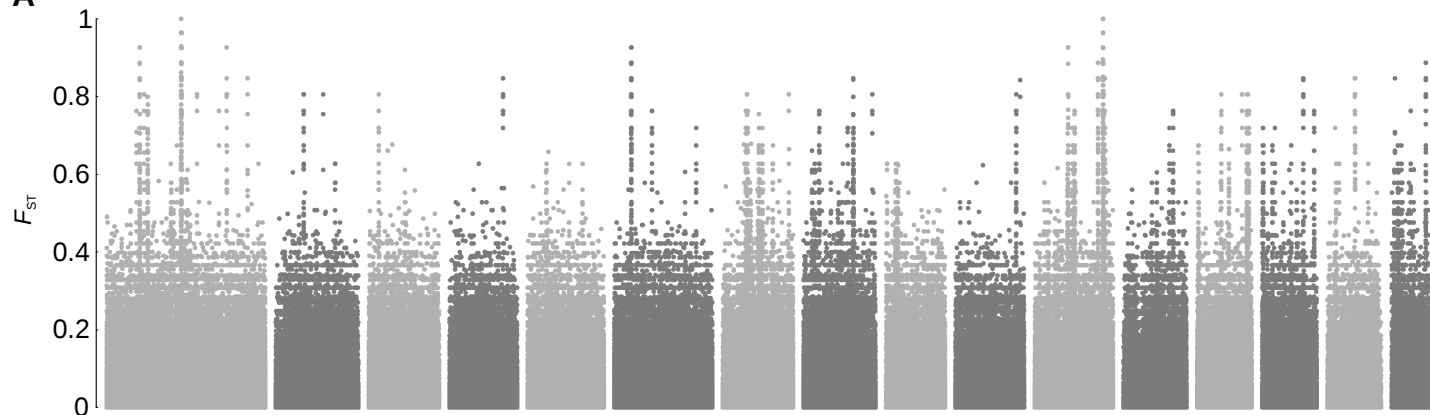**B**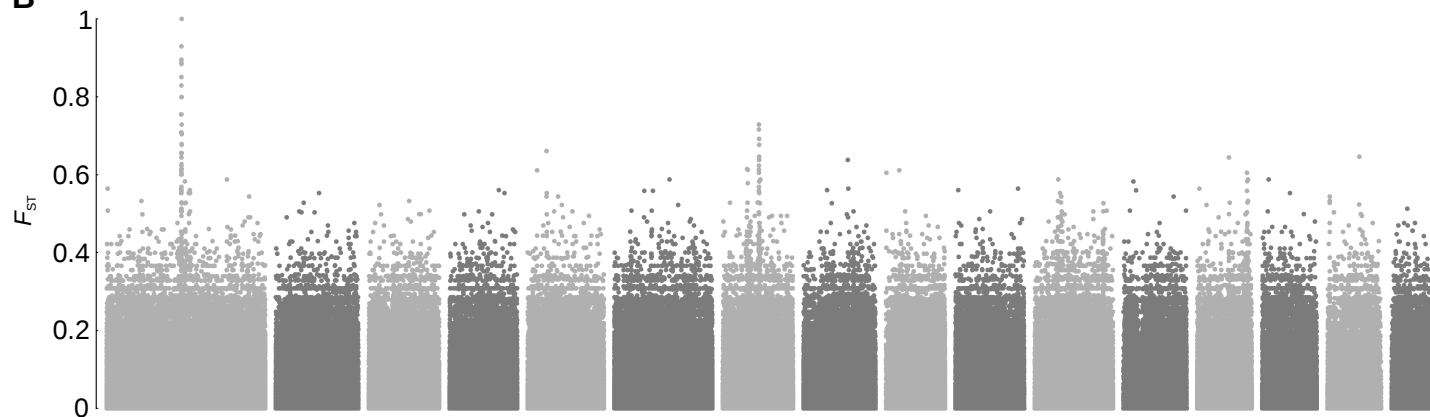**C**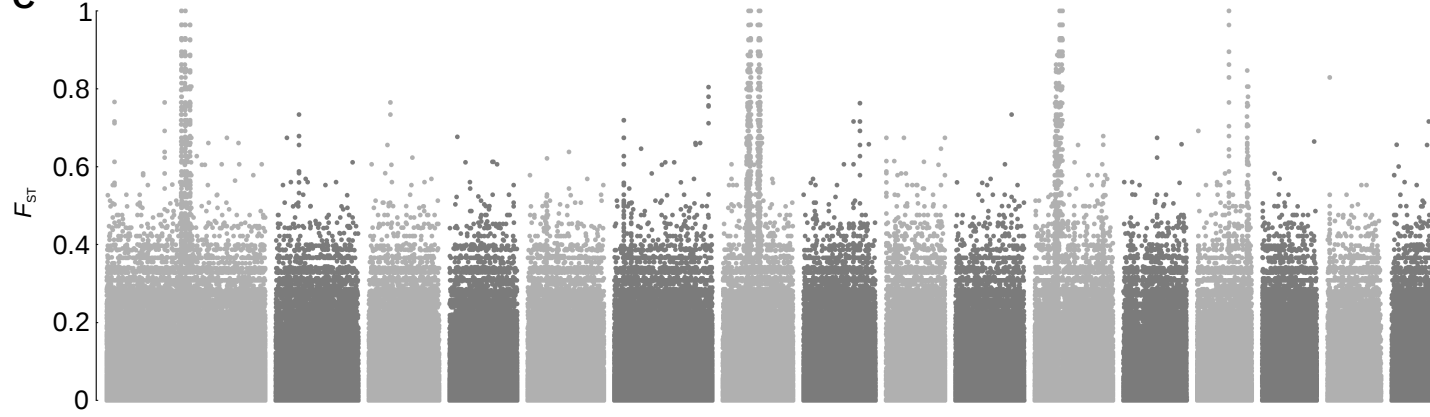

1

2

3

4

5

6

7

8

9

10

11

12

13

14

15

16

Chromosome

Supplement: S4 Fig — The fixation index (FST) was computed for every SNP segregating between each subspecies and a combined population consisting of the other two African subspecies (further described in Fig 2). (A) A. m. capensis vs adansonii + scutellata. The main Cape bee scan has FST peaks (FST>0.8) distributed across 13 chromosomes. (B) A. m. scutellata vs adansonii + capensis. The scutellata scan detects a single FST peak (FST>0.8) on chromosome 1. (C) A. m. adansonii vs capensis + scutellata. The adansonii scan detects FST peaks (FST>0.8) distributed across 6 chromosomes. Most of these SNPs are clustered on chromosomes 7 and 11. (PDF) [file pgen.1006097.s004.pdf]

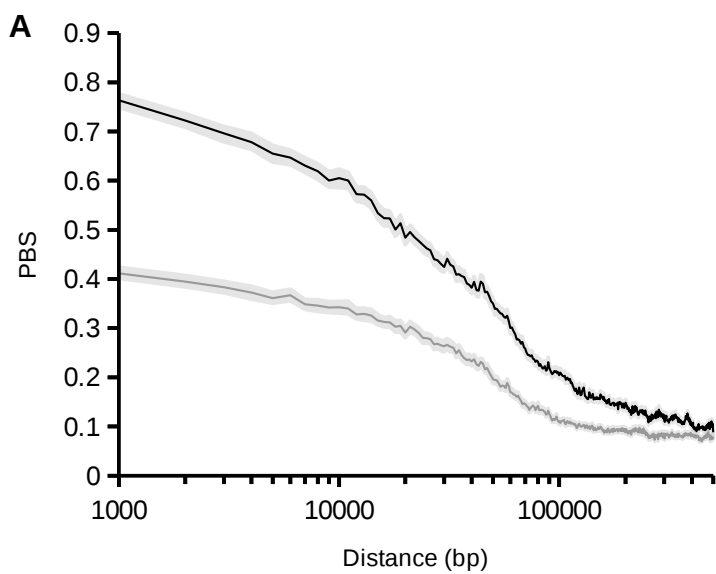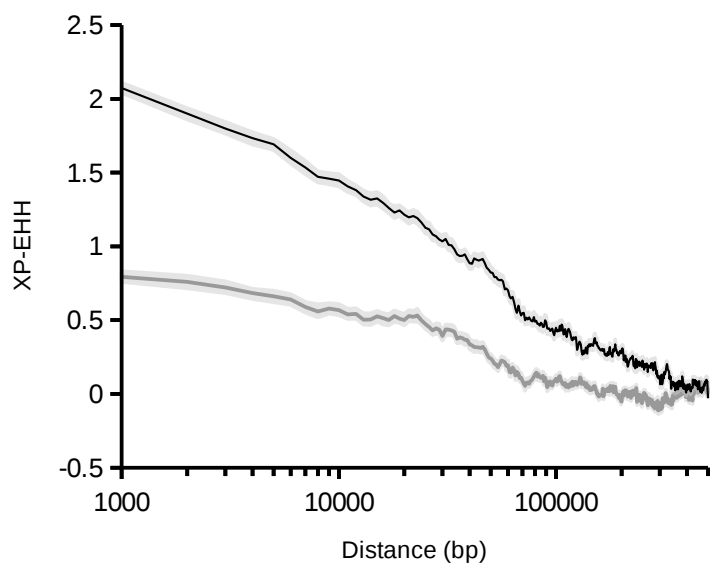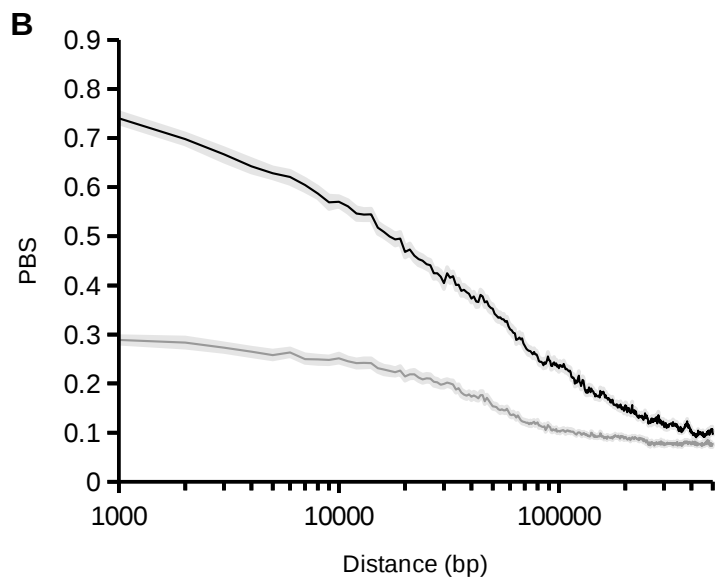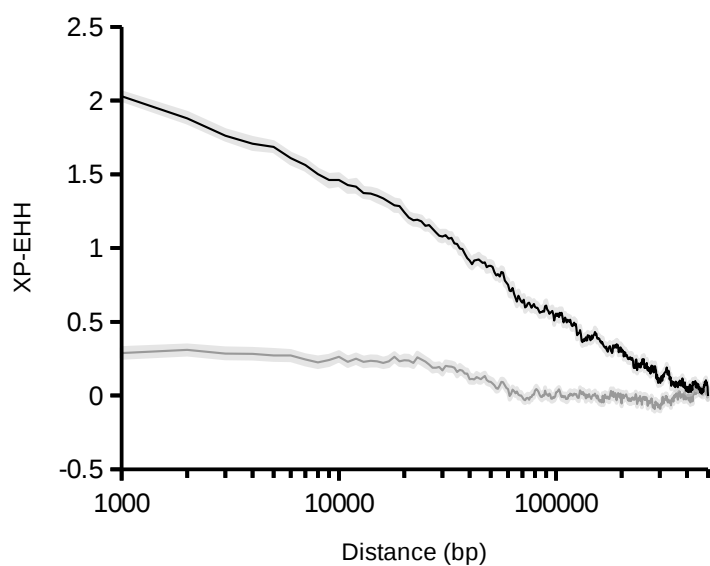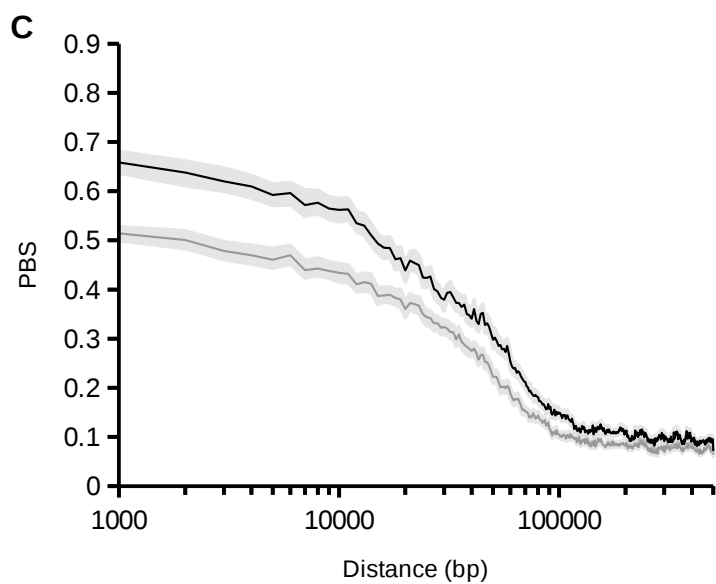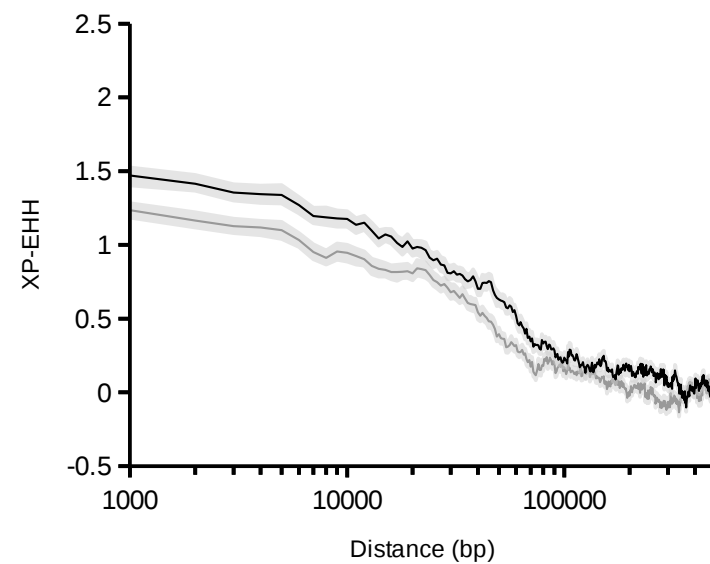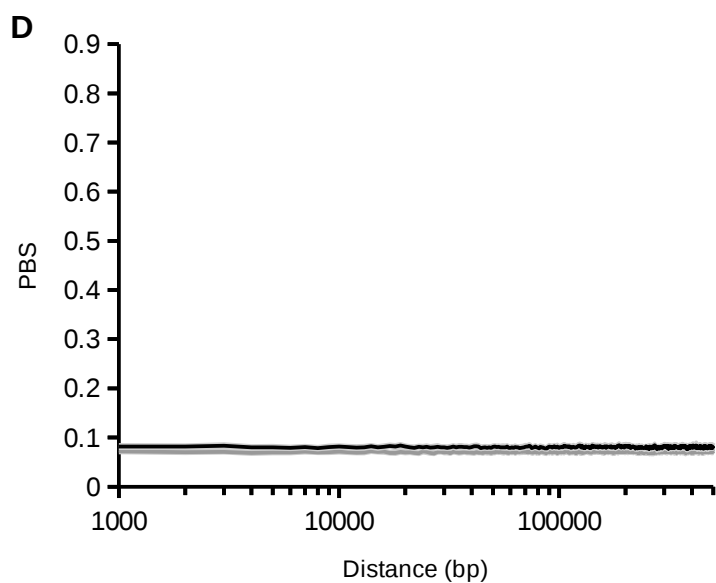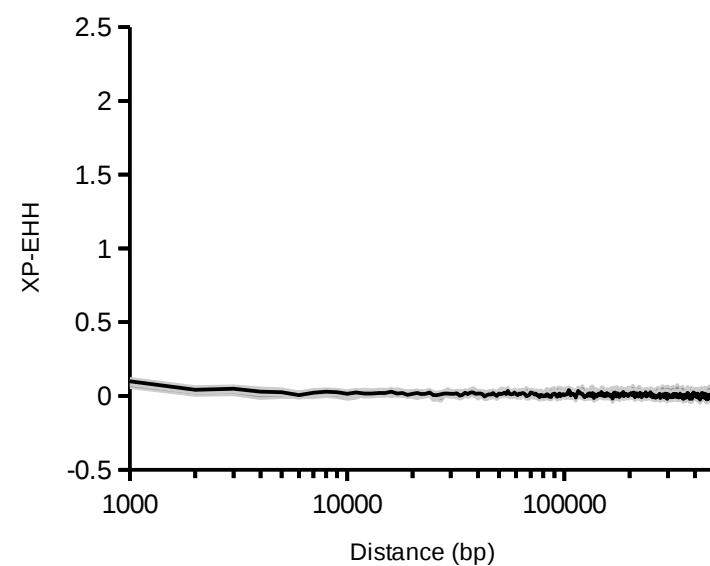

Supplement: S5 Fig — (A) SNPs segregating between the Cape bees and the African background population (scutellata + adansonii) were sorted for FST and the top 0.1% (n = 6245) were filtered for minimal evidence for extended haplotype homozygosity in the Cape bees (XP-EHH>0) to make a candidate set of variants with trending evidence for selection in the Cape population (n = 2917). The decay of the population branch statistic (PBS) and XP-EHH, respectively, was traced in 1kbp windows for the western Cape town (CT; black line) and eastern Port Elizabeth (PE; grey line) subpopulations for up to 500kbp to either side of every such SNP. 95% confidence intervals (grey) were computed from 200 bootstrap replicates at every distance. The mean PBS and XP-EHH signals are distinctly stronger in CT compared to PE close to these SNPs: at 3kbp away from a SNP, PBSCT is almost twice as high as PBSPE (0.70 vs 0.38; p<0.01) and XP-EHHCT is almost 2.5x times higher than for XP-EHHPE (1.80 vs 0.72; p<0.01). The linked signatures are significantly stronger in CT than in PE around these SNPs for hundreds of kilobasepairs: PBSCT>PBSPE for over 500kbp (p<0.05) and XP-EHHCT>XP-EHHPE for 350kbp (p<0.05). These haplotype patterns are consistent with stronger signatures of selection in the CT subpopulation. (B) Analysis as in (A) but tracing the PBS and XP-EHH for CT and PE around outliers (n = 3820) identified specifically between the CT subpopulation and the SA population. The pattern is consistent with (A) but the difference between the CT and PE subpopulation for linked signatures of selection is further amplified when focused around these outlier SNPs: at 3kbp away from a SNP, the mean PBS for CT is 0.66 vs 0.27 for PE (p<0.01) and the mean XP-EHH for CT is 1.76 vs 0.28 for PE (p<0.01). These haplotype patterns indicate that some signatures of selection in the CT subpopulation are considerably weaker in the PE subpopulation. (C) Analysis as in (A) but tracing the PBS and XP-EHH for CT and PE around outliers (n [file pgen.1006097.s005.pdf]

**A**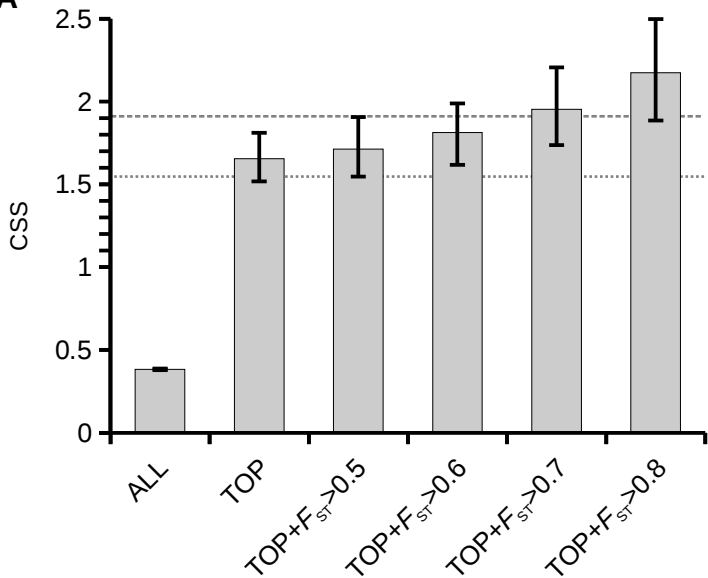

Supplement: S6 Fig — (A) Genetic distance (FST estimator of Reynolds et al. [104]), the population branch statistic (PBS) and cross population extended haplotype heterozygosity (XP-EHH) between the Cape bees and the African background population were estimated for 1kbp windows (scutellata + adansonii) across the full genome and joined into a Composite Selection Score (CSS; [24]). The window-based CSS estimates were cross-referenced with accessions by taking the full gene-body coordinates adjusted to include 2kbp of upstream and downstream sequence. Across all 13,281 accessions, the mean CSS score is 0.383. The 99% percentile for CSS is 1.546 and the 99.5% percentile for CSS is 1.912. The 97 accessions identified by taking the top 1000 SNPs ranked for their CSS (FST and XP-EHH) have significantly elevated CSS across the full gene body (CSS = 1.654; p<0.05). This set was reduced to include only accessions with SNPs above a threshold level of fixation. As these thresholds became increasingly strict, we enriched for accessions with high overall CSS scores. The 25 accessions that include putative causative variants with FST>0.8 (99.99% percentile for FST) and have a mean CSS score of 2.175, significantly higher than the top 1% of genes (p<0.05). 95% confidence intervals were generated from 200 bootstrap replicates of each class of genes. (PDF) [file pgen.1006097.s006.pdf]

A

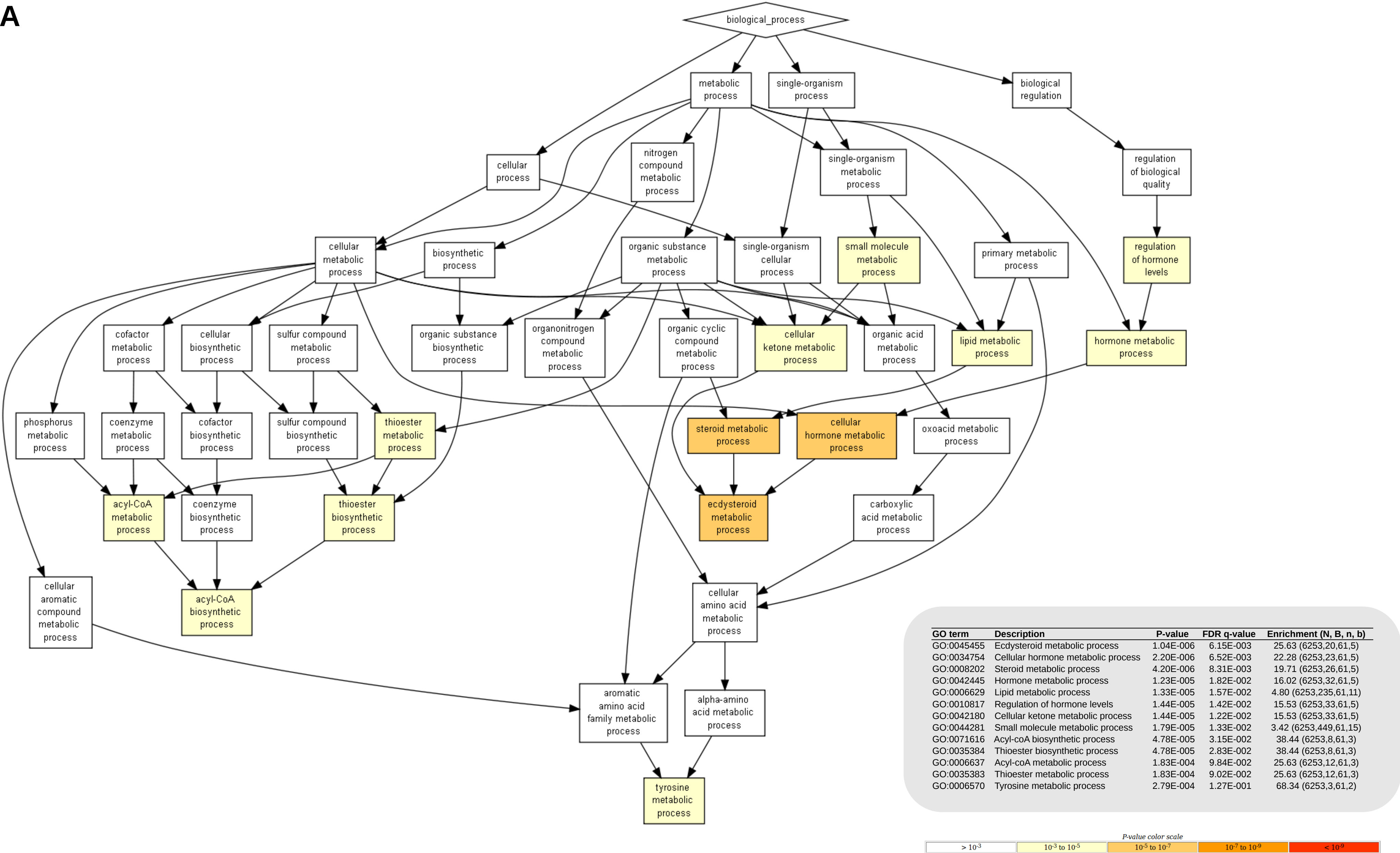

B

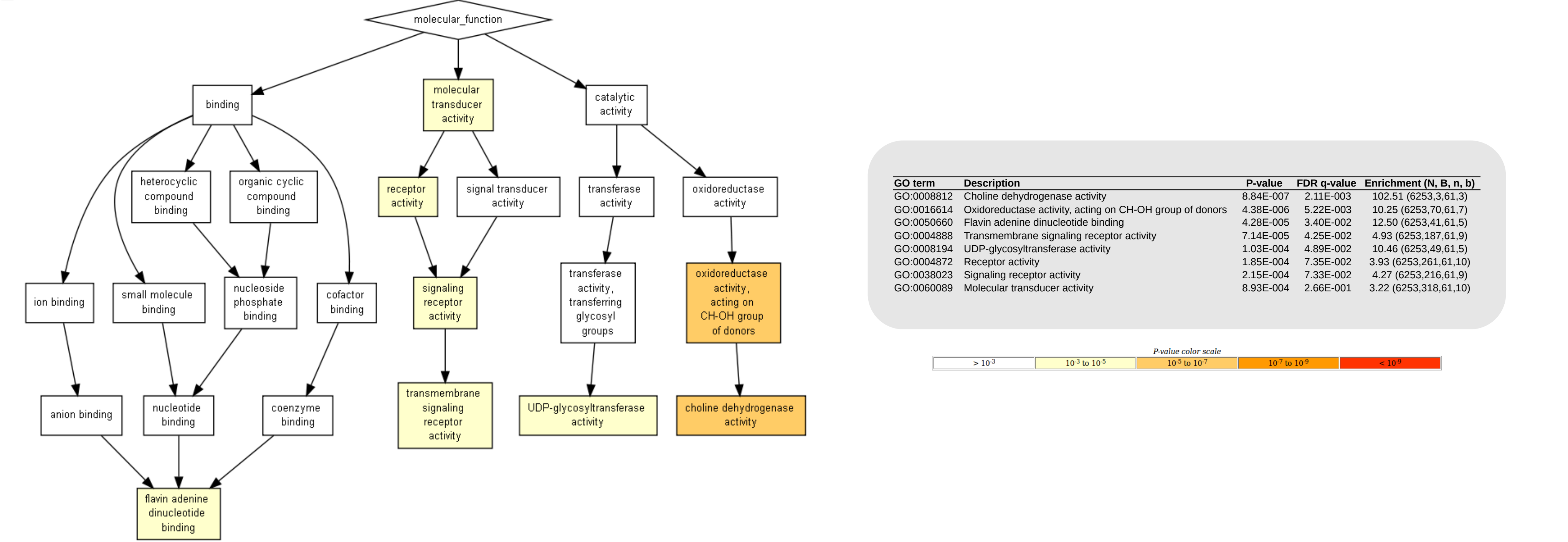

Supplement: S7 Fig — The top 1000 SNPs sorted for their Composite Selection Signal (CSS; FST+XP-EHH) located within 8kbp from the closest gene body were associated with 97 accessions in the honeybee genome (S3 Table), 73 of which had previously been matched to 68 unique Drosophila accessions using BLASTx with an e-value <0.5 (Wallberg et al. [21]). 61 of these accessions and a background set of 6253 honeybee-fly orthologues were recognized by the GOrilla platform and the candidate set was queried for significantly enriched GO-terms. (A) We detected 13 significantly enriched biological processes (p<0.05), 10 of which had a q-value <0.05 after correcting for multiple testing. The significantly enriched terms with no nested GO-terms below them are “Ecdysteroid metabolic process” (GO:0045455) and “Acyl-coA biosynthetic process” (GO:0071616). On the left: graph showing the interrelationships among GO-terms. On the right: table of GO-terms and their associated values and counts. Enrichment abbreviations are as follows: N = the total number of genes; B = the total number of genes associated with a specific GO term; n = the number of genes in in the target set; b = the number of genes in the intersection. Enrichment is taken as (b/n) / (B/N). (B) We detected 8 significantly enriched molecular functions (p<0.05), 5 of which had a q-value <0.05 after correcting for multiple testing. The significantly enriched terms with no nested GO-terms below them are “Flavin adenine dinucleotide binding” (GO:0050660), “Transmembrane signaling receptor activity” (GO:0004888), “UDP-glycosyltransferase activity”, (GO:0008194) and “Choline dehydrogenase activity” (GO:0008812). (PDF) [file pgen.1006097.s007.pdf]

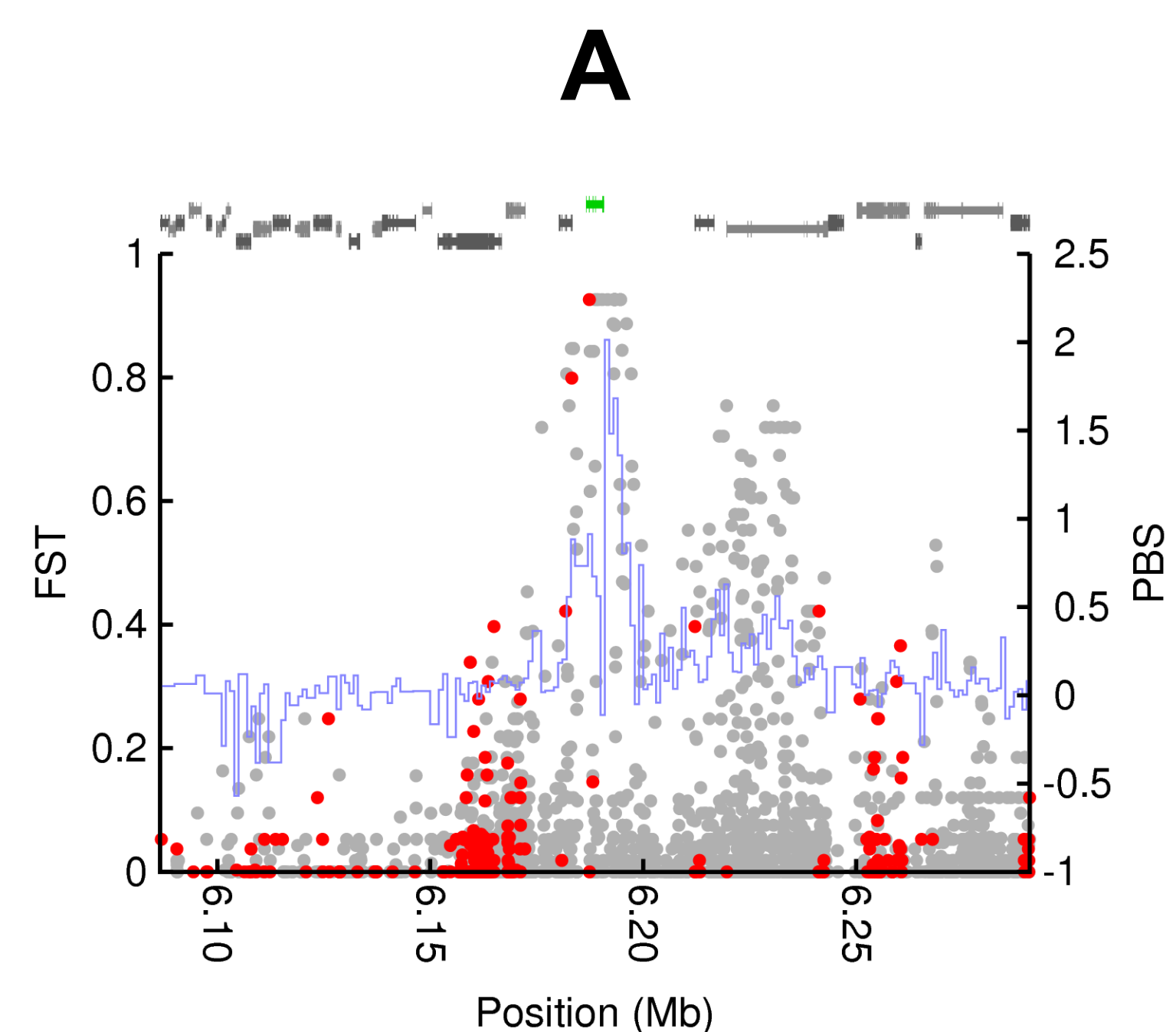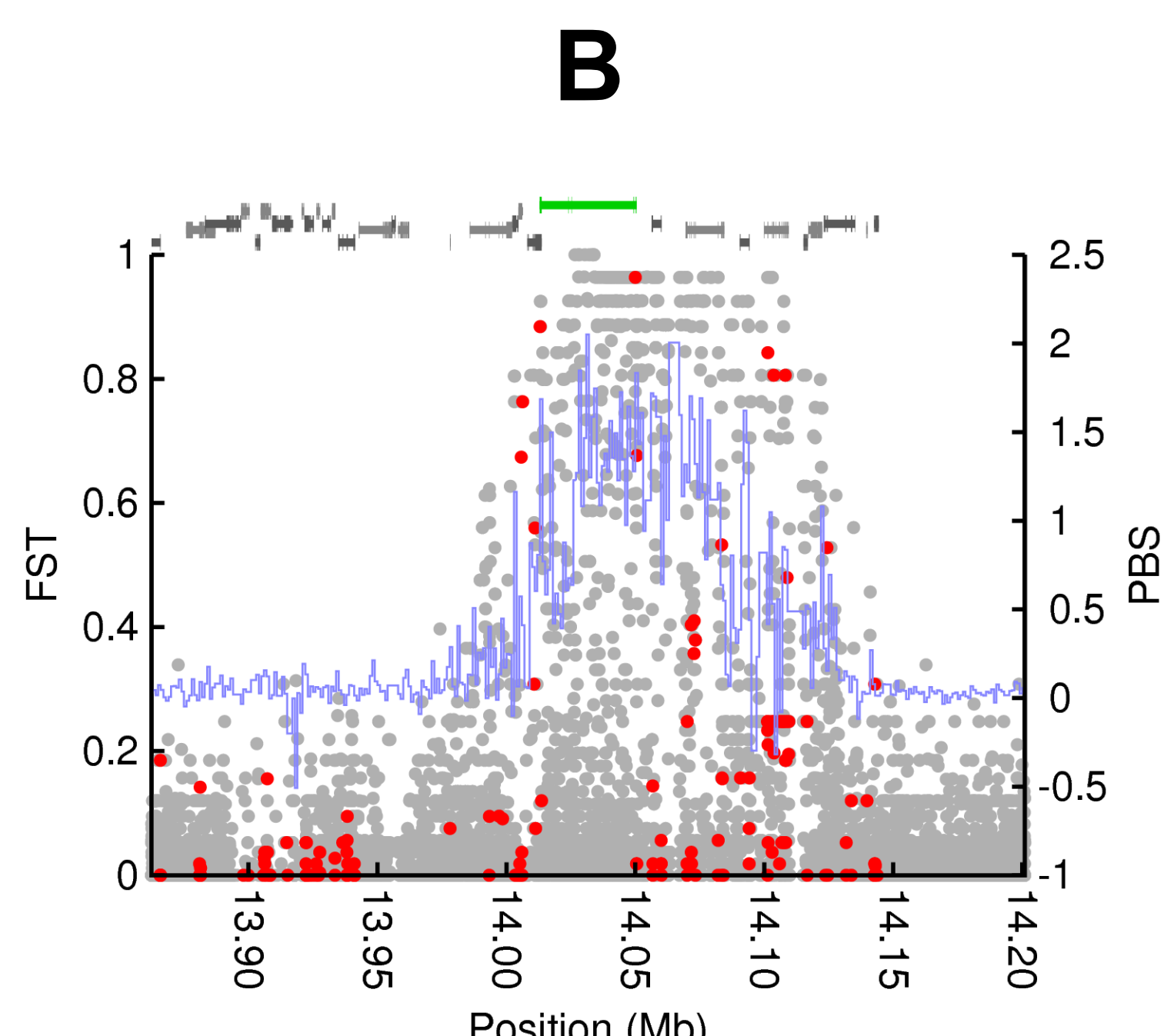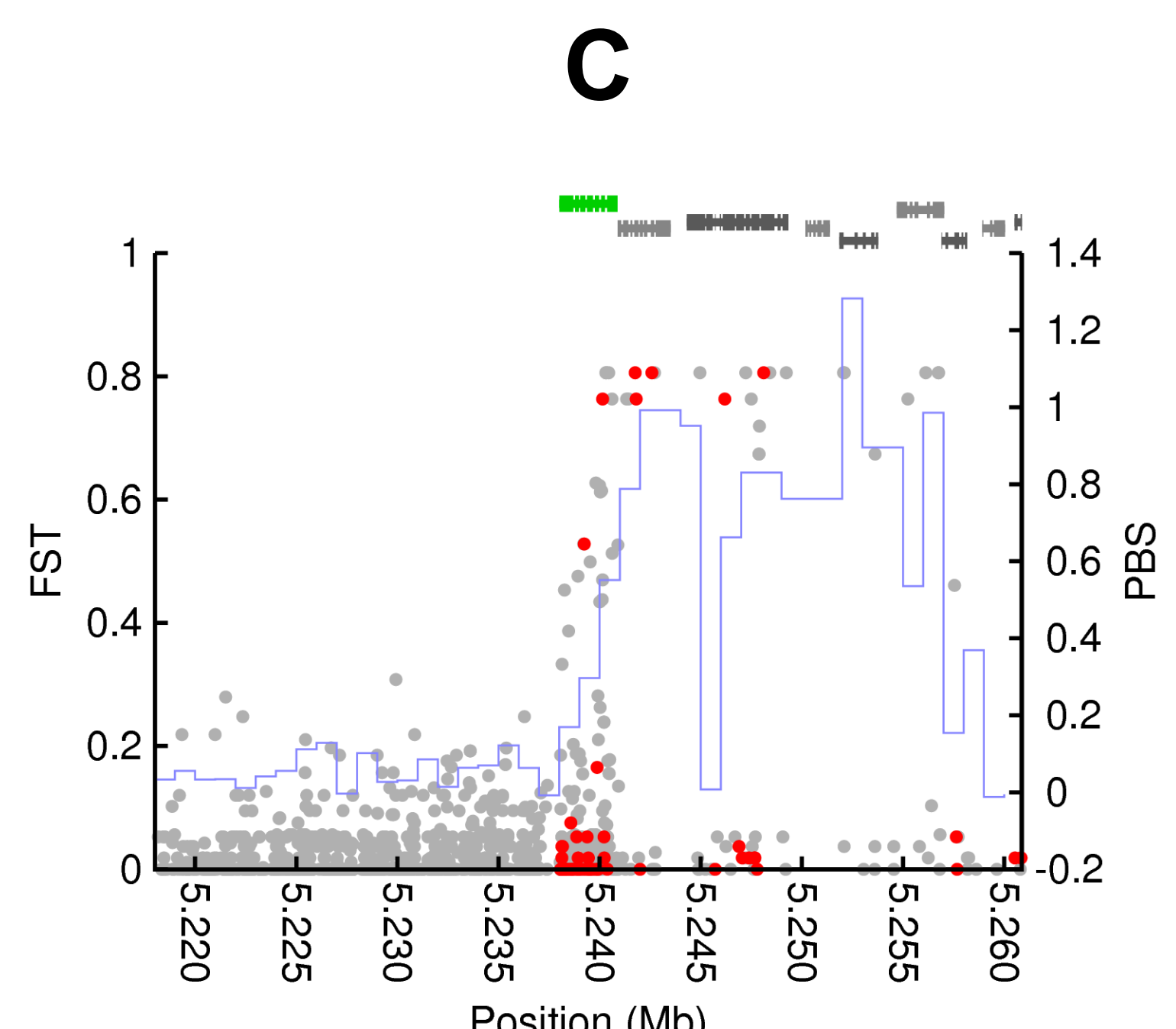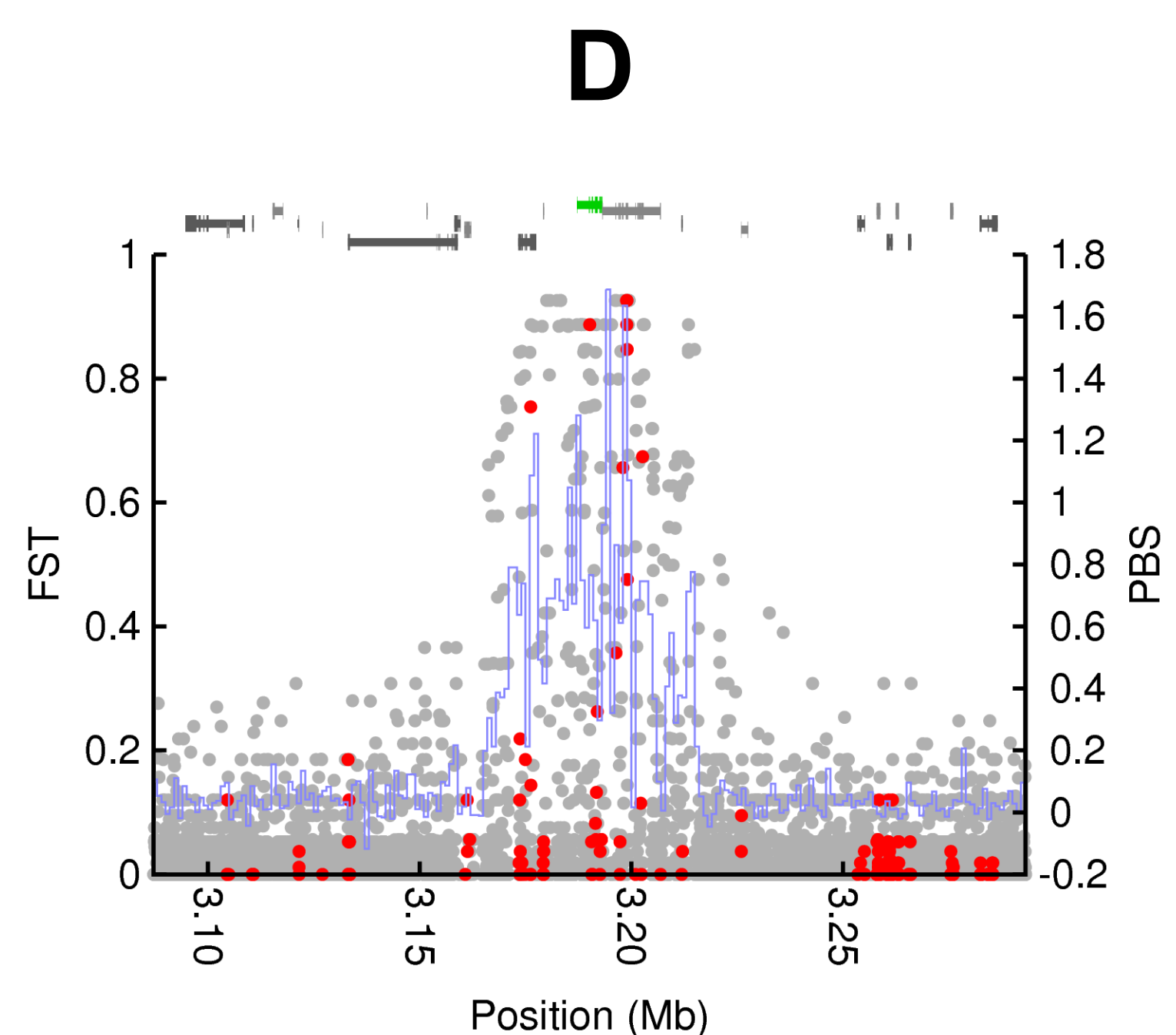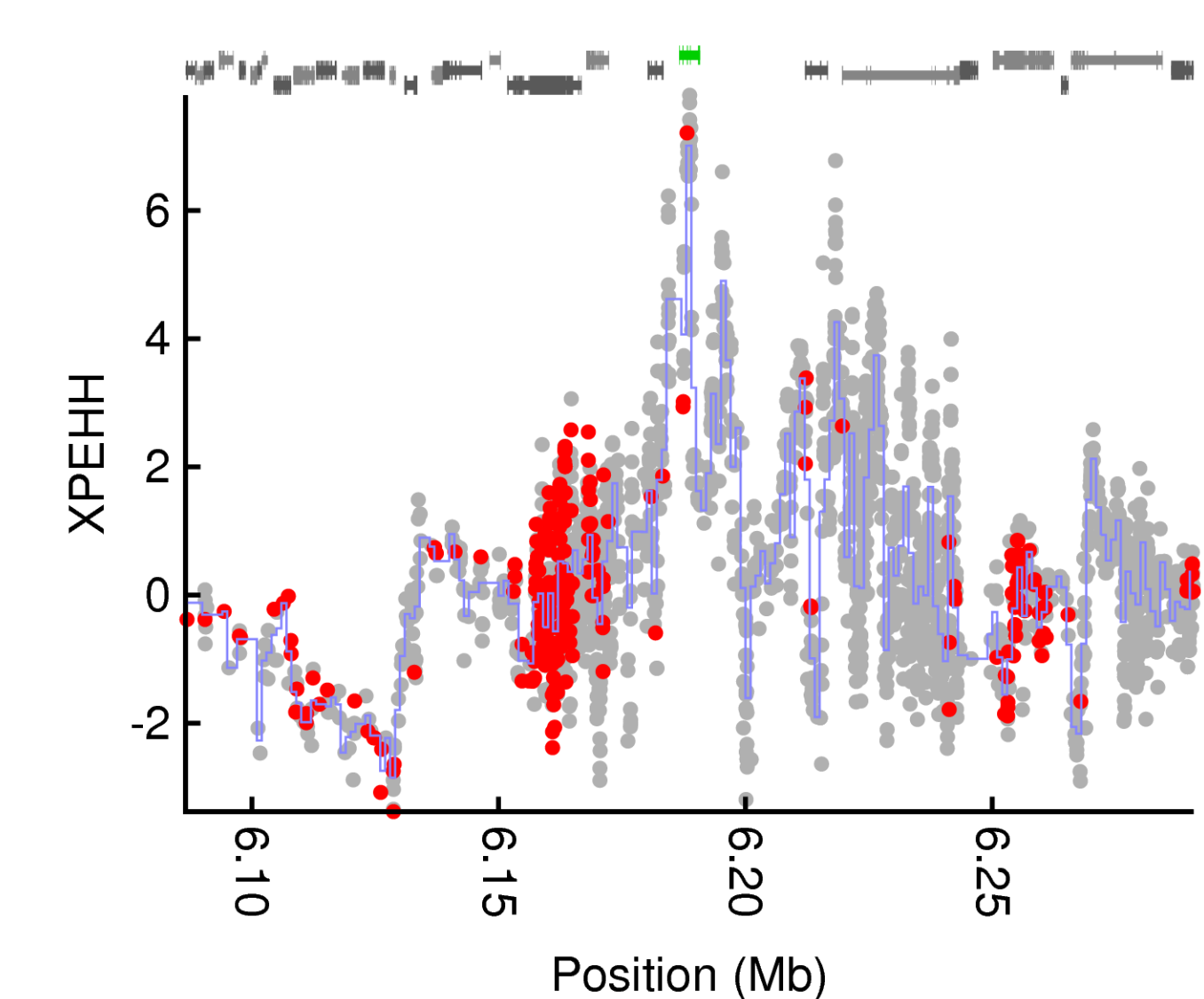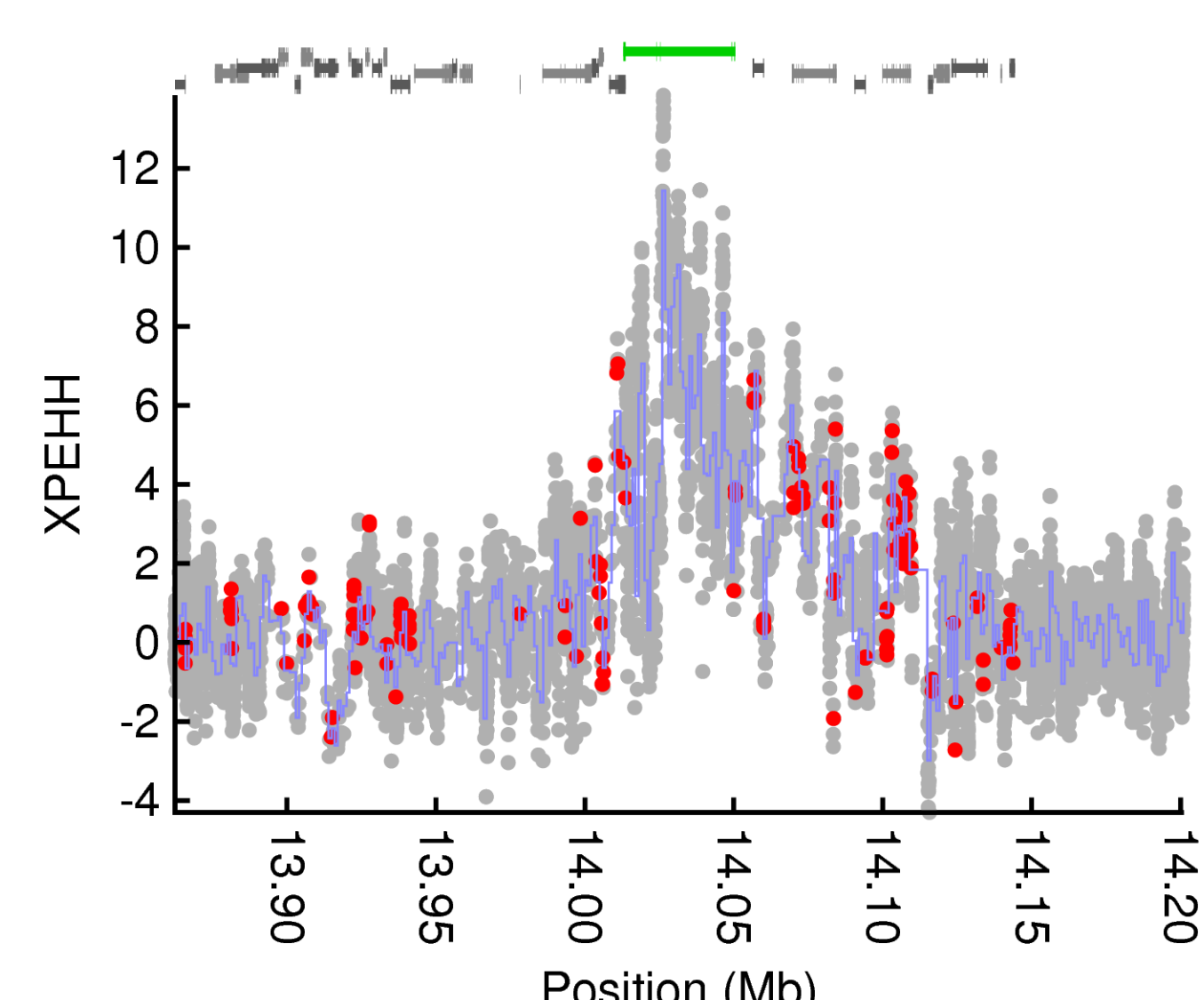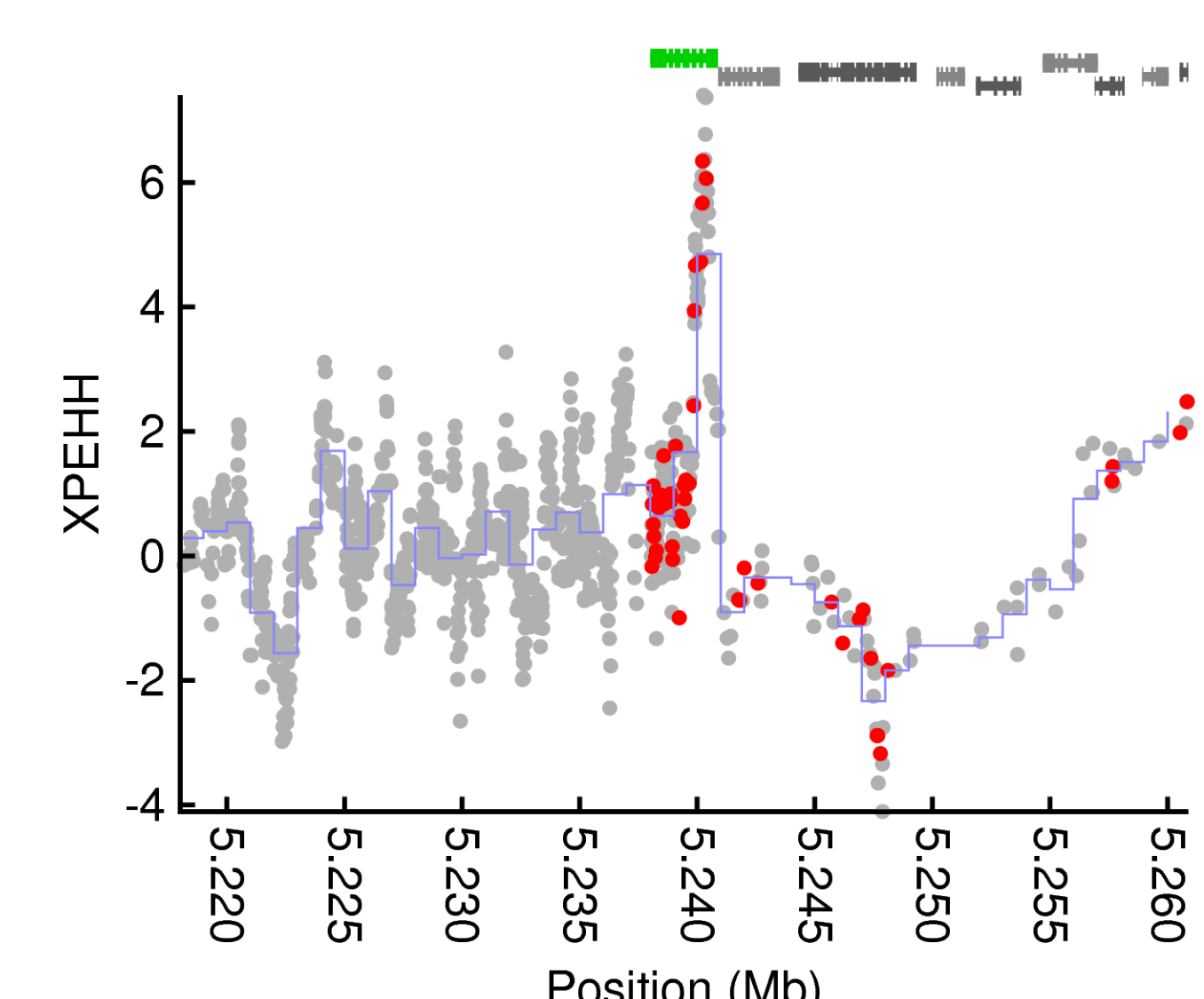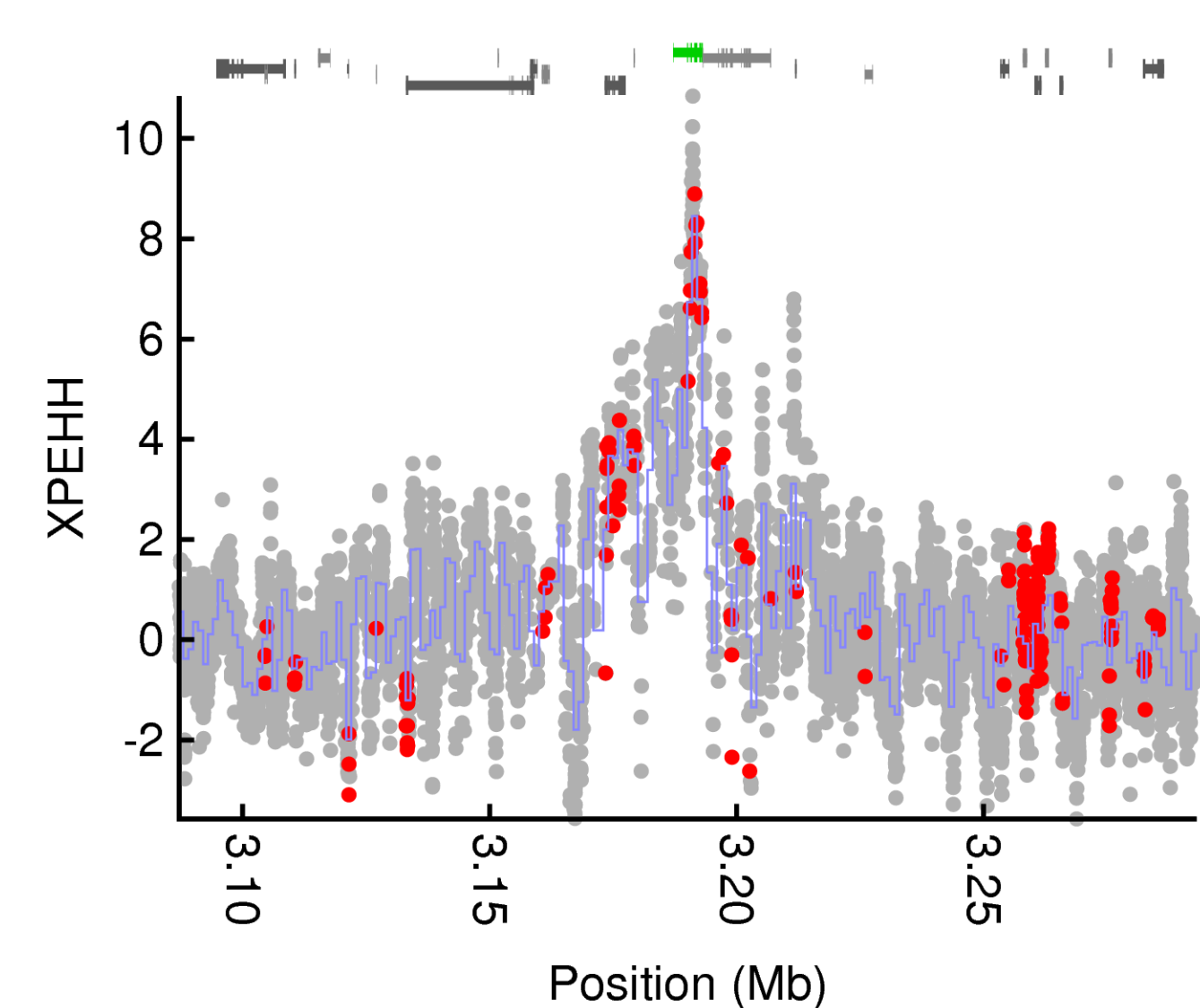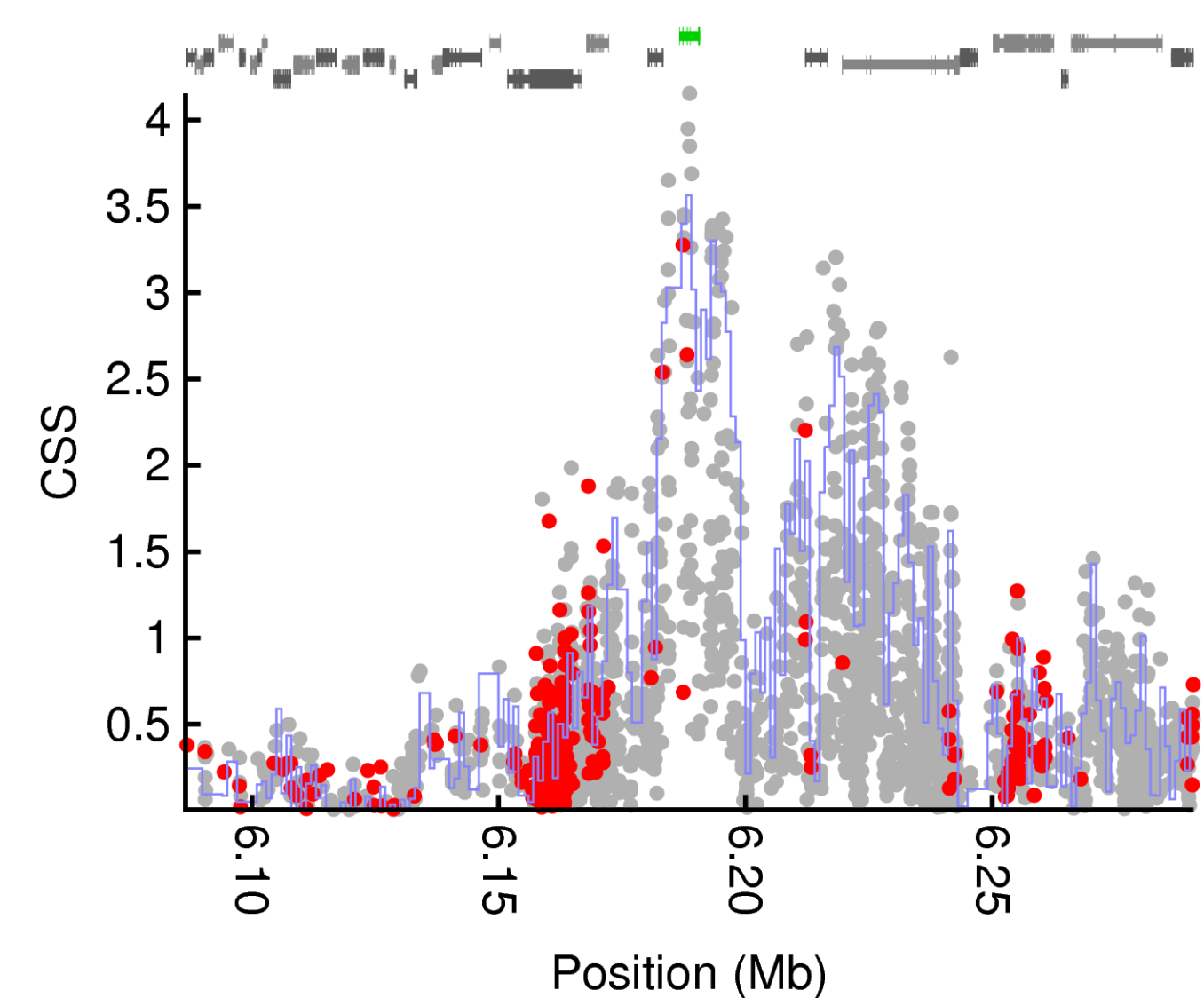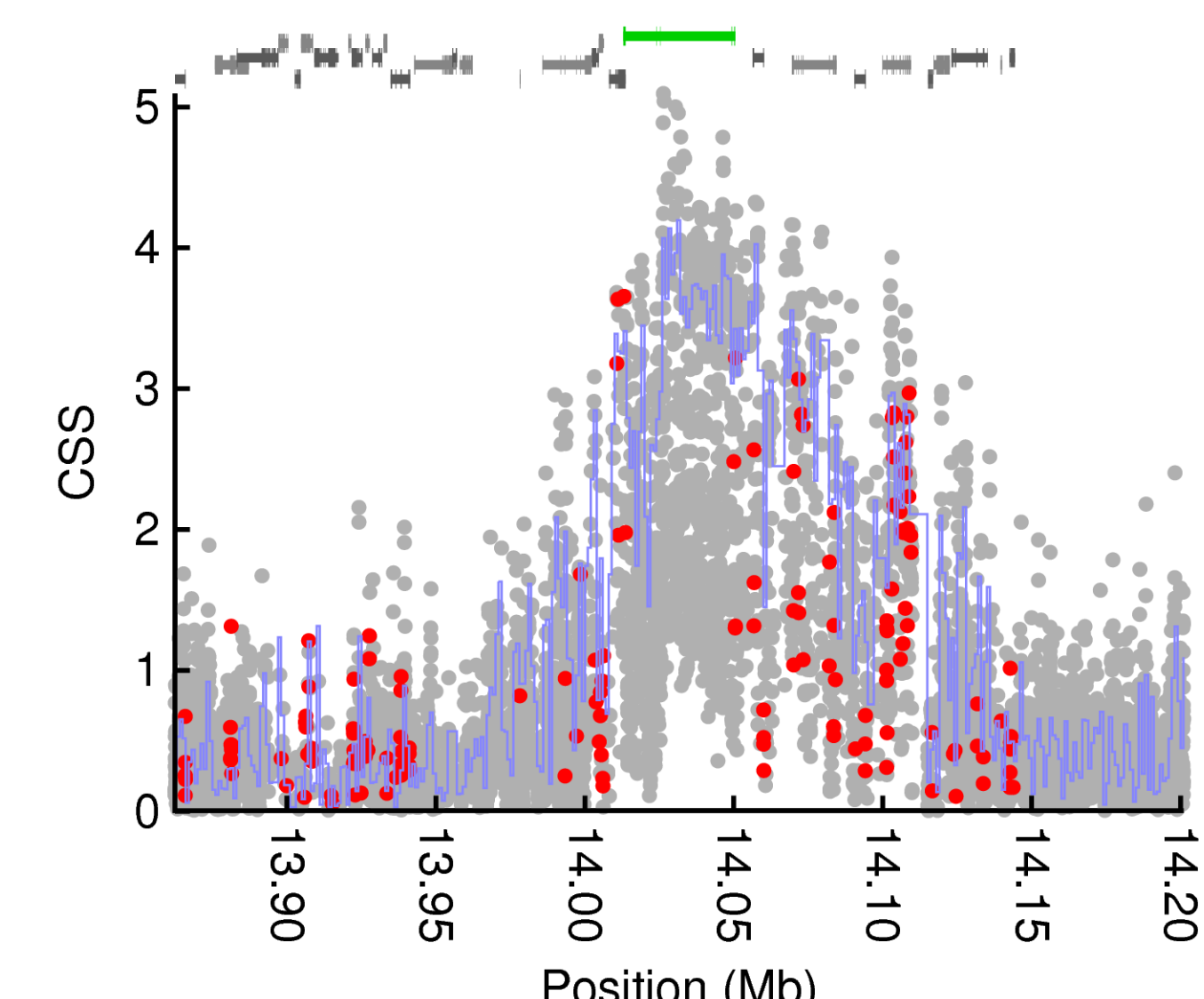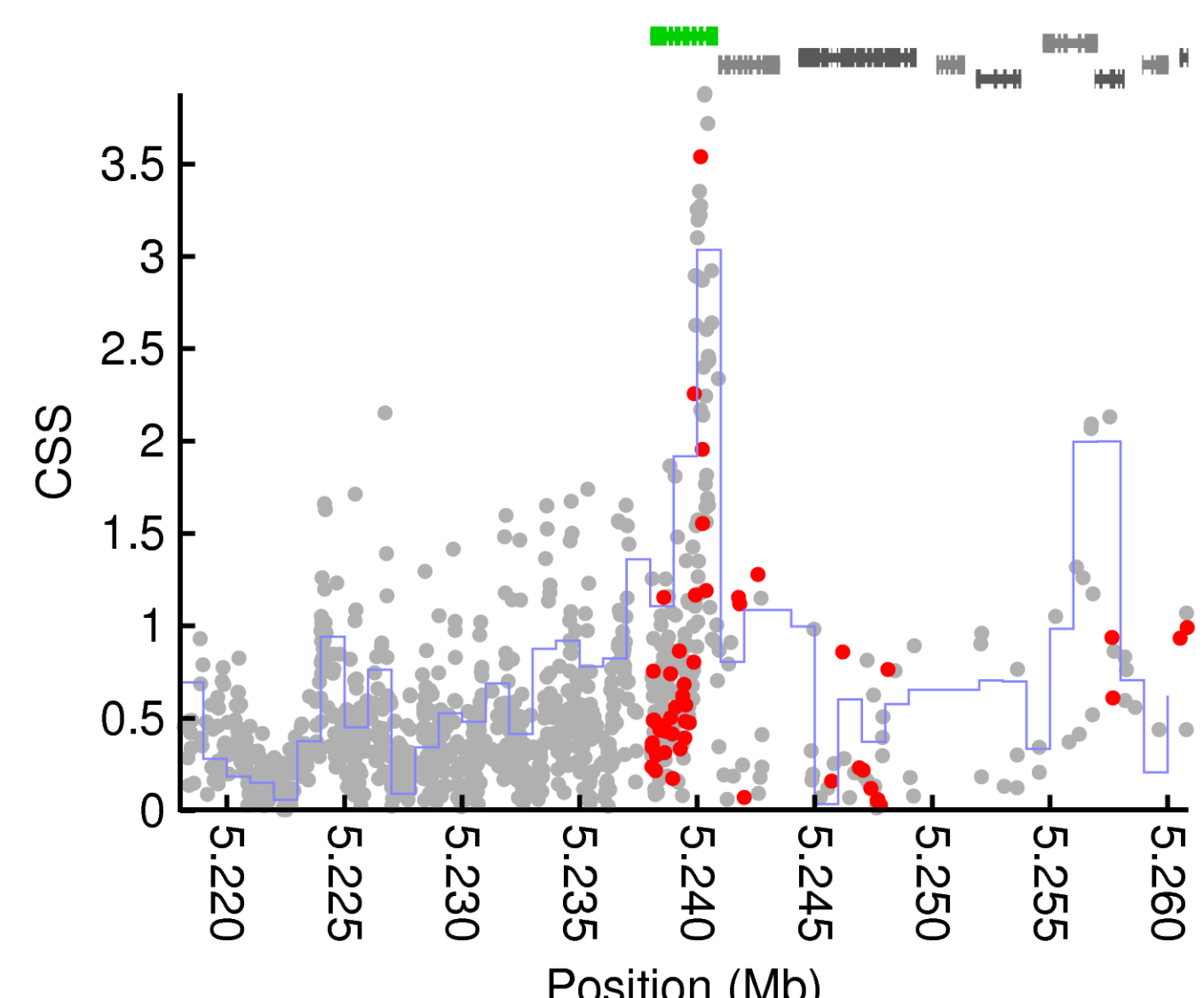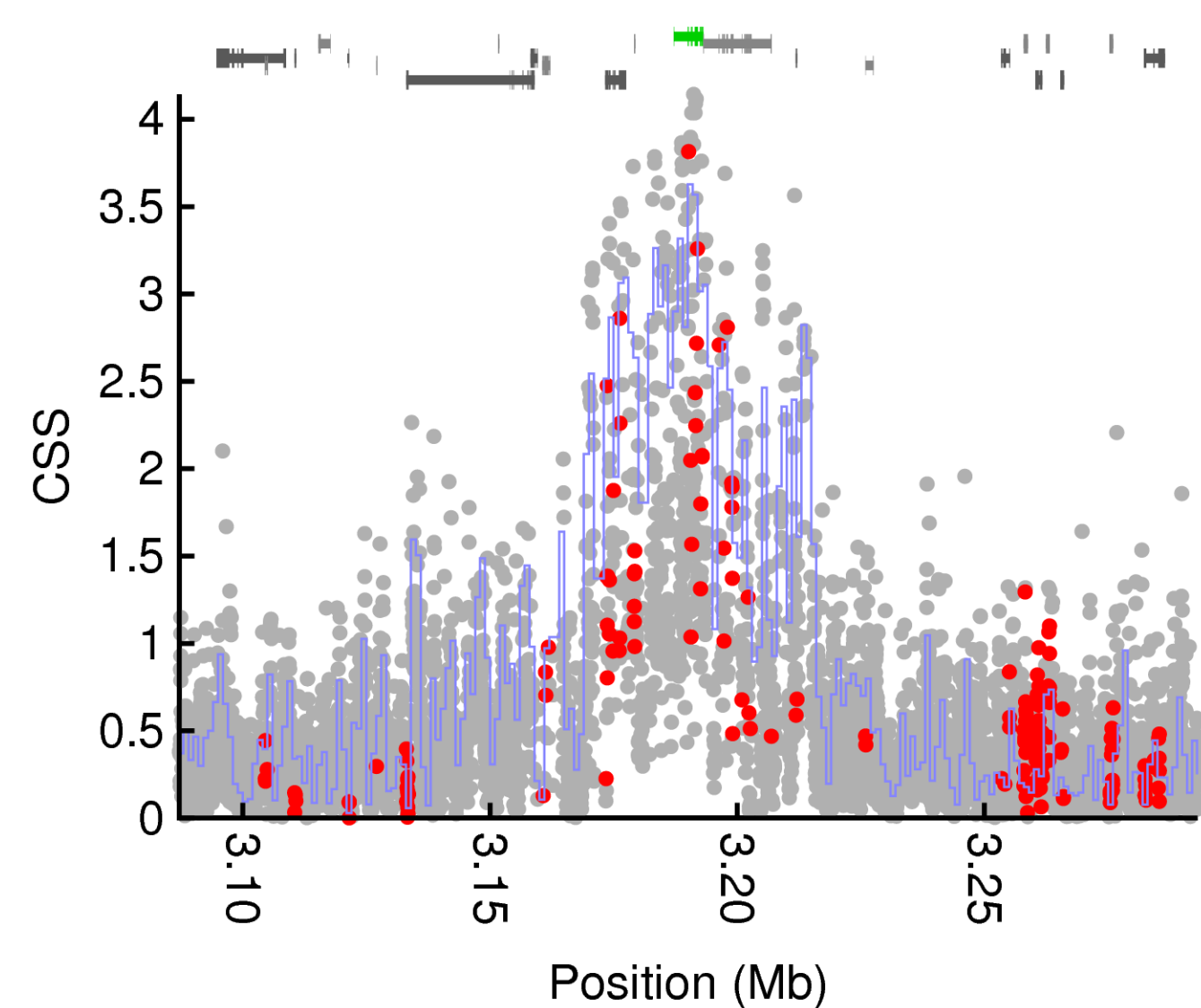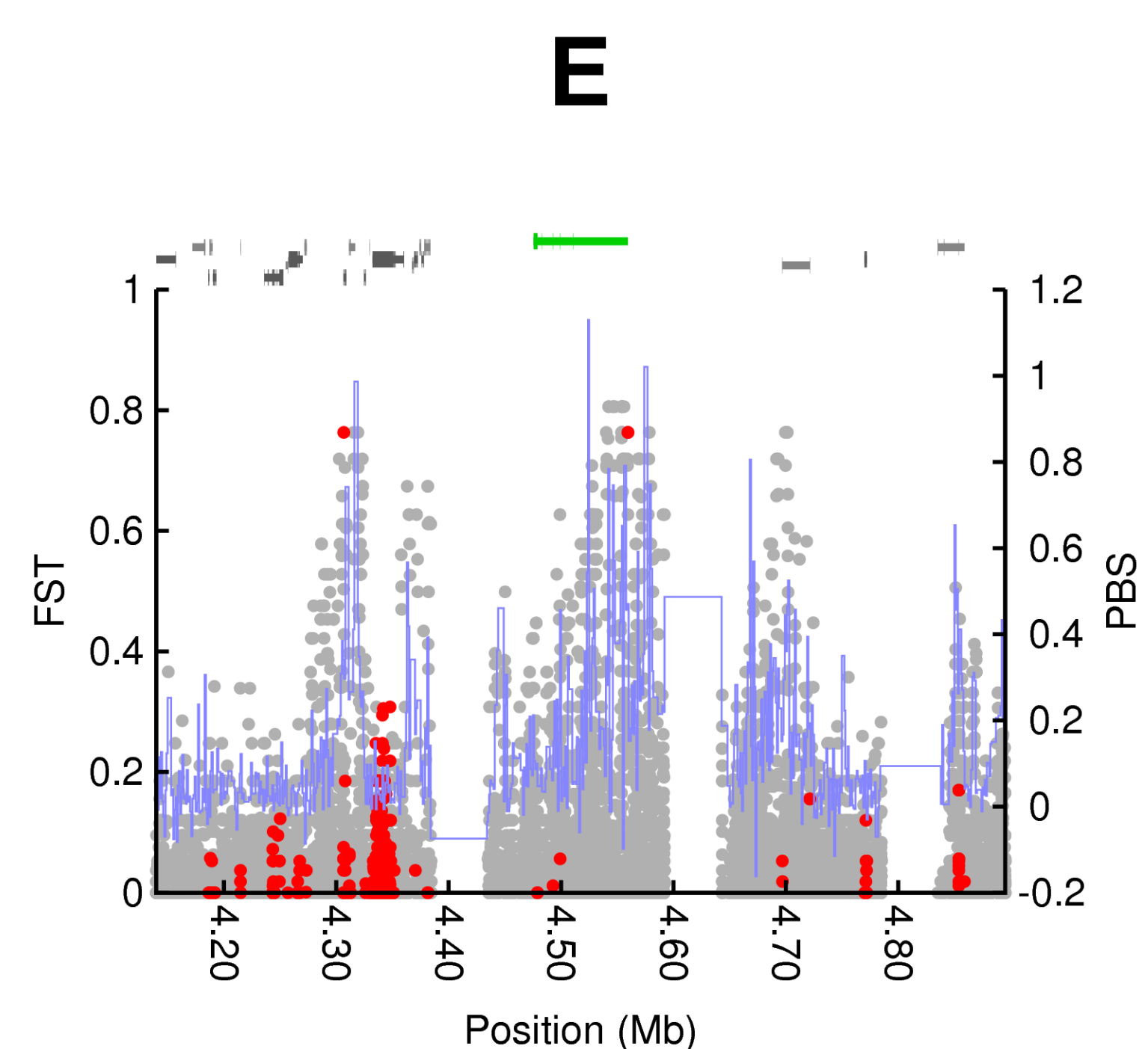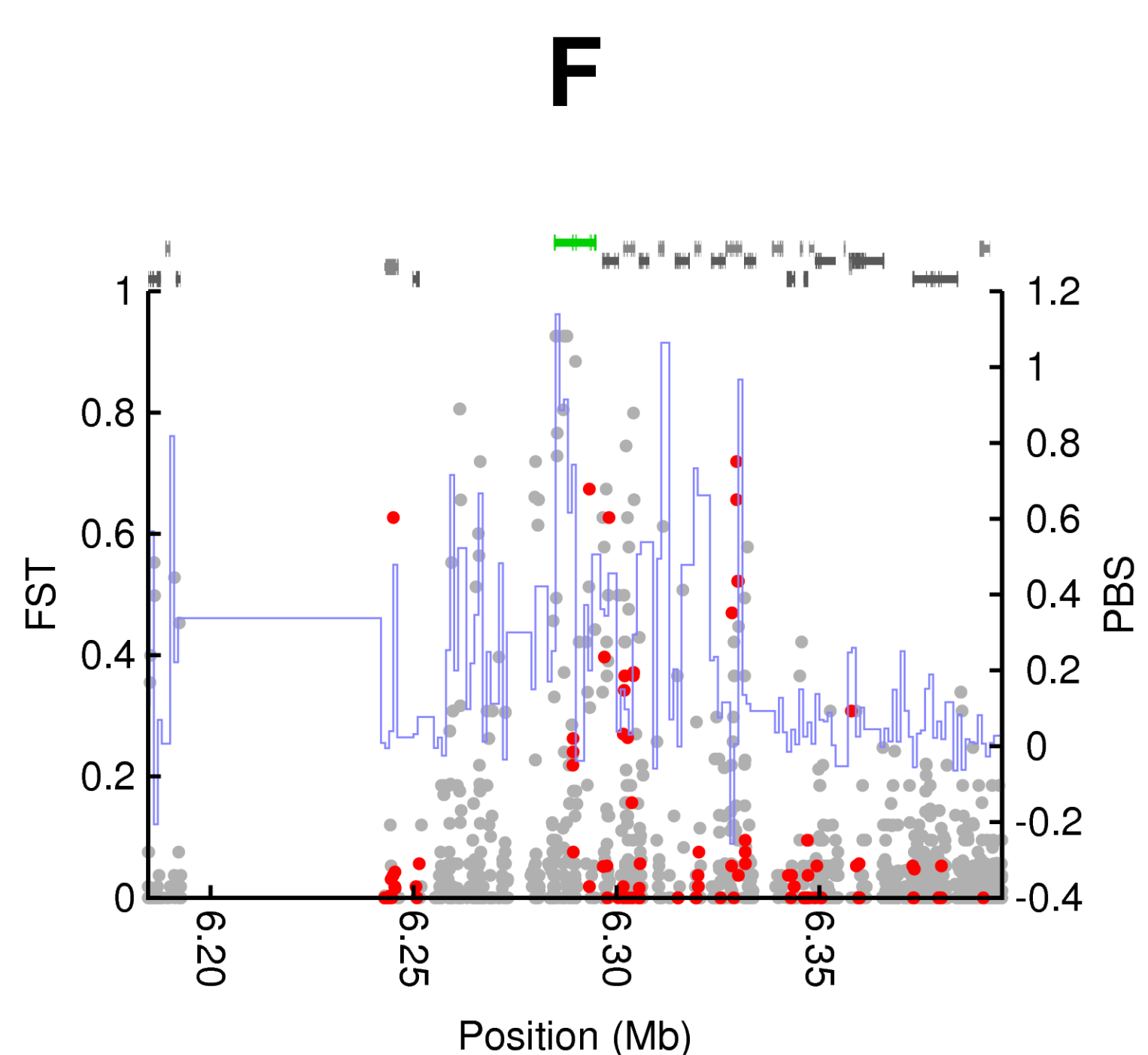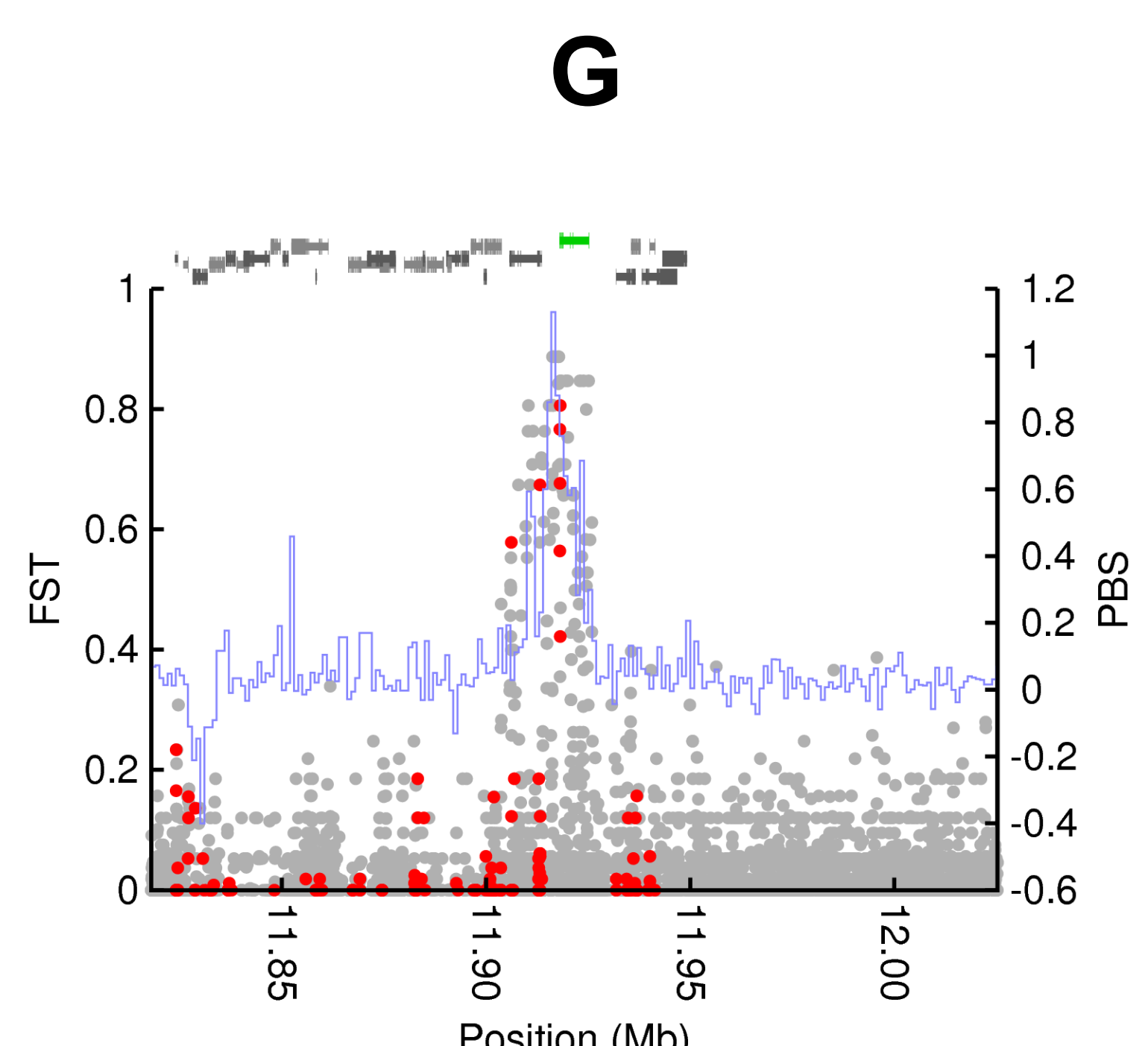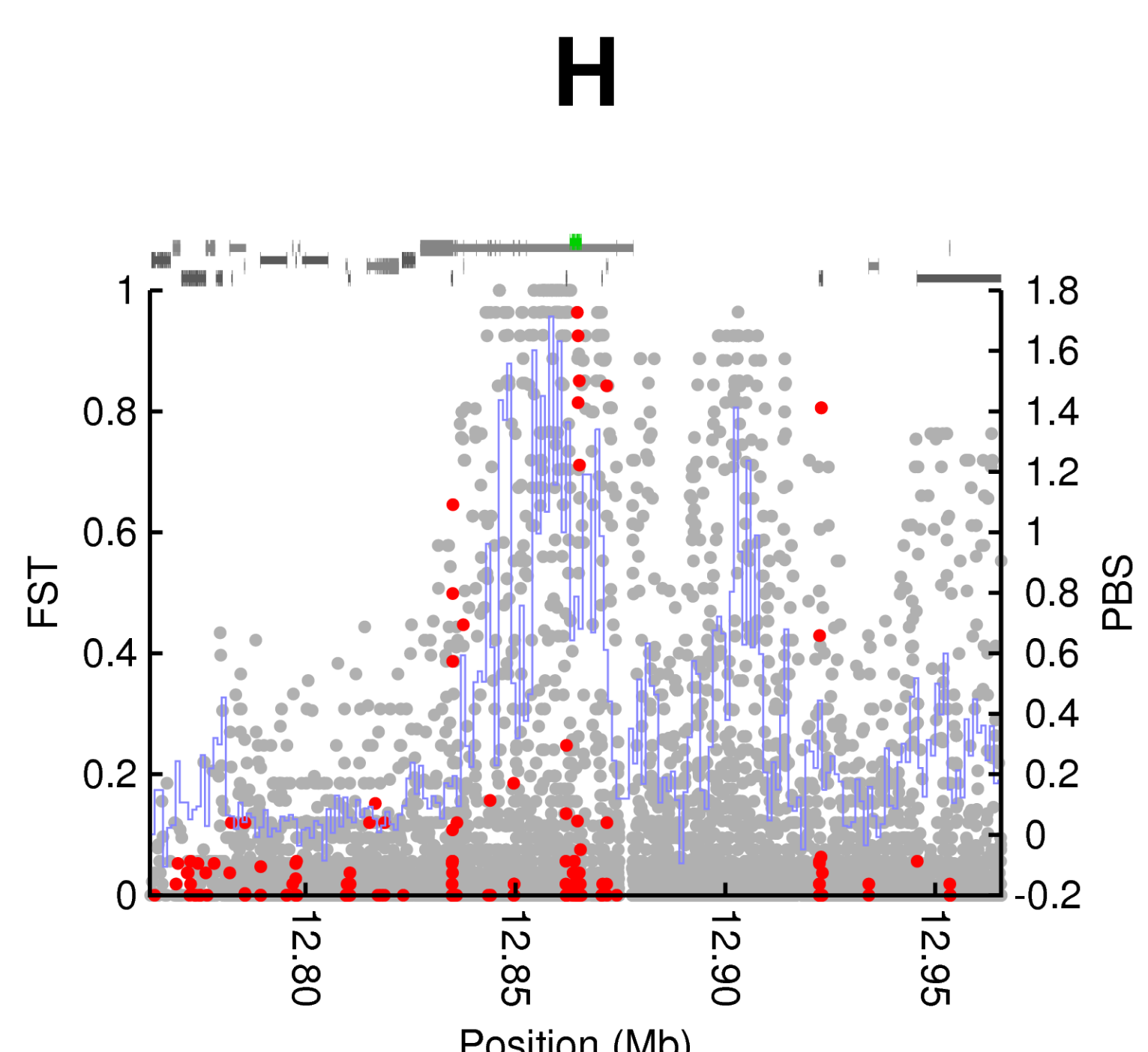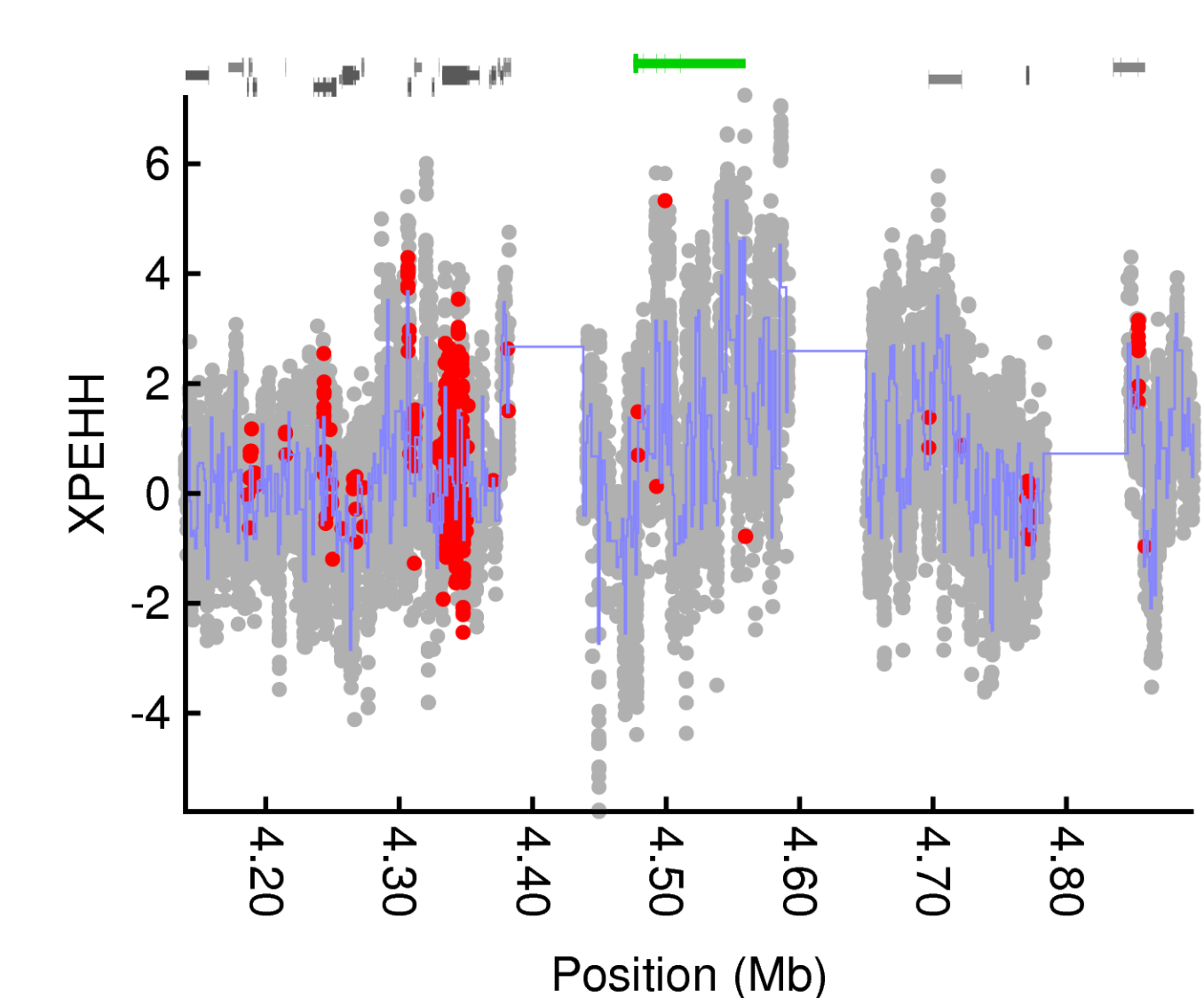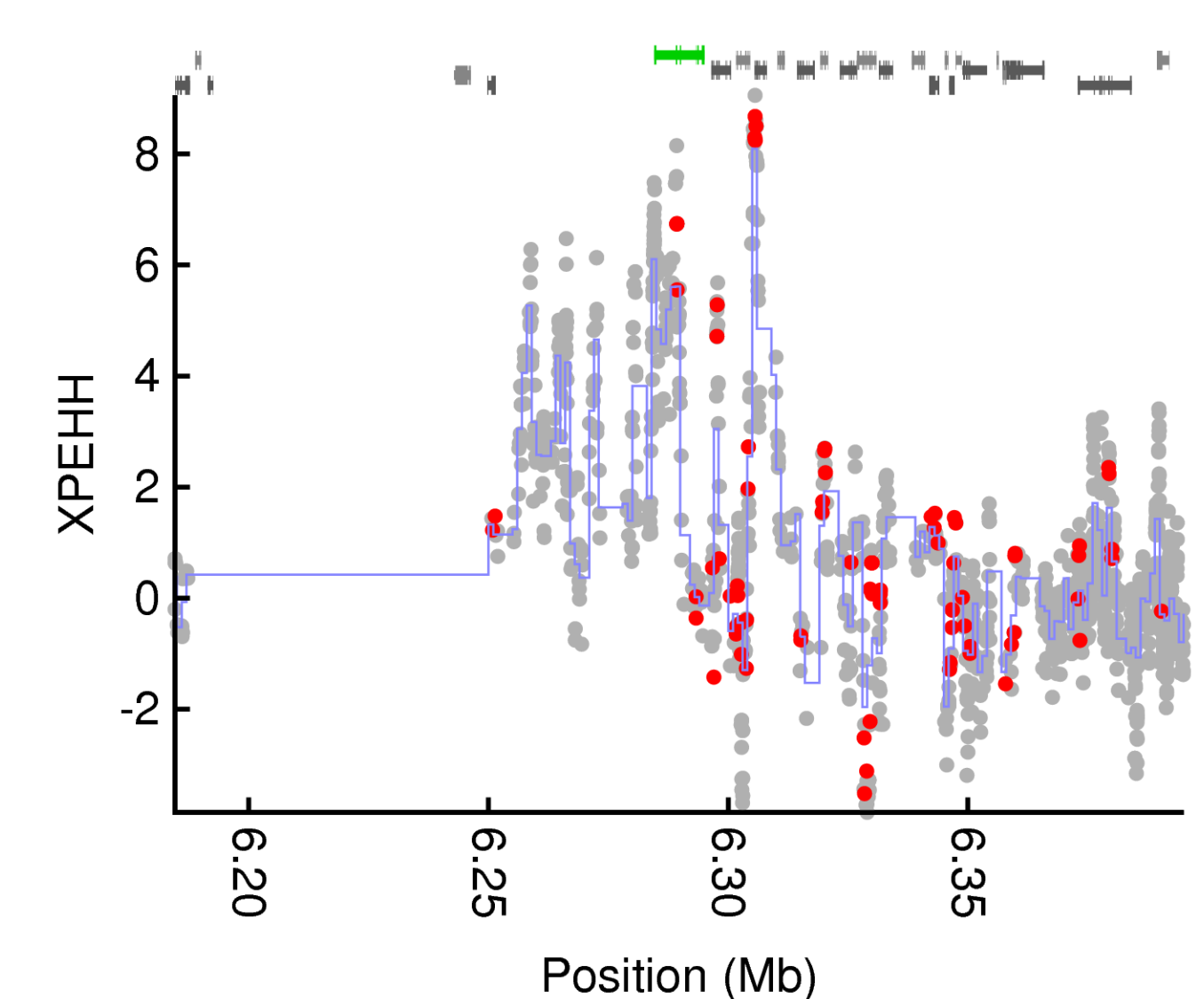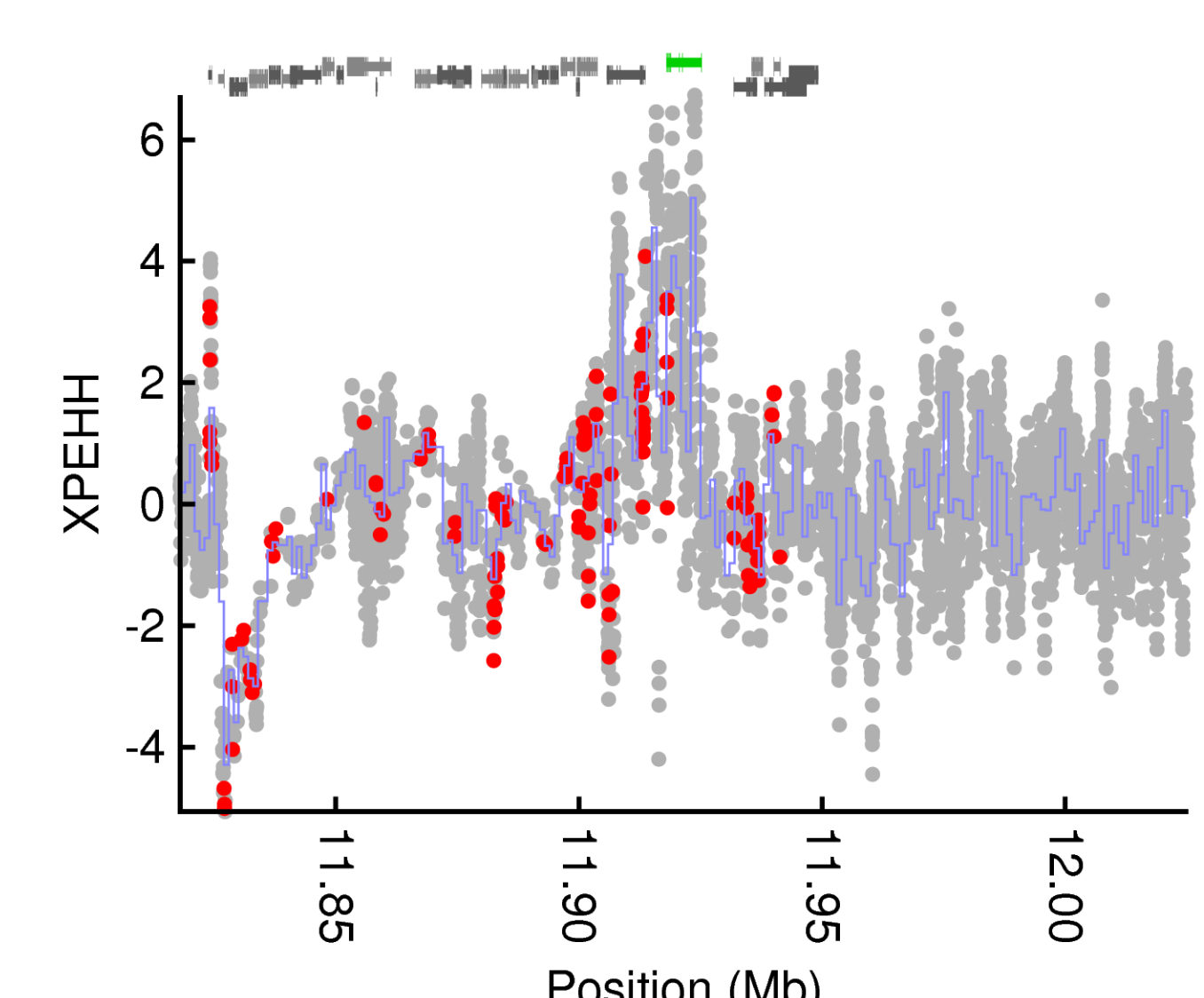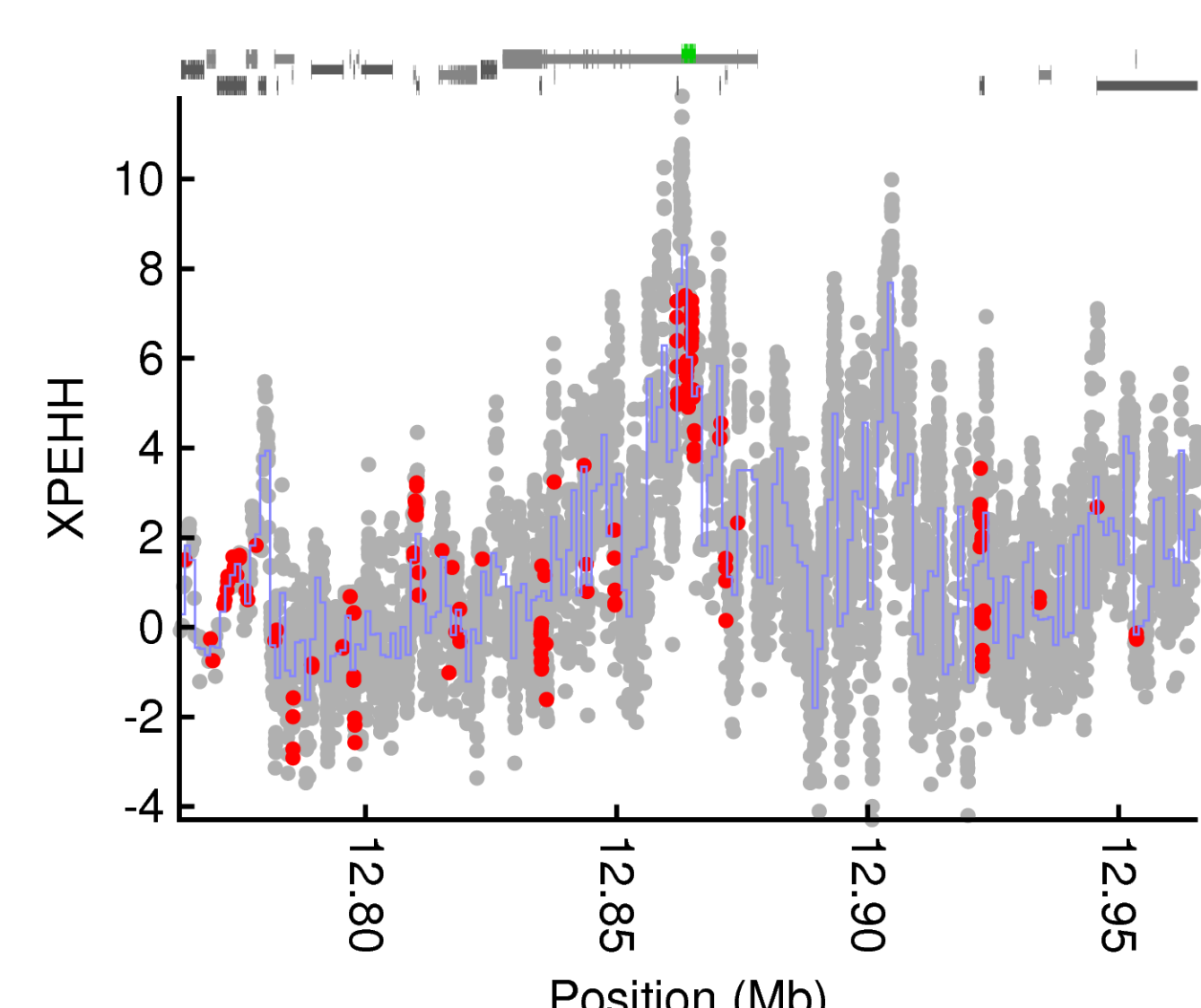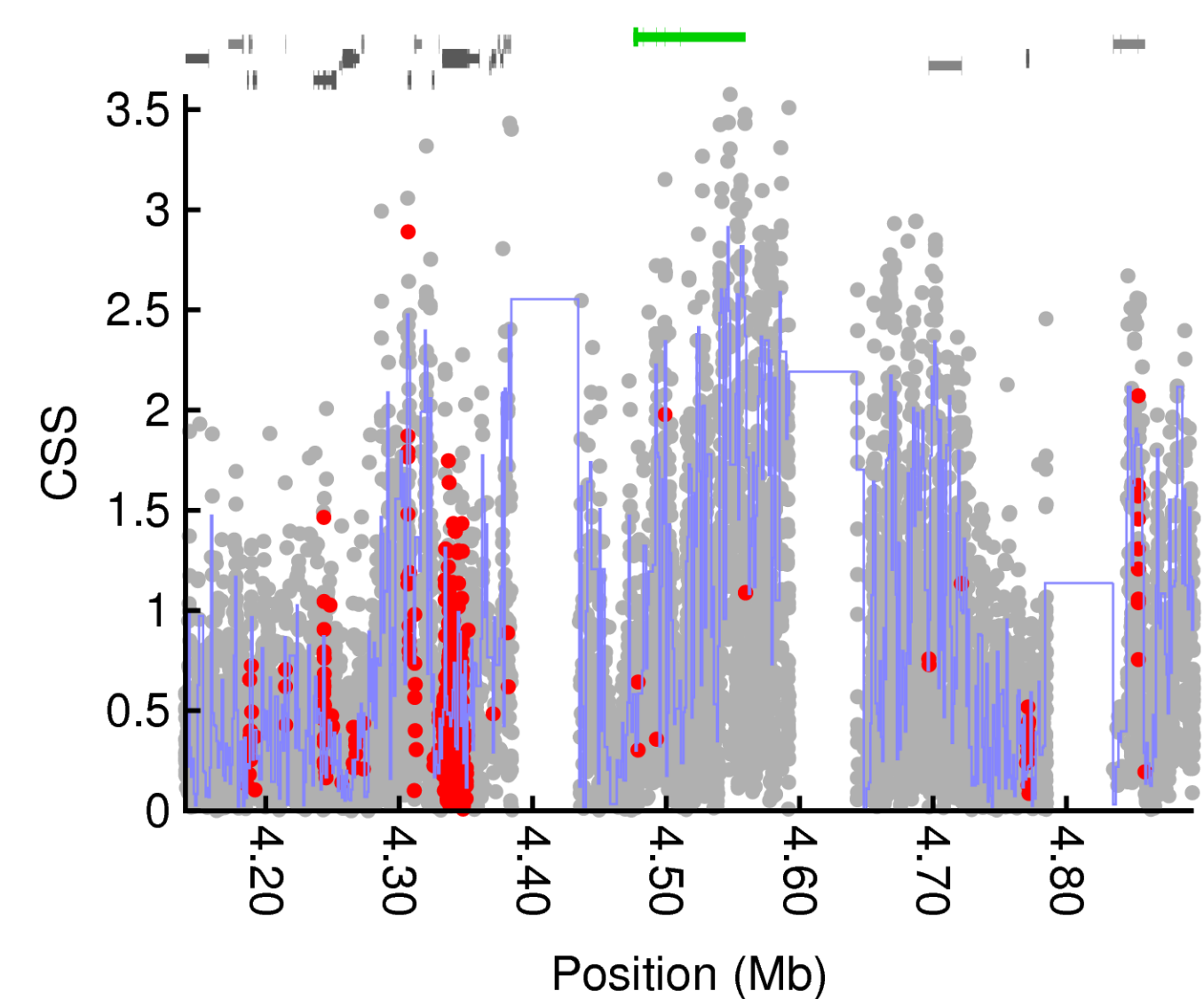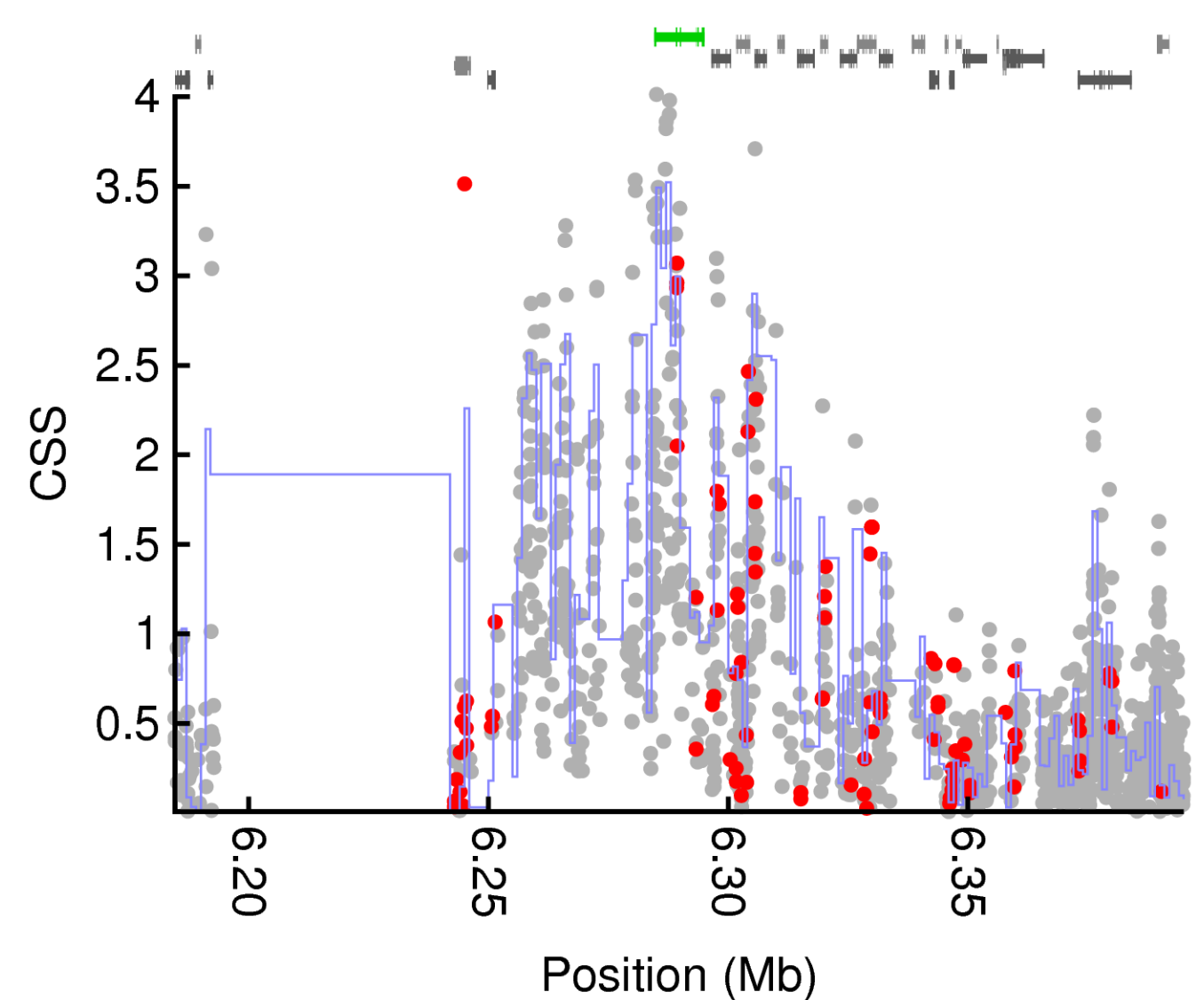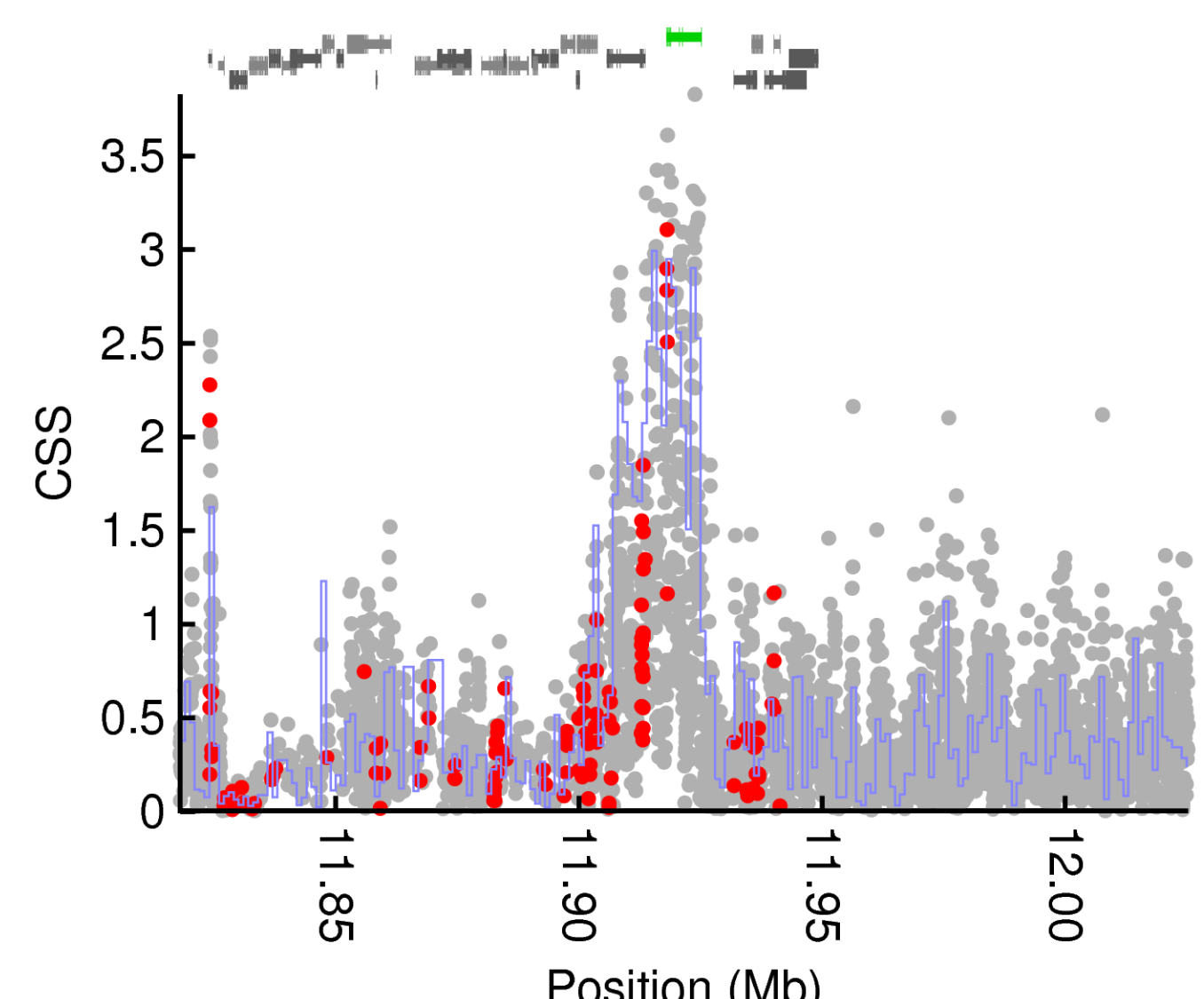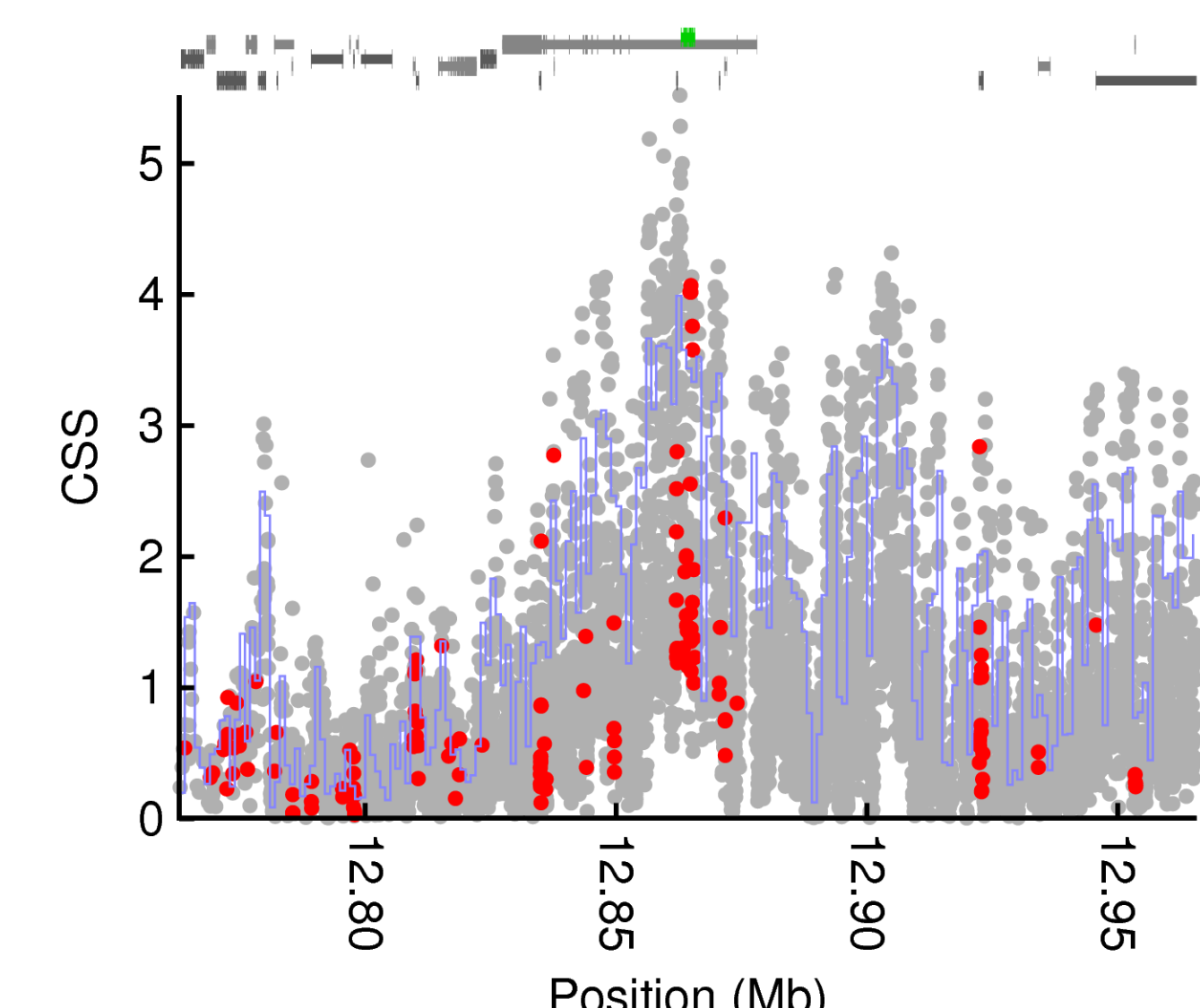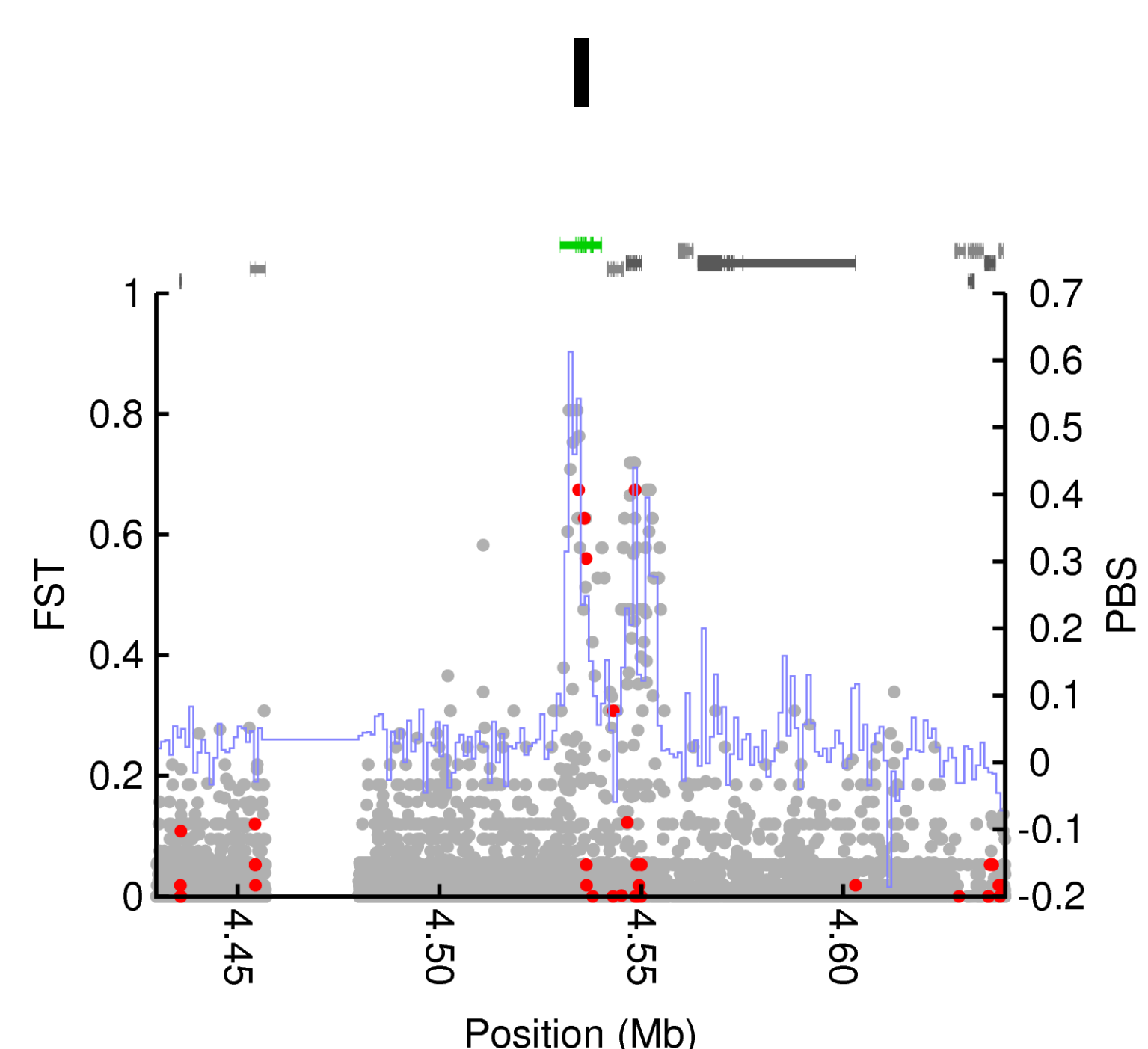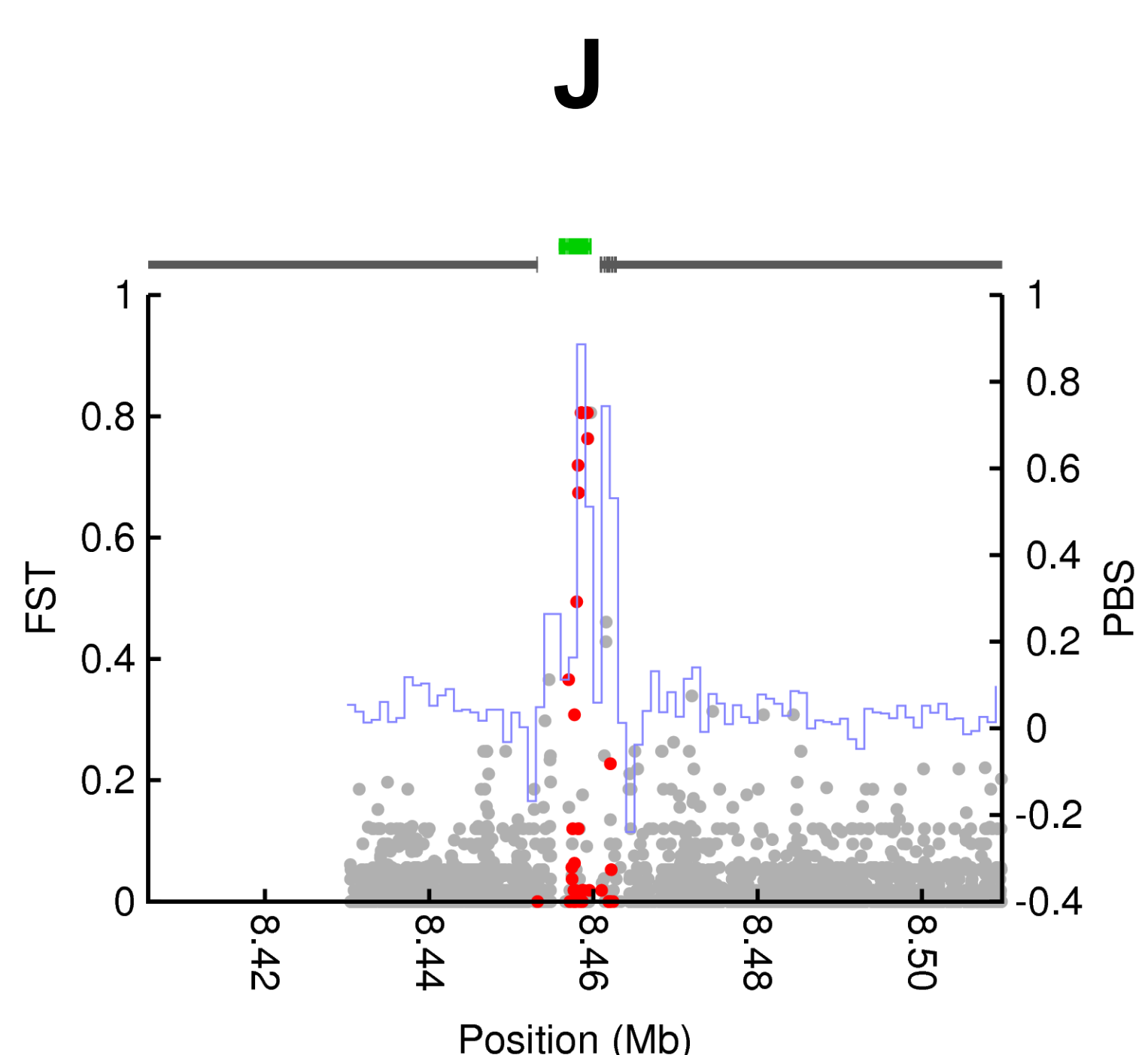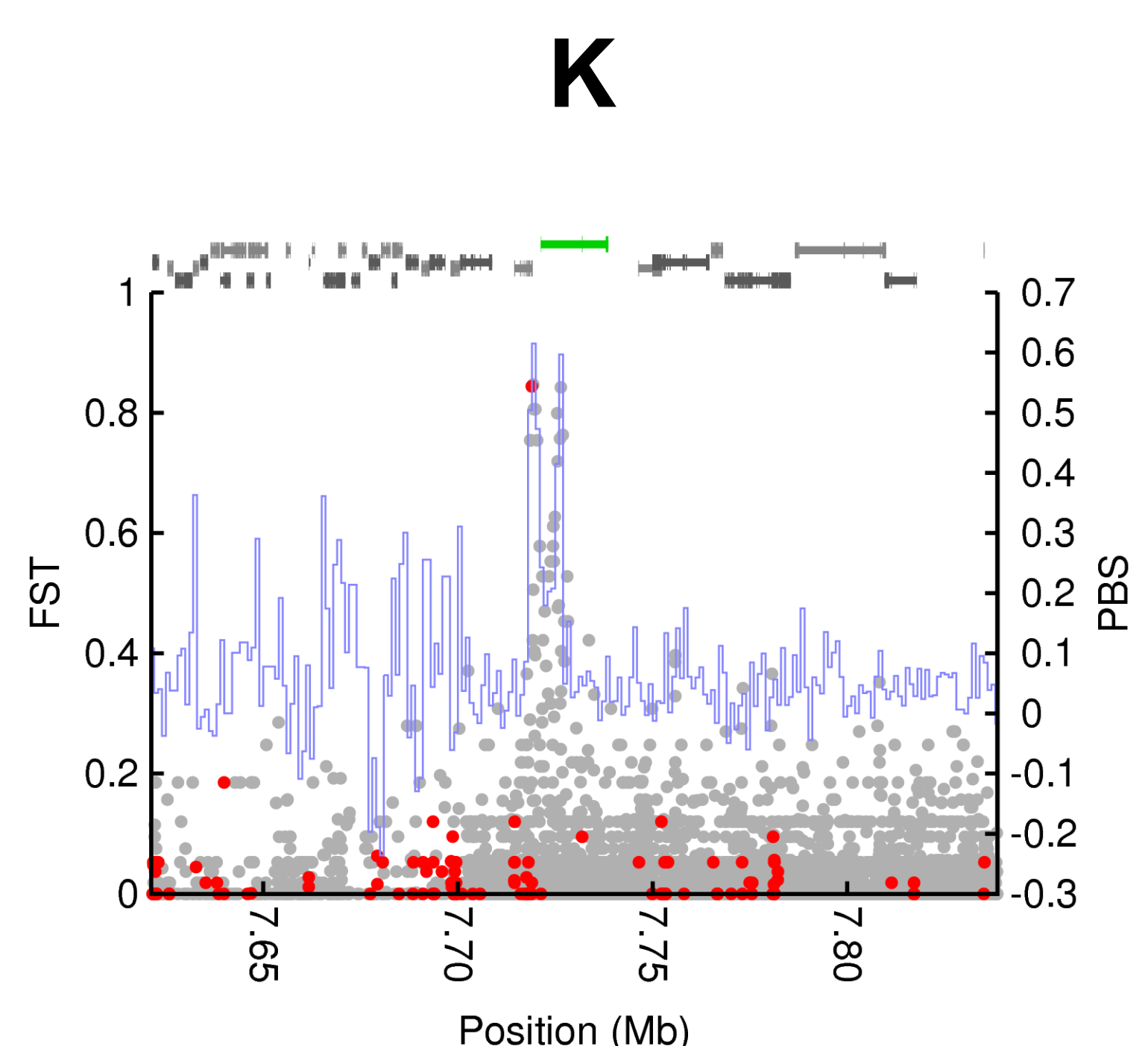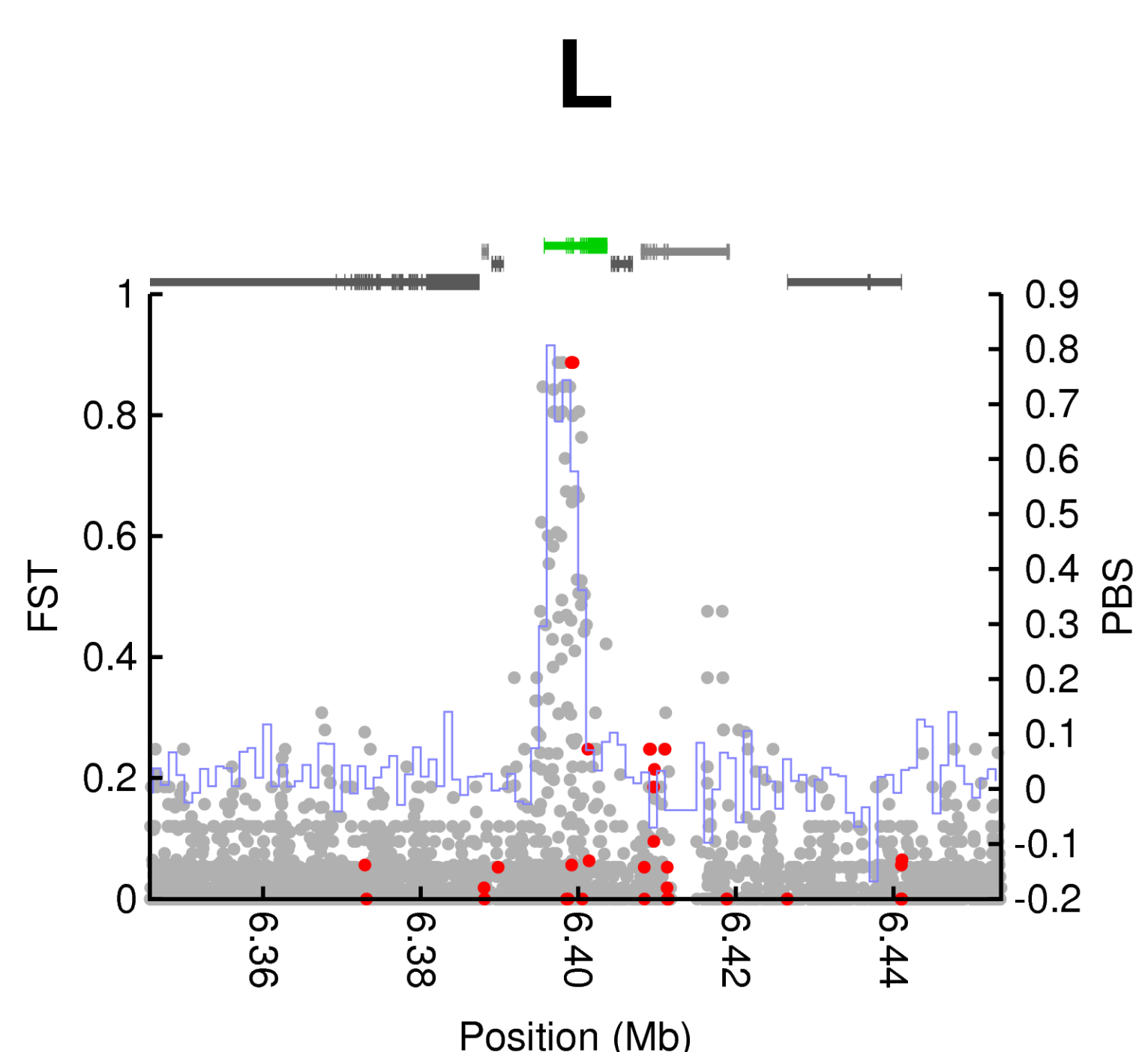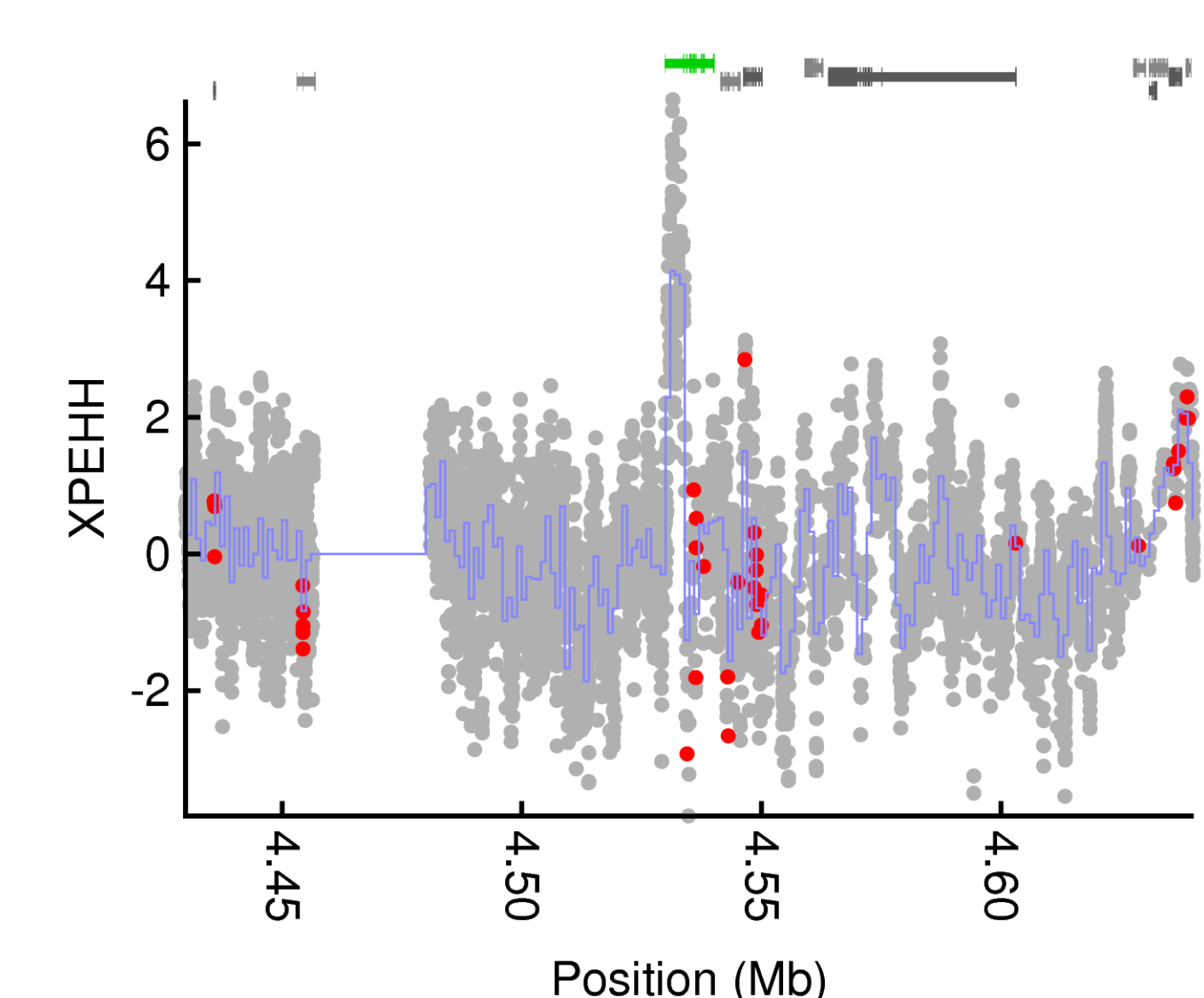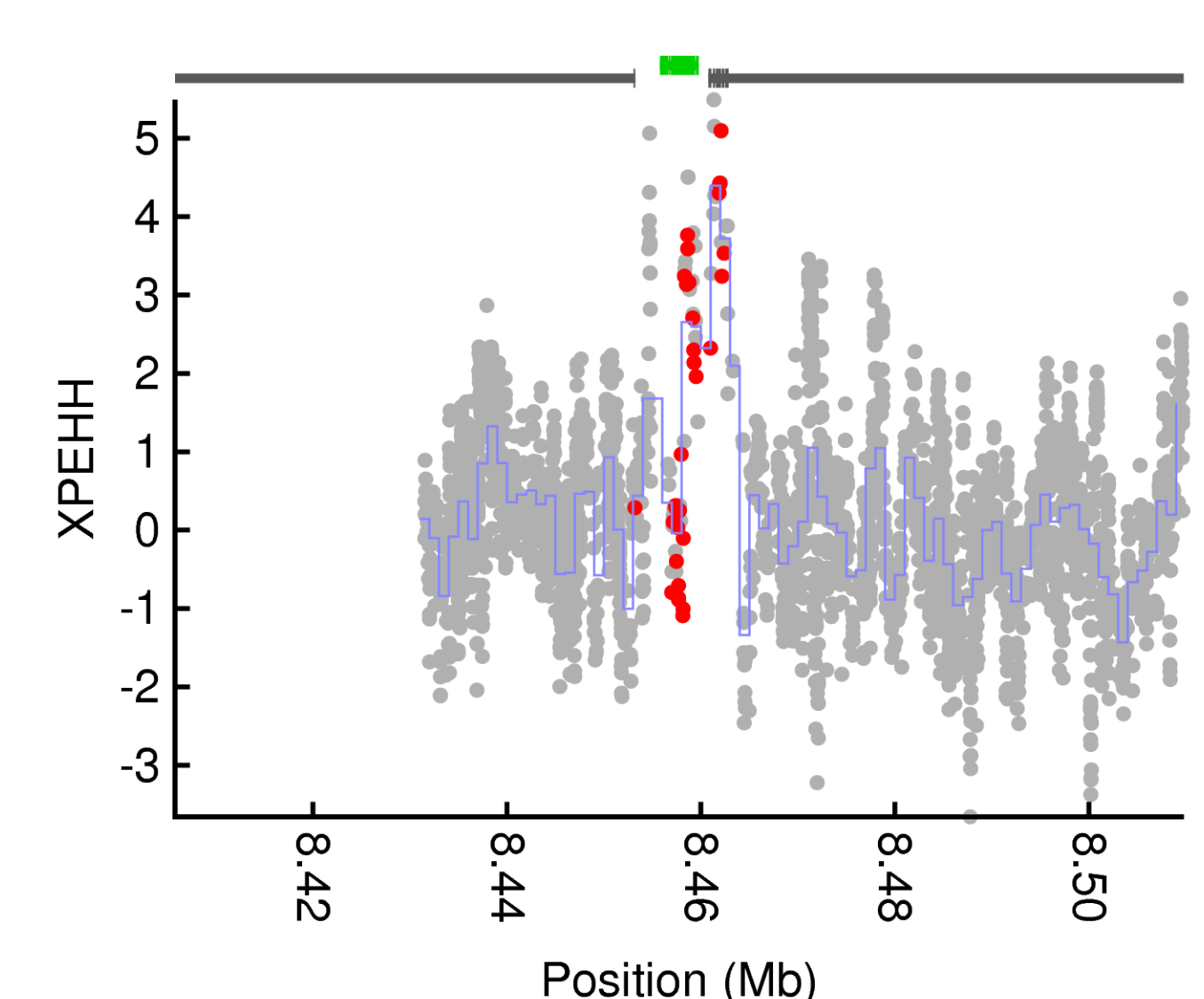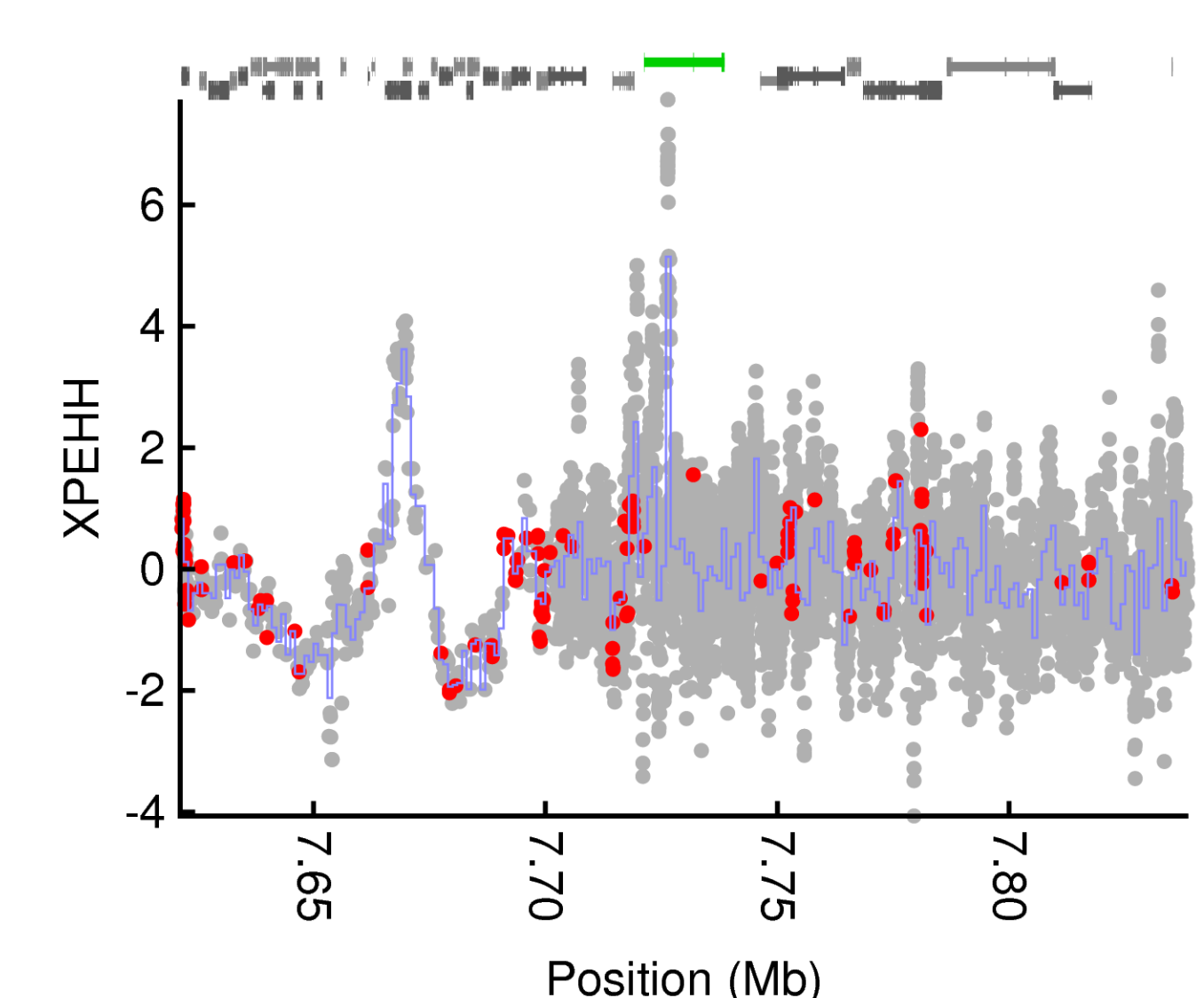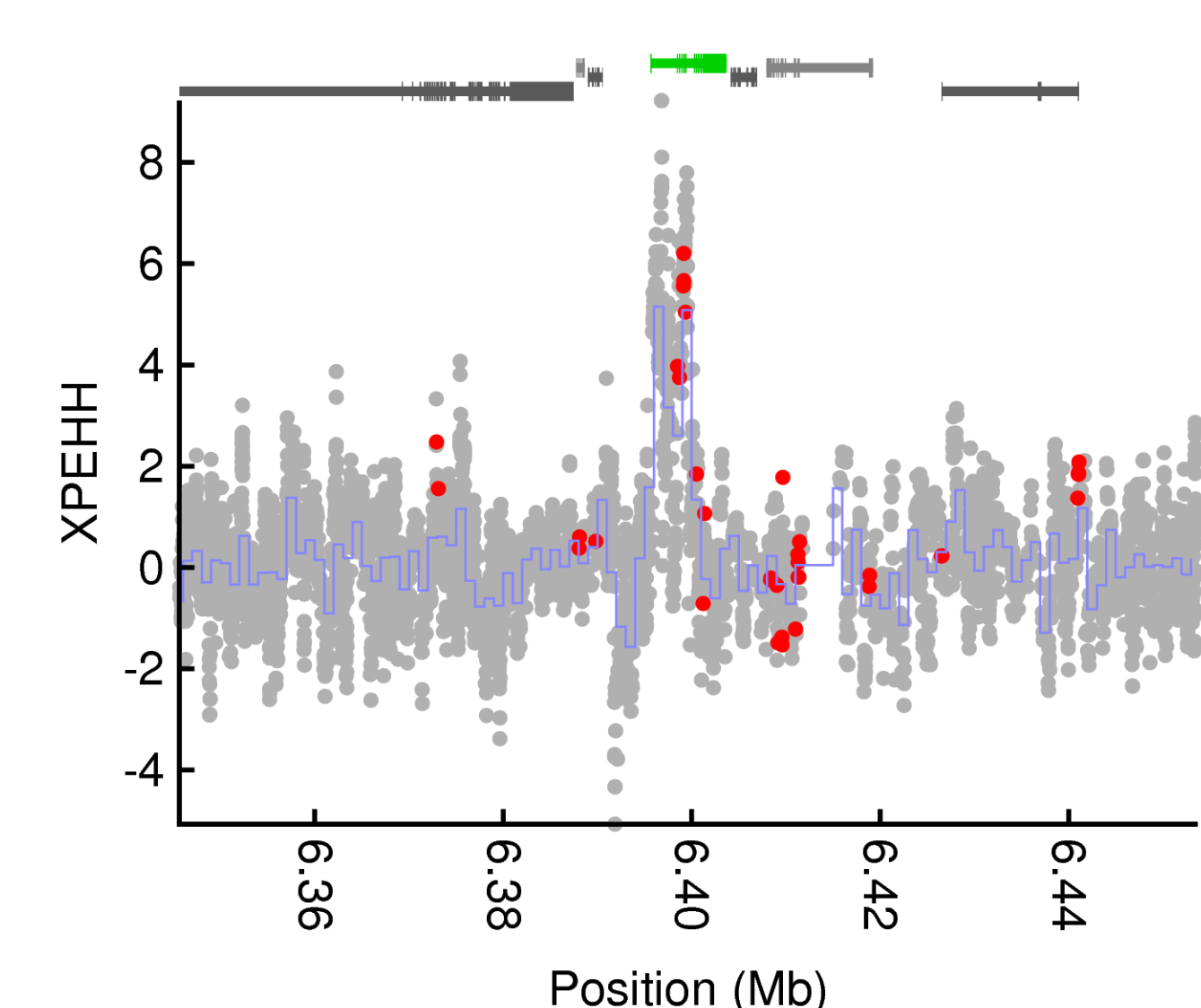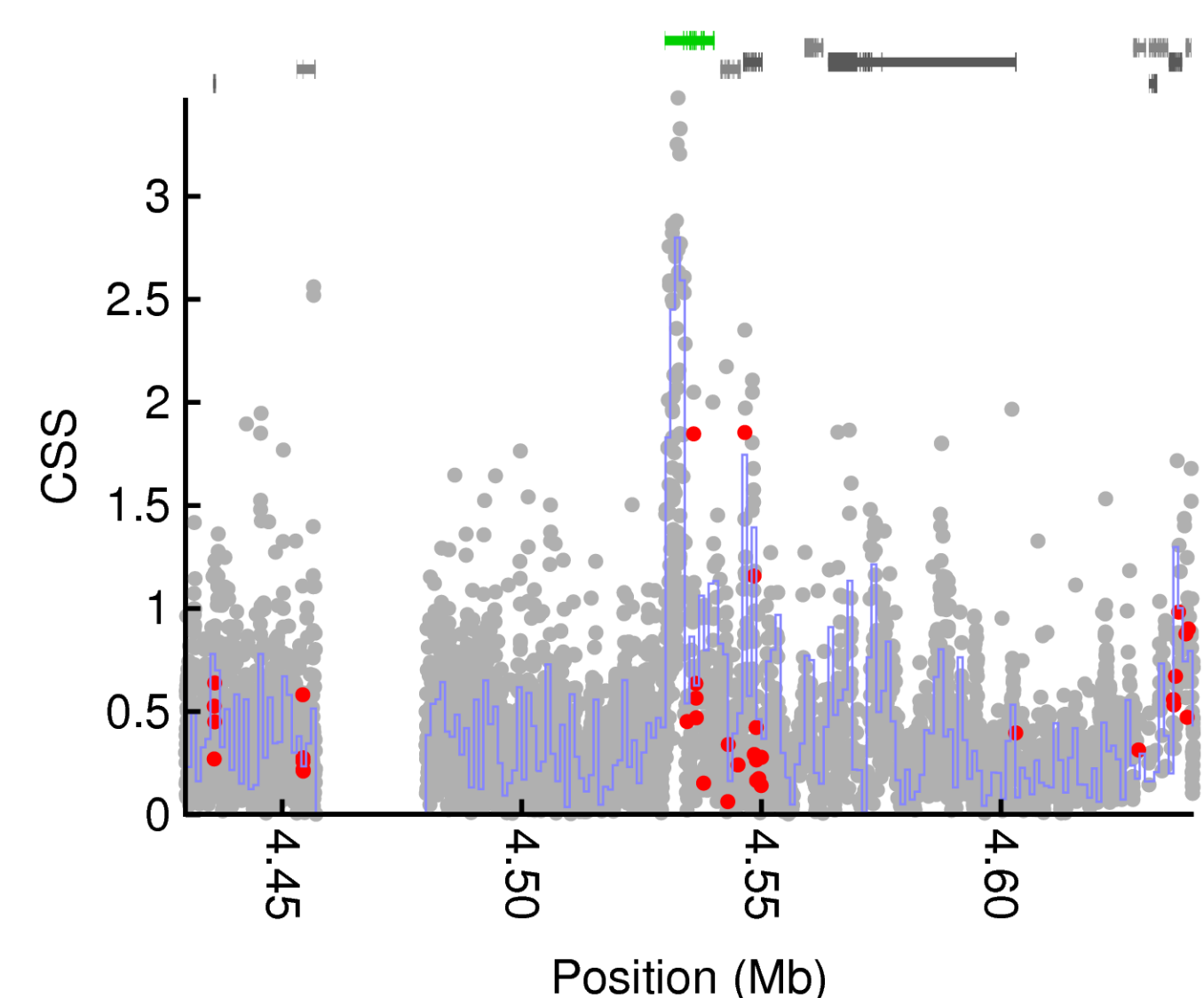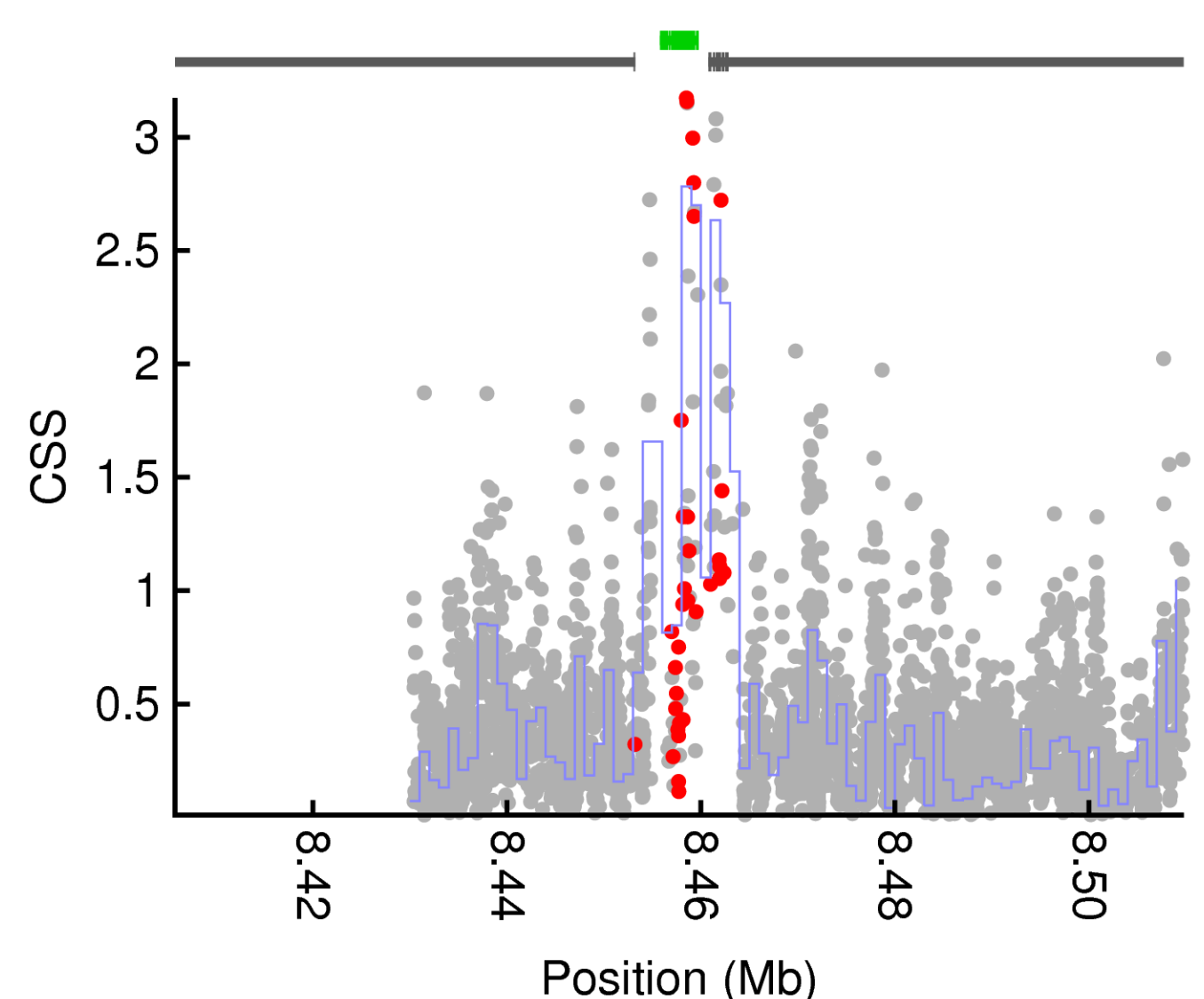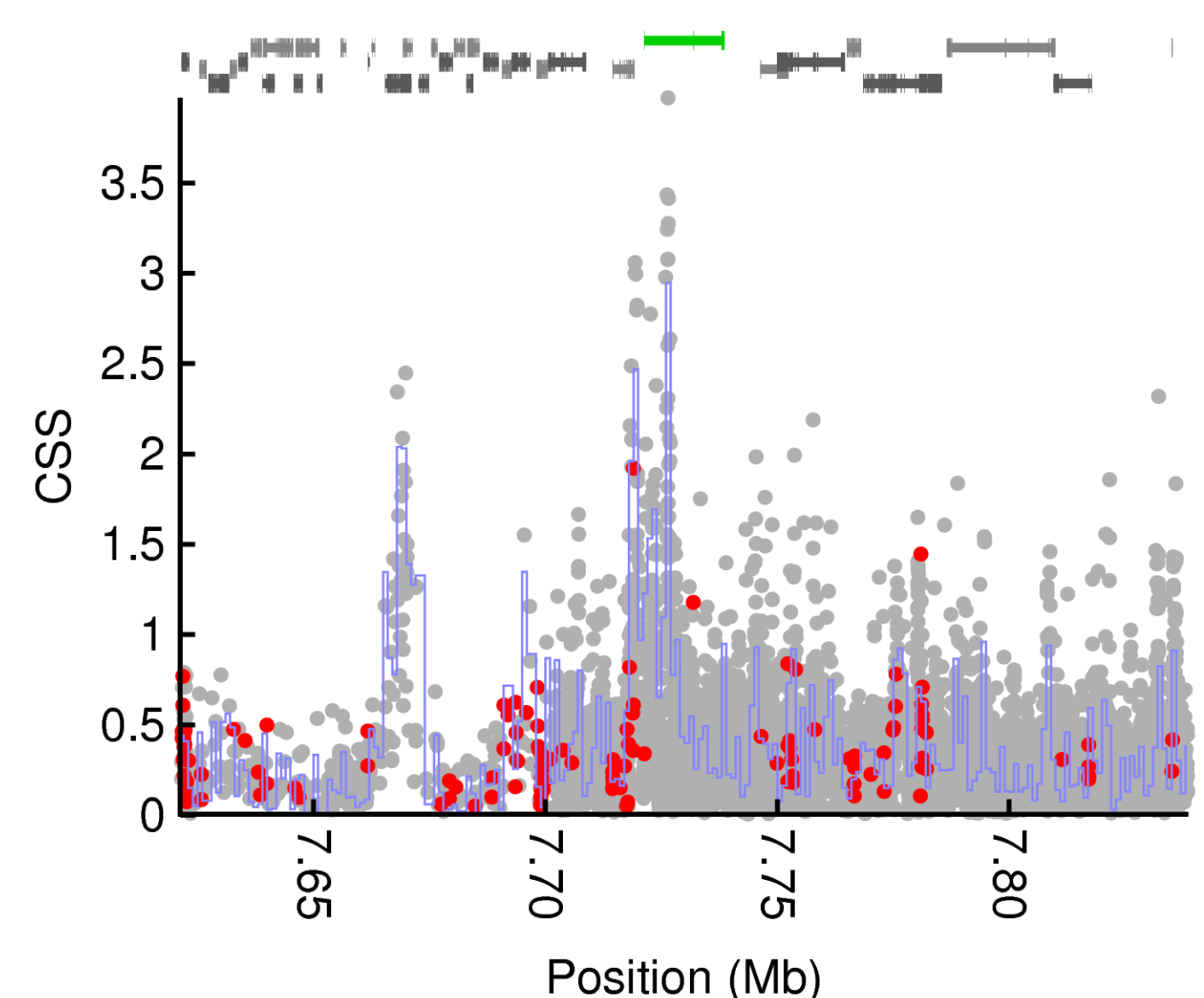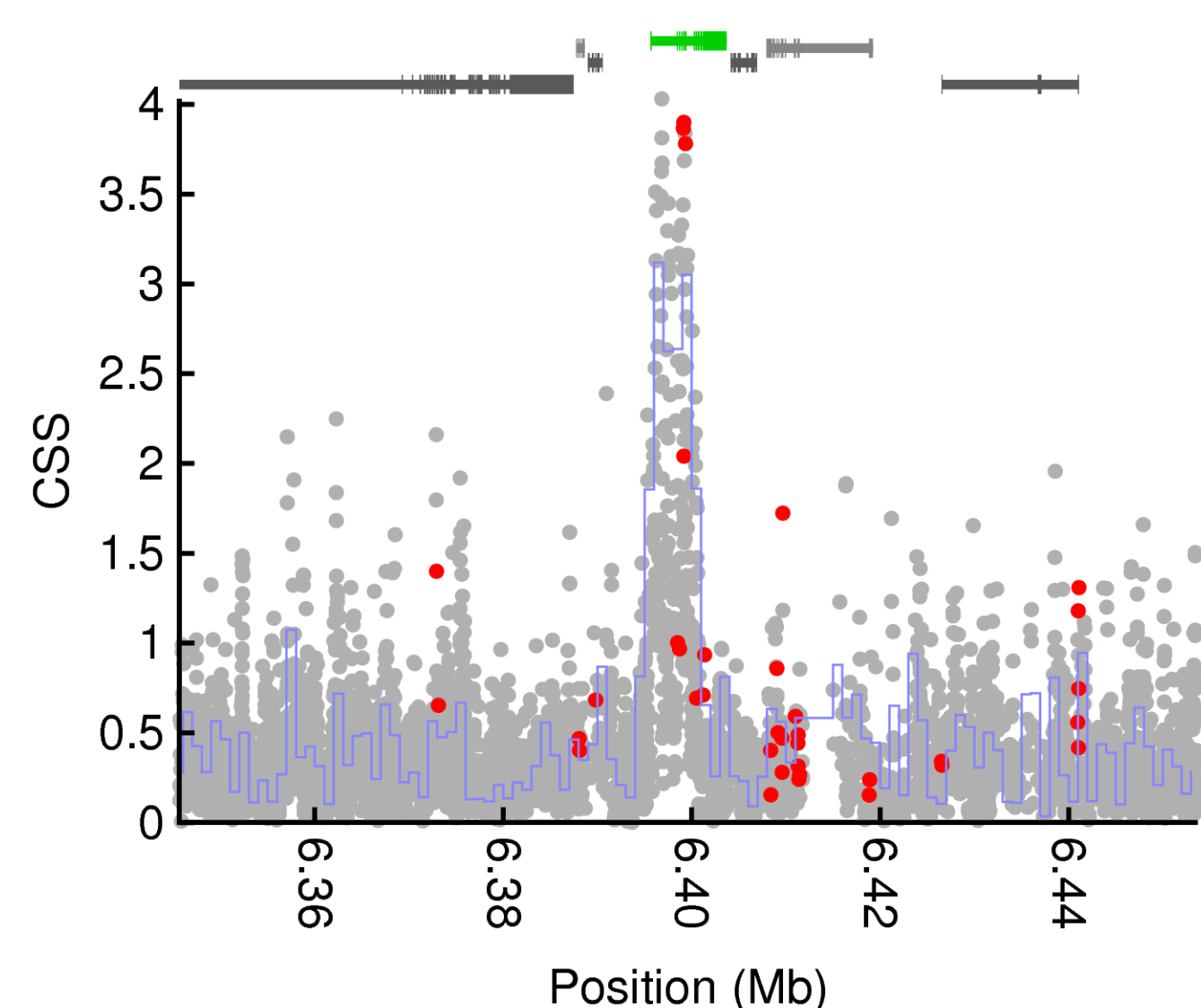

Supplement: S8 Fig — Among the 97 candidate accessions associated with the top 1000 SNPs ranked for the composite selection score (CSS) were 25 accessions that include at least one SNP above the 99.99% FST percentile (FST>0.80). These are located in 12 selective sweeps (labeled from A to L) across the genome. (A) Sweep A; around accession GB40769 (LOC412458; Dehydrogenase/reductase SDR family member 11-like). Top plot: FST of all SNPs in the region (grey dots); FST of non-synonymous SNPs (red dots), if detected; blue line is the population branch statistic PBS measured over 1kbp non-overlapping windows; gene bodies in grey above graph; the main accession is highlighted in green. Middle plot: XP-EHH of all SNPs in the region (grey dots); XP-EHH of non-synonymous SNPs (red dots); blue line is the mean XP-EHH measured over 1kbp non-overlapping windows. Genes as in the top plot. Bottom plot: the Composite Selection Score (CSS; from [24]) based on both FST and XP-EHH of all SNPs in the region (grey dots); CSS of non-synonymous SNPs (red dots); blue line is the mean CSS measured over 1kbp non-overlapping windows. Genes as in the top plot. (B) Sweep B; around accession GB46500 (Ethr; Ecdysis triggering hormone receptor). Plots as in (A). (C) Sweep C; around accession GB50742 (LOC102655146; Bardet-biedl syndrome 1 protein-like). Plots as in (A). (D) Sweep D; around accession GB54486 (LOC411978; β-glucosidase). Plots as in (A). (E) Sweep E; around accession GB54634 (LOC725260; Uncharacterized LOC725260). Plots as in (A). (F) Sweep F; around accession GB43519 (LOC411614; Neuropeptide y receptor-like). Plots as in (A). (G) Sweep G; around accession GB44980 (LOC409260; Epidermal retinol dehydrogenase 2-like). Plots as in (A). (H) Sweep H; around accession GB45239 (LOC100576557; Uncharacterized protein LOC100576557). Plots as in (A). (I) Sweep I; around accession GB40077 (LOC726040; Probable 4-coumarate—coA ligase 3-like). Plots as in (A). (J) Sweep J; around accession GB49919 (LOC724687; Uncharacte [file pgen.1006097.s008.pdf]
