# Supplementary material for: Identification of Multiple Loci Associated with Social Parasitism in Honeybees
Source: PLoS Genet. 2016 Jun 9;12(6):e1006097. doi: 10.1371/journal.pgen.1006097 (PMC4900560; doi:10.1371/journal.pgen.1006097)
Supplement: S9 Fig — Short read data was mapped across the putative 9bp deletion (the thelytoky associated element 1; tae1) in one of the introns of the transcription factor Gemini and visualized using samtools console output. (A) Read data for 10 capensis samples. The deletion element is located to position 1,532,307–1,532,315 on chromosome 13 (5’-TTCCATCGT-3’ on the plus strand; highlighted in black). Samples #4 and #5 have tae1-consistent reads without the motif mapped across the region. These are aligned with *-symbols indicating gaps in the alignment against the reference sequence. Samples #2 and #3 do not have well mapped data across the region and may also lack the element. (B) Read data for 10 scutellata samples. Samples #2 and #3 have tae1-consistent reads without the motif mapped across the region. Sample #10 does not have well mapped data across the region and may lack the element. (C) Read data for 10 adansonii samples. Samples #5 and #10 tae1-consistent reads without the motif mapped across the region. Samples #1 and #7 does not have well mapped data across the region and may lack the element. (D) Table summarizing the short read evidence for the occurrence of tae1 across the three subspecies. Reads aligned with gaps across the deletion motif were detected in 2 out 10 samples in all three subspecies. Additional samples may also have the deletion. The deletion is not fixed in the Cape bees. (PDF) [file pgen.1006097.s009.pdf]

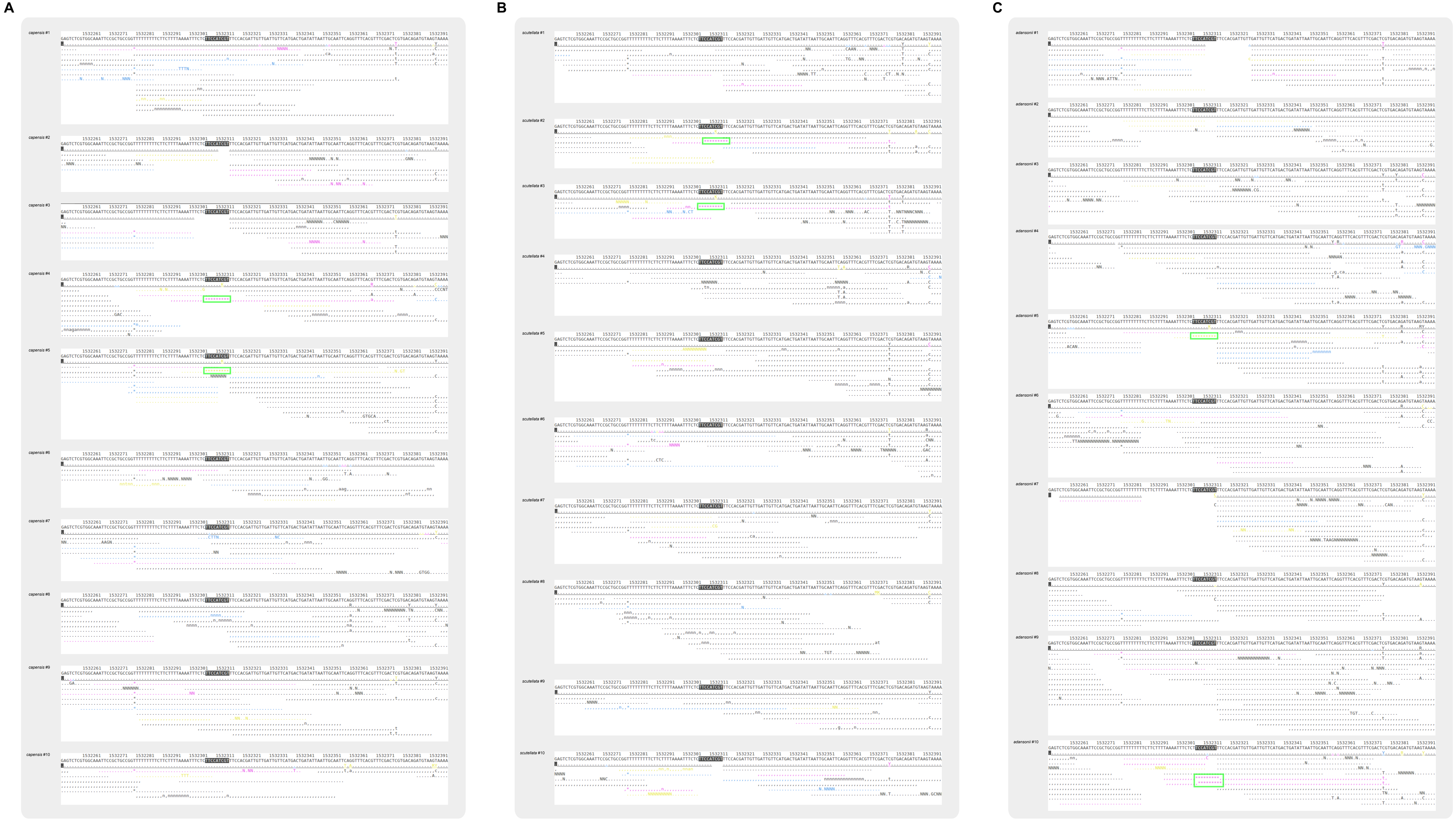

D

Subspecies	Sample	Deletion evidence	Putative genotype (D=deletion, d=no deletion)
capensis	1	well mapped reads w/o deletion	d
	2	no mapped reads	D
	3	no mapped reads	D
	4	alignment gap	D
	5	alignment gap	D
	6	well mapped reads w/o deletion	d
	7	well mapped reads w/o deletion	d
	8	well mapped reads w/o deletion	d
	9	well mapped reads w/o deletion	d
	10	well mapped reads w/o deletion	d
Population frequency			40%
scutellata	1	well mapped reads w/o deletion	d
	2	alignment gap	D
	3	alignment gap	D
	4	well mapped reads w/o deletion	d
	5	well mapped reads w/o deletion	d
	6	well mapped reads w/o deletion	d
	7	well mapped reads w/o deletion	d
	8	well mapped reads w/o deletion	d
	9	well mapped reads w/o deletion	d
	10	no mapped reads	D
Population frequency			30%
adansonii	1	no mapped reads	D
	2	well mapped reads w/o deletion	d
	3	well mapped reads w/o deletion	d
	4	well mapped reads w/o deletion	d
	5	alignment gap	D
	6	well mapped reads w/o deletion	d
	7	no mapped reads	D
	8	well mapped reads w/o deletion	d
	9	well mapped reads w/o deletion	d
	10	alignment gap	D
Population frequency			40%
